# *In Vivo* Angiogenesis Screening and Mechanism of Action of Novel Tanshinone Derivatives Produced by One-Pot Combinatorial Modification of Natural Tanshinone Mixture from *Salvia Miltiorrhiza*

**DOI:** 10.1371/journal.pone.0100416

**Published:** 2014-07-03

**Authors:** Zhe-Rui Zhang, Jin-Hang Li, Shang Li, Ai-Lin Liu, Pui-Man Hoi, Hai-Yan Tian, Wen-Cai Ye, Simon Ming-Yuen Lee, Ren-Wang Jiang

**Affiliations:** 1 State Key Laboratory of Quality Research in Chinese Medicine and Institute of Chinese Medical Sciences, University of Macau, Macao, P.R. China; 2 College of Pharmacy, Jinan University, Guangzhou, P.R. China; 3 Institute of Materia Medica, Chinese Academy of Medical Sciences and Peking Union Medical College, Beijing, P. R. China; University of East Anglia, United Kingdom

## Abstract

**Background:**

Natural products present in low quantity in herb medicines constitute an important source of chemical diversity. However, the isolation of sufficient amounts of these low abundant constituents for structural modification has been a challenge for several decades and subsequently halts research on the utilization of this important source of chemical entities for drug discovery and development. And, pro-angiogenic therapies are being explored as options to treat cardio-cerebral vascular diseases and wound healing recently. The present study investigates the pro-angiogenic potential of tanshinone derivatives produced by one-pot synthesis using zebrafish model.

**Methodology/Principal Findings:**

In order to address the difficulty of chemical modification of low abundant constituents in herb medicines, a novel one-pot combinatorial modification was used to diversify a partially purified tanshinone mixture from *Salvia miltiorrhiza*. This led to the isolation of ten new imidazole-tanshinones (Compounds **1**–**10**) and one oxazole-tanshinone (Compound **11**), the structures of which were characterized by spectroscopic methods in combination with single-crystal X-ray crystallographic analysis. The angiogenesis activities of the new tanshinone derivatives were determined in an experimental model of chemical-induced blood vessels damage in zebrafish. Of all the tested new derivatives, compound **10** exhibited the most potent vascular protective and restorative activity with an EC_50_ value of 0.026 µM. Moreover, the mechanism underlying the pro-angiogenesis effect of **10** probably involved the VEGF/FGF-Src-MAPK and PI3K-P38 signalling pathways by gene expression analysis and a blocking assay with pathways-specific kinase inhibitors.

**Conclusions/Significance:**

Taken together, our study demonstrated the more distinctive pro-angiogenic properties of **10** than other tanshinones and revealed **10** has potential for development as a pro-angiogenic agent for diseases associated with insufficient angiogenesis. Our results highlighted the great potential of adopting a newly modified one-pot approach to enhance the chemical diversity and biological activities of constituents from natural products regardless of their abundances.

## Introduction

Over 50% of commercial available drugs originated from the optimization of lead compounds isolated from natural products [1]. However, most reported chemical modification studies have mainly focused on the abundant compounds present in natural products as current organic synthesis technology does not efficiently modify small amount of compounds as starting materials. Thus, in the past, these minor compounds which have a higher structural diversity than the major compounds in natural products have been overlooked [2]. The development of an alternative novel approach is needed to address the bottleneck in the structural modification of these minor natural constituents.

Recently, there has been increasing interest in the application of the one-pot synthesis approach to create chemical structural diversity, in which chemical reactions were carried out by mixing starting materials including parental compounds and reactants into a single container to undergo multiple chains of modifying reactions without isolating intermediates. One-pot synthesis is also regarded as “green” mild approach as it improves the efficiency of chemical reactions and minimizes chemical waste generation by eliminating complicated separation and purification processes of the intermediate chemical compounds. For instance, even compounds with complex structure and multiple stereocentres, such as Oseltamivir, an anti-viral drug which is regarded as the last resort for controlling H5N1 infuenza pandemic, is successfully produced by sequential one-pot syntheses [3]. Interestingly, one-pot chemical diversification has been performed on an inactive natural product mixtures; however, only one semi-synthetic pyrazole was isolated [4]. A proof of concept study is necessary to fully utilize the chemical diversity of natural products in herbal medicine and generate an array of drug-like derivatives for subsequent screening using a partial fractionated chemical mixture instead of a pure compound as a starting material. Thus, both the major and minor constituent would be diversified simultaneously, and the conventional intensive labour and inefficient chemical isolation processes could be bypassed.

*Salvia miltiorrhiza* (Danshen), is widely used botanical material in traditional Chinese medicine for treating cerebral and cardiovascular diseases such as stroke [5], coronary heart disease [6] and hyperlipidemia [7]. Tanshinones are naturally occurring abietane-type diterpenoid naphthoquinone, which exist widely in *Salvia* species and could be considered as a natural combinatorial library [8], [9]. More than forty tanshinones have been isolated and identified. For instances, tanshinone IIA (Tan IIA, 0.29%), cryptotanshinone (CPT, 0.23%), tanshinone I (Tan I, 0.11%) are regarded as the major constitutes [10], [11], [12] while tanshindiol C, tanshinone VI, trijuganone A are examples of minor tanshinones [13]. However, structural modifications were exclusively carried out on the major tanshinones to improve their potency, solubility and bioavailability through a variety of modifications such as sulfation [14], acylation [15] and dimerization [16]. There has been no reported research on the modification of minor tanshinones. In particular, low abundant tanshinones in *Salvia miltiorrhiza* remain an unexplored natural source of starting materials for the discovery and development of new therapeutics.

Angiogenesis is a complex and critical step of various physiological and pathological processes. Many diseases such as cancers, psoriasis associated with excessive angiogenesis [17], [18]. Nevertheless, insufficient angiogensis also occurs in numerous human disorders (e.g. stroke and myocardial infarction) due to dysfunctional blood vessel formation [19], [20], [21]. Angiogenesis therapies focused on the unique medical approachs to help people lead healthier lives by restoring balance to blood vessels growth. Thereinto, pro-angiogenic therapies are being explored as options to treat cardio-cerebral vascular diseases and wound healing. Therefore, improvement in endothelial cells function and enhancement of the *de novo* blood vessels formation are important steps in the recovery process following severe ischemic and traumatic situations [22].

One of major limitations of commonly used *in vitro* assays which can precisely reflect the pharmacodynamic change of a specific mode of action to a specific cell type and/or molecular target, is that these assays do not facilitate the assessment of absorption, distribution, metabolism, and excretion (ADME) [23]. Recently, zebrafish (*Danio rerio*) has become an ideal environmentally friendly and economical *in vivo* drug screening model for improving various drug-like properties such as pharmacodynamic, absorption and metabolism simultaneously [24]. In addition, our previous study suggested that phase I and II drug metabolism systems in zebrafish are highly conserved compared with those in rodents and mammals [25]. Furthermore, zebrafish is highly similar to human at the anatomical and physiological levels; therefore many human diseases including cardiovascular diseases [26], cancer [27], leukemia [28], have been successfully reproduced in zebrafish. More importantly, the studies on pharmaceutical and biomedical discovery and developement in zebrafish can provide safer commerial products by bridging the gap in the translation of a scientific hypothesis to a human compatiable prototype [29]. The *Tg(fli-1a:EGFP)y1* zebrafish embryo provides a powerful tool for the discovery of novel angiogenesis modulating agents allowing observation of the responses of live embryo to drugs in real time. Moreover, our previous study established a novel chemical-induced blood vessels loss model which can mimic certain pathological conditions relating to angiogenesis deficiencies. Thus, a more physiologically relevant assay is required for identifying pro-angiogenesis agents against diseases asscoiated with angiogenesis deficiencies [30]. The aim of the present study was to identify new angiogenic tanshinone derivatives from the chemically modified total tanshinone extract of *Salvia miltiorrhiza* using the zebrafish angiogenesis assays.

In present study, new tanshinone derivatives were synthesized by mixing total natural tanshinones with reactants using a one-pot synthesis approach. A series of new tanshinone derivatives were isolated and subjected to both structural elucidation and zebrafish angiogenesis assays. Furthermore, the involvement mechanism underlying the angiogenesis effect of the new tanshinone derivatives was investigated using real-time qPCR analysis and testing for blocking effects with pathway-specific inhibitors.

## Materials and Methods

### General Remarks in Chemistry Assays

Ultraviolet (UV) spectra were determined in CH_3_Cl_3_ on a Jasco V-550 UV/vis spectrophotometer. ESI-MS spectra were carried out on a Finnigan LCQ Advantage Max ion trap mass spectrometer. HR-ESI-MS data were obtained on an Agilent 6210 ESI/TOF mass spectrometer. Optical rotation was recorded in CH_3_Cl_3_ on Jasco P-1020 polarimeter at room temperature. Infrared (IR) spectra were measured on a Jasco FT/IR-480 plus Fourier Transform infrared spectrometer using KBr pellet. Nuclear magnetic resonance (NMR) spectra were measured on Bruker AV-300 or AV-400 spectrometers. Thin-layer chromatography (TLC) analyses were carried out using pre-coated silica gel GF_254_ plates (Qingdao Marine Chemical Plant, P.R. China). Column chromatographies were performed on silica gel (200–400 mesh, Qingdao Marine Chemical Plant, P.R. China), reverse-phase C_18_ silica gel (Merck, Germany) and Sephadex LH-20 (Pharmacia Biotec AB, Sweden). The solvents used in column chromatography and high-performance liquid chromatography (HPLC) were of analytical (Shanghai, P.R. China) grade and chromatographic grade (Fisher Scientific, USA), respectively.

### Plant Material

The dried roots of *Salvia miltiorrhiza* were purchased from Qingping Herbal Medicine Market (Guangzhou, P.R. China) and identified by Prof. Guang-xiong Zhou in Jinan University. A voucher specimen (No. 20090901) was deposited in the Institute of Traditional Chinese Medicine and Natural Products, Jinan University, China.

### Extraction and Enrichment of Total Tanshinones

The dried roots of *Salvia miltiorrhiza* (10 kg) was crushed and extracted using 95% ethanol. The crude extract was suspended in distilled water and partitioned with dichloromethane to afford the dichloromethane fraction 178.5 g, which was revealed to be the total tanshinones (95%, estimated based on the online UV spectrum and the total peak areas of individual peaks) by HPLC analysis.

### Combinatorial Modification and Purification of Tanshinone Derivatives

The extract of dichloromethane fraction (10.0 g) was dissolved in ethanol (300 ml), benzaldehyde (5.96 ml, 53 mmol) and ammonium acetate (27.0 g, 0.35 mol) were added to the solution. The solution was stirred for 24 hours under 35°C. Then the solvent was removed in vacuum and the residue was washed 2 times by waters to remove the excess ammonium acetate and afford the semi-synthetic mixture (17.1 g). The semi-synthetic mixture was subjected to silica gel (200–300 mesh), eluted with cyclohexane-acetic ether (50∶1, 30∶1, 20∶1, 15∶1, 10∶1, 8∶1, 7∶1, 5∶1, 3∶1, 2∶1 and 1∶1) to give 8 fractions (Fr. 1 to Fr. 8). Compounds **1** (10.4 mg) and **2** (5.1 mg) were directly crystallized from Fr. 4 and Fr. 1, respectively. Fr. 2 was separated by Sephadex LH-20 eluted with chloroform-methanol (1∶1) and preparative HPLC eluted with acetonitrile-water (70∶30) to afford **6** (25.5 mg) and **4** (4.2 mg). **3** (7.0 mg) was obtained from Fr. 7 by preparative HPLC eluted with acetonitrile-water (75∶25). Similarly, compounds **5** (2.8 mg) and **7** (6.5 mg) were obtained from Fr. 8 by preparative HPLC with the same gradient system as **3**. Fr. 5 was chromatographed on silica gel eluted with gradient cyclohexane-acetic ether (50∶1, 30∶1, 20∶1, 15∶1, 10∶1, 5∶1 and 1∶1) to give subfractions Fr. 5a- 5f. Fr. 5c was further purified by preparative HPLC eluted with acetonitrile-water (85∶15) to afford **9** (22.9 mg) and **10** (2.2 mg). Similarly, Fr. 5d was separated by preparative HPLC with the same mobile phase as Fr. 5c to afford **8** (5.5 mg) and **11** (4.2 mg). The purity of compounds was determined to be ≥97% by HPLC analysis. A stack plot showing the HPLC chromatograms of individual compounds were shown in File S1.

Compound **1**: m.p. 288–290°C; ESI-MS *m/z*: 363.4 [M+H^+^], 361.4 [M-H^−^]; HR-ESIMS *m*/*z* [M+H]^+^ 363.1510, calculated for C_25_H_18_N_2_O requires 363.1492. UV-Vis (MeOH): 374.0, 355.8, 307.8, 265.8, 203.6 nm; IR (KBr): 3435, 2962, 2930, 1628, 1455, 1367, 1289, 775, 694 cm^−1^. ^1^H-NMR *δ*_H_10.87 (1H, d, *J*  =  8.4 Hz, H-1), 7.72 (1H, dd, *J*  =  7.6, 8.4 Hz, H-2), 7.54 (1H, d, *J*  =  7.6 Hz, H-3), 8.13 (1H, d, *J*  =  9.2 Hz, H-6), 8.37 (1H, d, *J*  =  9.2 Hz, H-7), 8.02 (1H, s, H-16), 2.66 (3H, s, H-17), 2.78 (3H, s, H-18), 8.45 (2H, d, *J*  =  7.4 Hz, H-2′,6′), 7.65 (2H, dd, *J*  =  7.4, 7.6 Hz, H-3′,5′), 7.54 (1H, t, *J*  =  7.6 Hz, H-4′), 13.04 (1H, s, N-H); ^13^C-NMR *δ*_C_ 126.7 (C-1), 126.2 (C-2), 127.0 (C-3), 133.9 (C-4), 130.4 (C-5), 121.8 (C-6), 118.9 (C-7), 115.6 (C-8), 119.2 (C-9), 130.5 (C-10), 130.3 (C-11), 126.2 (C-12), 112.8 (C-13), 148.8 (C-14), 115.4 (C-15), 142.0 (C-16), 9.7 (C-17), 19.9 (C-18), 136.5 (C-1′), 127.0 (C-2′,6′), 128.8 (C-3′,5′), 129.4 (C-4′), 149.0 (C-7′).

Compound **2**: m.p. 210–212°C; ESI-MS *m/z*: 365.5 [M+H^+^], 363.8 [M-H^−^]; HR-ESIMS *m*/*z* [M+H]^+^ 365.1685, calculated for C_25_H_20_N_2_O requires 365.1648; UV-Vis(MeOH): 370.2, 352.4, 336.8, 275.8, 202.8 nm; IR(KBr): 3215, 2878, 2828, 1605, 1482, 1452, 1348, 1024, 696 cm^−1^. *δ*_H_ 4.25 (2H, t, *J*  =  8.4 Hz, H-1), 2.36 (2H, m, H-2), 7.55 (1H, t, *J*  =  5.7 Hz, H-3), 7.50 (1H, d, *J*  =  8.4 Hz, H-6), 8.14 (1H, d, *J*  =  8.4 Hz, H-7), 7.90 (1H, s, H-16), 2.60 (3H, s, H-17), 2.15 (3H, s, H-18), 8.31 (2H, d, *J*  =  7.2 Hz, H-2′,6′), 7.58 (2H, dd, *J*  =  7.2, 7.5 Hz, H-3′,5′), 7.47 (1H, t, *J*  =  7.5 Hz, H-4′), 12.85 (1H, s, N-H). 13C-NMR *δ*_C_ 22.8 (C-1), 24.8 (C-2), 120.4 (C-3), 133.1 (C-4), 132.6 (C-5), 125.5 (C-6), 118.4 (C-7), 118.1 (C-8), 121.7 (C-9), 132.1 (C-10), 130.6 (C-11), 125.5 (C-12), 112.3 (C-13), 147.1 (C-14), 115.5 (C-15), 141.4 (C-16), 9.7 (C-17), 20.0 (C-18), 136.1 (C-1′), 126.6 (C-2′,6′), 128.7(C-3′,5′), 129.0 (C-4′), 148.8 (C-7′).

Compound **3**: m.p. 250–251°C; ESI-MS *m/z*: 365.5 [M+H^+^], 363.4 [M-H^−^]; HR-ESIMS *m*/*z* [M+H]^+^ 365.1663, calculated for C_25_H_20_N_2_O requires 365.1648; UV-Vis(MeOH): 390.6, 371.2, 306.2, 244.6, 204.2 nm; IR(KBr): 3438, 3053, 2961, 2930, 1632, 1454, 1386, 773, 689 cm^−1^. ^1^H-NMR *δ*_H_10.74 (1H, d, *J*  =  8.7 Hz, H-1), 7.68 (1H, dd, *J*  =  7.5, 8.7 Hz, H-2), 7.51 (1H, d, *J*  =  7.5 Hz, H-3), 7.96 (1H, d, *J*  =  9.0 Hz, H-6), 8.20 (1H, d, *J*  =  9.0 Hz, H-7), 4.07 (1H, m, H-15), 4.96 (1H, t, *J*  =  9.0 Hz, H-16a), 4.50 (1H, dd, *J*  =  9.0, 4.2 Hz, H-16b), 1.53 (3H, d, *J*  =  6.6 Hz H-17), 2.75 (3H, s, H-18), 8.37 (2H, d, *J*  =  7.5 Hz, H-2′,6′), 7.62 (2H, dd, *J*  =  7.5, 8.7 Hz, H-3′,5′), 7.51 (1H, t, *J*  =  8.7 Hz, H-4′), 13.07 (1H, s, N-H); ^13^C-NMR *δ*_C_ 126.7 (C-1), 126.0 (C-2), 127.3 (C-3), 133.7 (C-4), 130.4 (C-5), 120.5 (C-6), 120.5 (C-7), 114.7 (C-8), 121.6 (C-9), 130.8 (C-10), 130.1 (C-11), 126.0 (C-12), 111.7 (C-13), 148.4 (C-14), 36.3 (C-15), 78.8 (C-16), 19.8 (C-17), 19.9 (C-18), 135.6 (C-1′), 126.3 (C-2′,6′), 129.0 (C-3′,5′), 129.3 (C-4′), 152.4 (C-7′).

Compound **4**: ESI-MS *m/z*: 365.6 [M+H^+^], 363.6 [M-H^-^]; HR-ESIMS *m*/*z* [M+H]^+^ 365.1655, calculated for C_25_H_20_N_2_O requires 365.1648; UV-Vis(MeOH): 368.2, 353.0, 272.2, 224.4, 203.8 nm; IR(KBr): 3200, 2942, 2851, 1618, 1450, 1386, 693 cm^−1^. *δ*_H_ 4.09 (2H, t, *J*  =  6.0 Hz, H-1), 2.04 (2H, m, H-2), 2.61 (1H, m, H-3), 7.90 (1H, d, *J*  =  8.7 Hz, H-6), 8.10 (1H, d, *J*  =  8.7 Hz, H-7), 7.94 (1H, s, H-16), 2.61 (3H, s, H-17), 5.07 (1H, br.s, H-18a), 5.65 (1H, br.s, H-18b), 8.32 (2H, d, *J*  =  7.5 Hz, H-2′,6′), 7.59 (2H, dd, *J*  =  7.5, 7.4 Hz, H-3′,5′), 7.48 (1H, t, *J*  =  7.4 Hz, H-4′), 12.90 (1H, s, N-H); ^13^C-NMR *δ*_C_ 30.3 (C-1), 23.6 (C-2), 32.6 (C-3), 150.5 (C-4), 144.3 (C-5), 121.5 (C-6), 117.9 (C-7), 118.3 (C-8), 122.1 (C-9), 134.4 (C-10), 131.6 (C-11), 125.4 (C-12), 112.3 (C-13), 147.6 (C-14), 115.4 (C-15), 141.7 (C-16), 9.71 (C-17), 109.5 (C-18), 136.5 (C-1′), 126.7 (C-2′,6′), 128.7 (C-3′,5′), 129.0 (C-4′), 148.6 (C-7′).

Compound **5**: ESI-MS *m/z*: 399.5 [M+H^+^], 397.5 [M-H^-^]; UV-Vis(MeOH): 368.2, 353.0, 272.2, 224.4, 203.8 nm; IR(KBr): 3395, 2925, 1624, 1385, 1026, 694 cm^−1^. *δ*_H_ 2.12 (2H, t, *J*  =  6.6 Hz, H-1), 1.95 (2H, m, H-2), 3.57 (1H, m, H-3a), 4.14 (1H, t, *J*  =  5.1 Hz, H-3b), 7.83 (1H, d, *J*  =  8.4 Hz, H-6), 8.12 (1H, d, *J*  =  8.4 Hz, H-7), 7.91 (1H, s, H-16), 2.61 (3H, s, H-17), 4.01 (1H, d, *J*  =  6.6 Hz H-18a), 3.77 (1H, m, H-18b), 8.32 (2H, d, *J*  =  7.8 Hz, H-2′,6′), 7.58 (2H, dd, *J*  =  7.2, 7.8 Hz, H-3′,5′), 7.47 (1H, t, *J*  =  7.2 Hz, H-4′), 12.83 (1H, s, N-H); ^13^C-NMR *δ*_C_ 29.9 (C-1), 20.4 (C-2), 38.5 (C-3), 69.2 (C-4), 141.8 (C-5), 123.9 (C-6), 117.6 (C-7), 117.6 (C-8), 122.0 (C-9), 132.5 (C-10), 130.7 (C-11), 125.2 (C-12), 112.0 (C-13), 147.2 (C-14), 115.3 (C-15), 141.3 (C-16), 9.5 (C-17), 71.3 (C-18), 136.4 (C-1′), 126.6 (C-2′,6′), 128.6 (C-3′,5′), 128.8 (C-4′), 148.7 (C-7′).

Compound **6**: m.p.128–130°C; ESI-MS *m/z*: 381.5 [M+H^+^], 379.5 [M-H^−^]; HR-ESIMS *m*/*z* [M+H]^+^ 381.1981, calculated for C_26_H_24_N_2_O requires 381.1961; UV-Vis(MeOH): 352.4, 383.2, 260.6, 204.2 nm; IR(KBr): 3500, 2924, 2854, 1608, 1455, 1386, 1260, 691 cm^−1^. *δ*_H_ 3.96 (2H, t, *J*  =  6.6 Hz, H-1), 1.95 (2H, m, H-2), 1.75 (2H, m, H-3), 7.61 (1H, d, *J*  =  8.8 Hz, H-6), 8.08 (1H, d, *J*  =  8.8 Hz, H-7), 7.88 (1H, d, *J*  =  1.0, H-16), 2.66 (3H, s, H-17), 1.37 (3H, s, H-18), 1.37 (3H, s, H-19), 8.31 (2H, d, *J*  =  7.4 Hz, H-2′,6′), 7.57 (2H, dd, *J*  =  7.4, 7.6 Hz, H-3′,5′), 7.47 (1H, t, *J*  =  7.6 Hz, H-4′), 12.80 (1H, s, N-H); ^13^C-NMR *δ*_C_ 30.8 (C-1), 19.6 (C-2), 38.5 (C-3), 34.3 (C-4), 142.7 (C-5), 123.8 (C-6), 117.8 (C-7), 117.1 (C-8), 122.6 (C-9), 132.8 (C-10), 130.8 (C-11), 125.4 (C-12), 111.8 (C-13), 147.1 (C-14), 115.3 (C-15), 141.2 (C-16), 9.7 (C-17), 32.0 (C-18), 32.0 (C-19), 136.4 (C-1′), 126.6 (C-2′,6′), 128.7 (C-3′,5′), 128.9 (C-4′), 148.8 (C-7′).

Compound **7**: ESI-MS *m/z*: 397.5 [M+H^+^], 395.6 [M-H^−^]; HR-ESIMS *m*/*z* [M+H]^+^ 397.1952, calculated for C_26_H_24_N_2_O_2_ requires 397.1911; UV-Vis(MeOH): 353.0, 337.4, 261.0, 203.8 nm; IR(KBr): 3361, 2929, 2866, 1608, 1452, 1397, 1028, 694 cm^−1^. *δ*_H_ 3.97 (2H, t, *J*  =  5.4 Hz, H-1), 2.00 (2H, m, H-2), 1.55 (2H, m, H-3), 7.60 (1H, d, *J*  =  8.4 Hz, H-6), 8.07 (1H, d, *J*  =  8.4 Hz, H-7), 7.89 (1H, s, H-16), 2.61 (3H, s, H-17), 1.31 (3H, s, H-18), 3.54 (1H, br.s, H-19a), 3.62 (1H, br.s, H-19b), 8.32 (2H, d, *J*  =  7.4 Hz, H-2′,6′), 7.58 (2H, dd, *J*  =  7.4, 7.6 Hz, H-3′,5′), 7.47 (1H, t, *J*  =  7.6 Hz, H-4′), 12.80 (1H, s, N-H); ^13^C-NMR *δ*_C_ 30.7 (C-1), 19.2 (C-2), 32.3 (C-3), 34.5 (C-4), 139.9 (C-5), 124.1 (C-6), 117.4 (C-7), 117.1 (C-8), 122.6 (C-9), 134.2 (C-10), 130.8 (C-11), 125.4 (C-12), 111.8 (C-13), 147.1 (C-14), 115.2 (C-15), 141.1 (C-16), 9.5 (C-17), 26.8 (C-18), 69.7 (C-19), 136.3 (C-1′), 126.6 (C-2′,6′), 128.6 (C-3′,5′), 128.8 (C-4′), 148.8 (C-7′).

Compound **8**: ESI-MS *m/z*: 425.4 [M+H^+^], 423.4 [M-H^−^]; HR-ESIMS *m*/*z* [M+H]^+^ 425.1875, calculated for C_27_H_24_N_2_O_3_ requires 425.1860; UV-Vis(MeOH): 353.2, 337.4, 262.4, 203.8 nm; IR(KBr): 3392, 2946, 2860, 1726, 1610, 1451, 1384, 1256, 694 cm^−1^. *δ*_H_ 4.02 (2H, t, *J*  =  5.4 Hz, H-1), 2.25 (2H, m, H-2), 1.88 (2H, m, H-3), 7.34 (1H, d, *J*  =  8.4 Hz, H-6), 8.09 (1H, d, *J*  =  8.4 Hz, H-7), 7.93 (1H, s, H-16), 2.61 (3H, s, H-17), 1.61 (3H, s, H-18), 3.60 (3H, s, COCH_3_), 8.33 (2H, d, *J*  =  7.4 Hz, H-2′,6′), 7.59 (2H, dd, *J*  =  7.4, 7.5 Hz, H-3′,5′), 7.48 (1H, t, *J*  =  7.5 Hz, H-4′), 12.91 (1H, s, N-H); ^13^C-NMR *δ*_C_ 30.1 (C-1), 19.6 (C-2), 34.2 (C-3), 46.9 (C-4), 148.6 (C-5), 124.7 (C-6), 118.0 (C-7), 117.6 (C-8), 122.5 (C-9), 133.5 (C-10), 130.7 (C-11), 125.6 (C-12), 112.3 (C-13), 145.8 (C-14), 115.3 (C-15), 141.7 (C-16), 9.9 (C-17), 27.6 (C-18), 177.5 (C-19), 52.5 (COCH_3_), 136.3 (C-1′), 126.7 (C-2′,6′), 128.7 (C-3′,5′), 129.0 (C-4′), 148.6 (C-7′).

Compound **9**: ESI-MS *m/z*: 383.4 [M+H^+^], 381.5 [M-H^−^]; HR-ESIMS *m*/*z* [M+H]^+^ 383.2111, calculated for C_26_H_26_N_2_O requires 383.2118; UV-Vis(MeOH): 367.4, 354.4, 259.0, 236.6, 205.2 nm; IR(KBr): 3408, 3061, 2956, 2924, 1604, 1454, 1406, 1382, 696 cm^−1^. *δ*_H_ 3.91 (2H, t, *J*  =  6.6 Hz, H-1), 1.93 (2H, m, H-2), 1.73 (2H, m, H-3), 7.46 (1H, d, *J*  =  8.8 Hz, H-6), 7.76 (1H, d, *J*  =  8.8 Hz, H-7), 3.97 (1H, m, H-15), 4.87 (1H, t, *J*  =  8.8 Hz, H-16a), 4.42 (1H, dd, *J*  =  4.4, 8.8 Hz, H-16b), 1.46 (3H, d, *J*  =  6.8 Hz, H-17), 1.33 (3H, s, H-18), 1.33 (3H, s, H-19), 8.25 (2H, d, *J*  =  7.4 Hz, H-2′,6′), 7.55 (2H, dd, *J*  =  7.4, 7.4 Hz, H-3′,5′), 7.42 (1H, t, *J*  =  7.4 Hz, H-4′), 12.83 (1H, s, N-H); ^13^C-NMR *δ*_C_ 30.6 (C-1), 19.6 (C-2), 38.5 (C-3), 34.2 (C-4), 142.8 (C-5), 122.6 (C-6), 119.4 (C-7), 116.3 (C-8), 129.1 (C-9), 132.0 (C-10), 130.9 (C-11), 124.8 (C-12), 110.0 (C-13), 146.4 (C-14), 36.2 (C-15), 78.7 (C-16), 19.8 (C-17), 31.8 (C-18), 31.8 (C-19), 135.3 (C-1′), 125.8 (C-2′,6′), 128.7 (C-3′,5′), 128.4 (C-4′), 152.0 (C-7′).

Compound **10**: ESI-MS *m/z*: 357.4 [M+H^+^], 355.4 [M-H^−^], 379.4 [M+Na^+^], 735.1 [2M+Na^+^]; UV-Vis (MeOH): 368.2, 353.0, 272.2, 224.4, 203.8 nm; IR(KBr): 3213, 2930, 2865, 1644, 1563, 1472, 1450, 1384, 1278, 702 cm^−1^. HR-ESIMS *m*/*z* [M+H]^+^ 357.1597, calculated for C_23_H_20_N_2_O_2_ requires 357.1598. *δ*_H_ 3.54 (2H, t, *J*  =  6.3 Hz, H-1), 1.84 (2H, m, H-2), 1.67 (2H, m, H-3), 7.52 (1H, d, *J*  =  8.4 Hz, H-6), 7.76 (1H, d, *J*  =  8.4 Hz, H-7), 1.31 (3H, s, H-18), 1.31 (3H, s, H-19), 8.24 (2H, d, *J*  =  6.6 Hz, H-2′,6′), 7.56 (2H, dd, *J*  =  6.6, 7.2 Hz, H-3′,5′), 7.54 (1H, t, *J*  =  7.2 Hz, H-4′), 14.01 (1H, s, N-H); ^13^C-NMR *δ*_C_ 29.8 (C-1), 19.1 (C-2), 37.6 (C-3), 34.7 (C-4), 154.4 (C-5), 127.1 (C-6), 127.4 (C-7), 131.6 (C-8), 137.1 (C-9), 135.1 (C-10), 128.8 (C-11), 168.1 (C-12), 182.0 (C-13), 180.4 (C-14), 31.5 (C-18), 31.5 (C-19), 136.5 (C-1′), 126.8 (C-2′,6′), 129.0 (C-3′,5′), 129.0(C-4′), 145.3 (C-7′).

Compound **11**: ESI-MS *m/z*: 384.3 [M+H^+^], 382.4 [M-H^−^], 789.2 [2M+Na^+^]; UV-Vis (MeOH): 368.2, 353.0, 272.2, 224.4, 203.8 nm; IR(KBr): 3358, 2922, 2851, 1466, 1384, 1289, 775, 693 cm^−1^. HR-ESIMS *m*/*z* [M+H]^+^ 384.1588, calculated for C_25_H_21_NO_3_ requires 384.1594. ^1^H-NMR *δ*_H_ 10.25 (1H, d, *J*  =  8.4 Hz, H-1), 7.72 (1H, dd, *J*  =  5.4, 6.3 Hz, H-2), 7.56 (1H, d, *J*  =  6.9 Hz, H-3), 8.04 (1H, d, *J*  =  9.3 Hz, H-6), 8.38 (1H, d, *J*  =  9.3 Hz, H-7), 3.89 (1H, m, H-15), 4.01 (1H, dd, *J*  =  14.6, 6.6 Hz, H-16a), 4.04 (1H, dd, *J*  =  14.6, 7.2 Hz, H-16b), 1.51 (3H, d, *J*  =  3.9 Hz, H-17), 2.77 (3H, s, H-18), 8.33 (2H, d, *J*  =  7.6 Hz, H-2′,6′), 7.68 (2H, t, *J*  =  7.6 Hz, H-3′,5′), 7.66 (1H, t, *J*  =  7.6 Hz, H-4′); ^13^C-NMR *δ*_C_ 126.3 (C-1), 126.0 (C-2), 127.9 (C-3), 133.9 (C-4), 130.8 (C-5), 121.6 (C-6), 121.3 (C-7), 113.4 (C-8), 121.8 (C-9), 129.4 (C-10), 129.2 (C-11), 149.2 (C-12), 113.4 (C-13), 159.5 (C-14), 33.8 (C-15), 64.9 (C-16), 16.1 (C-17), 19.7 (C-18), 126.9 (C-1′), 126.8 (C-2′,6′), 129.4 (C-3′,5′), 131.2 (C-4′), 149.4 (C-7′).

### Ethics Statement

All animal experiments were conducted in accord with the ethical guidelines of the Animal Ethics Committee, Institute of Chinese Medical Sciences, University of Macau and the protocol was approved by Animal Ethics Committee, Institute of Chinese Medical Sciences, University of Macau prior to the initiation of the experiments.

### Chemicals and Reagents in Zebrafish and Cell Culture assays

3-(4,5-Dimethylthiazol-2-yl)-2,5-diphenyltetrazolium bromide (MTT) and dimethyl sulfoxide (DMSO) were acquired from Sigma (USA). VEGFR tyrosine kinase inhibitor II (VRI), FGFR inhibitor (SU5402), EGFR inhibitor (PD 153035), non-selective eNOS inhibitor (L-NAME), Src inhibitor (PP2), P38 MAP kinase Inhibitor III (P38i), Raf kinase inhibitor IV (Rafi), MEK1/2 inhibitor (MEK1/2i), ERK1/2 inhibitor (ERK1/2i), PI3K inhibitor (LY 294002), HIF-1 inhibitor (HIFi) and three subclasses of Akt inhibitor (Akti) were purchased from Calbiochem (Merck, Germany), and each compound was dissolved in their recommended solvent, as per manufacturers. Tan IIA, CPT and Tan I were purchased from Jiangsu Yongjian Co., Ltd (Taizhou, P.R. China). Tanshinone derivatives and three tanshiones were dissolved in DMSO at a concentration of 50 mM. Purified water was prepared using a Milli-Q purification system from Millipore (Millipore, USA).

### Maintenance of Zebrafish

The zebrafish strains, including *Tg(fli-1a:EGFP)y1* and Wide-type, were maintained as previously described in our previous paper [31].

### Embryo Collection and Drug Treatment

Zebrafish embryos were generated by natural pair-wise mating (3–12 months old) and were raised at 28°C in embryo medium. Healthy, hatched zebrafish were selected at 21 hour post-fertilization (hpf) and distributed into a 12-well microplate (15 embryos per well for fluorescence observation) or 6-well microplate (30 embryos per well for RNA extract). The embryos which received embryo medium with 0.1% DMSO (v/v) only served as vehicle controls. The medium in all treatment groups was refreshed every 24 h and all groups were incubated at 28 °C.

- *Restorative treatment plan in VRI-treated zebrafish*

Embryos at 21 hpf were pre-treated with 500 ng/ml VRI for 3 h. The VRI was then washed out and replaced with either 0.1% DMSO (v/v) embryo medium or three tanshinones and eleven derivatives for another 24 h and 48 h.

*b. Specific inhibitors assay in VRI-treated zebrafish*

Embryos at 21 hpf were pretreated with 500 ng/ml VRI for 3 h. The VRI was then washed out and replaced with either 0.1% DMSO (v/v) embryo medium or only compound **10** (0.3 µM) and **10** (0.3 µM) plus co-treatment with inhibitors for 24 h.

### Morphological Observation and Assessment of Vascular Changes in Zebrafish

At 48 hpf or 72 hpf, zebrafish embryos were removed from microplates and observed for viability and gross morphological changes under a fluorescence microscope (Olympus IX81 Motorized Inverted Microscope, Japan) equipped with a digital camera (DP controller, Soft Imaging System, Olympus). Images were analyzed with Axiovision 4.2 and Adobe Photoshop 7.0. In the control group, intersegmental vessels (ISVs) showing sprouting and elongating from dorsal aorta (DA) or posterior cardinal vein (PCV) to dorsal longitudinal anastomotic vessels (DLAVs) were defined as intact. And in the VRI-only treatment group or some drug treatment groups, some of the ISVs observed sprouting from DA or PCV, but did not form complete ISVs, were defined as defective. The number of intact and defective ISVs in each zebrafish embryo was counted.

### Total RNA Extraction, Reverse Transcription and Real-time qPCR

The wild-type zebrafish embryos were treated with the protocols described above. At 48 hpf, total RNA was extracted from 30 embryos in each treatment group, using the RNeasy Mini Kit (Qiagen, USA) in accordance with the manufacturer's instructions. RNA was reverse transcribed into single-strand cDNA using III First-Strand Synthesis System for RT-PCR (Invitrogen, USA), followed by real-time PCR using the Universal PCR Master Mix, and 250 nM custom primers for zebrafish *kdrl (flk-1A), kdr (flk-1B)* and *flt1* (Applied Biosystems, USA) in the ABI 7500 Real-Time PCR System (Applied Biosystems, USA). The expression of *kdrl, kdr* and *flt-1* was normalized to the amount of *bactin1*, using the relative quantification method described by the manufacturer.

The zebrafish *bactin1* primers were 5′-CAAGATTCCATACCCAGGAAGGA -3′ (F) and 5′-CAAGATTCCATACCCAGGAAGGA-3′ (R). The zebrafish *kdrl* primers were 5′-GACCATAAAACAAGTGAGGCAGAAG-3′ (F) and 5′- CTCCT

GGTTTGACAGAGCGATA-3′ (R). The zebrafish *kdr* primers were 5′-CAAGTA

ACTCGTTTTCTCAACCTAAGC-3′ (F) and 5′-GGTCTGCTACACAACGCATT

ATAAC- 3′ (R). The zebrafish *flt-1* primers were 5′-AACTCACAGACCAGTGA

ACAAGATC- 3′ (F) and 5′-GCCCTGTAACGTGTGCACTAAA-3′ (R).

### Cancer Cell Lines Culture and MTT Assay

Cancer cells were maintained in suitable medium (A549 and MCF-7 cell lines in DMEM culture medium; MDA-MB-231 in RPMI 1640 culture medium) (ATCC,USA), supplemented with 10% heat-inactivated fetal bovine serum (FBS) (Invitrogen,USA), and 1% antibiotics (100 IU/ml penicillin and 100 µg/ml streptomycin). Cells were grown in 75 cm^2^ tissue culture flasks in a humidified atmosphere containing 5% CO_2_, at 37°C.

The cancer cells were plated into a 96-well plate at a concentration of 0.8–1×10^5^ cells/well overnight to allow for cell attachment. The working solutions of compound **10** and tanshinones were dispensed appropriately into the partitioned 96-well plates then incubated for another 48 h. The cells were also incubated with DMSO (0.1%) which served as the vehicle control. Then the medium was discarded and cells were incubated for 4 h at 37°C in MTT solution (final concentration 0.5 mg/ml). The solution was then replaced by 100 µl DMSO to dissolve the violet formazan crystals in intact cells. The absorbance was measured at a wavelength of 570 nm by a SpectraMaxR M5 Multi-Mode Microplate Readers (Molecular Devices, USA). Cell viability was expressed as a percentage of the vehicle control.

### Statistical analysis

Each experiment was done in triplicate at least. The mean and standard deviations were compared using Students' t-test with the following statistical criteria: p<0.05, significant; p<0.01, highly significant; p<0.001 extremely significant. All relevant data are presented as mean±SEM.

## Results

### Synthesis of New Tanshinone Derivatives

**Figure 1:**
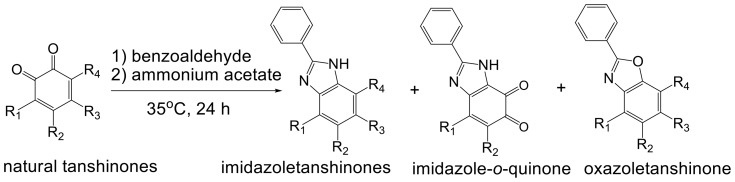
The one-pot modification of tanshinone mixture from *Salvia miltiorrhiza*.

**Figure 2:**
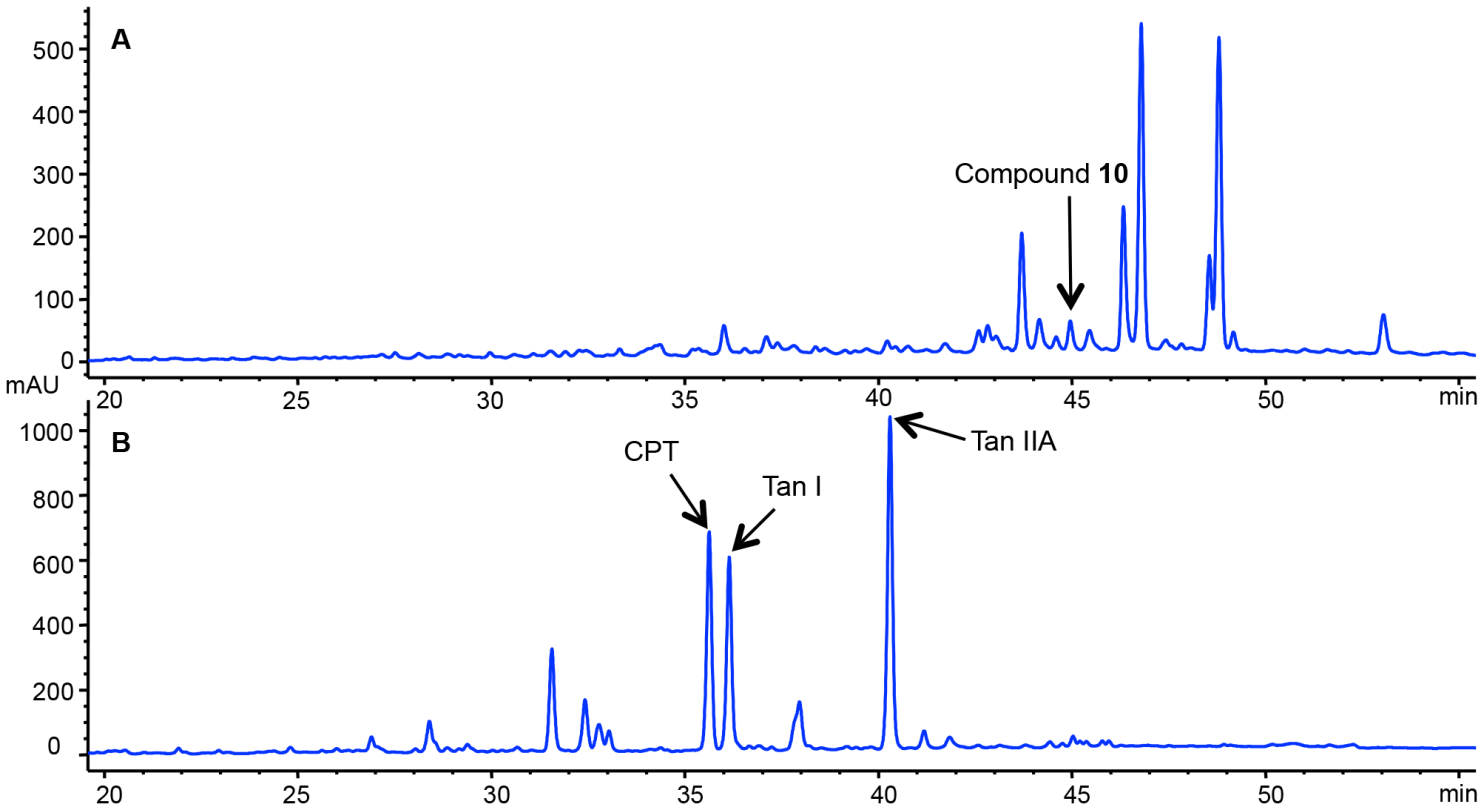
HPLC traces of the combinatorial modified tanshinones extract highlighting the minor but active compound 10 (A), and total tanshinones of *Salvia miltiorrhiza* highlighting the three major natural tanshinones (B).

**Figure 3:**
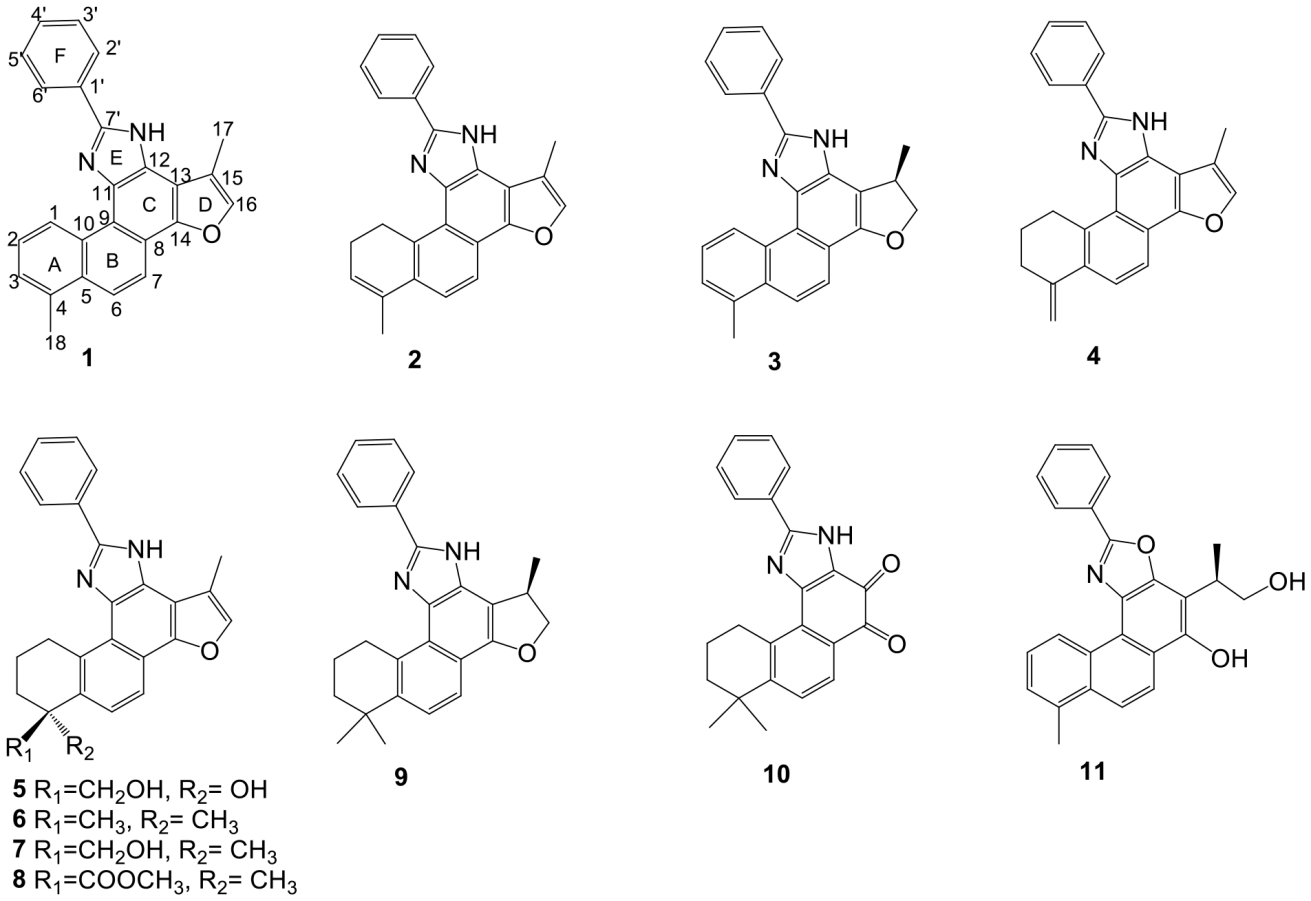
Structural formulae of eleven tanshinone derivatives.

In our study, total tanshinones (95%) fraction was isolated and enriched from an ethanol extract of *Salvia miltiorrhiza* and identified by HPLC analysis. One-pot synthesis was then carried out by reacting the total tanshinones fraction with benzaldehyde and ammonium acetate under 35°C to obtain a semi-synthetic mixture (Fig. 1). A comparison of the HPLC chemical profiles of the semi-synthetic mixture with that of the unreacted total tanshinones revealed a marked change in the chemical composition (Fig. 2). The semi-synthetic mixture showed a group of peaks with retention times at 42–50 min, whereas the most prominent peaks of the parental total tanshinones were concentrated at 30–42 min. Further subsequent isolation of the semi-synthetic mixture resulted in eleven new derivatives named as compounds **1–** **11** (Fig. 3), which could be classified into three groups according to their structural scaffolds.

Compounds **1–** **5** were classified into a group which was composed of the 2-phenylimidazole and C18 tanshinone moieties. Compound **1** possessed a fully conjugated ring system with methyl groups located at C-4 and C-15. Compounds **2** and **3** were similar to those of **1** with the absence of Δ^1,2^ and Δ^15,16^ double bonds, respectively. Compound **4** was also an analog of **1**, however, the three aromatic methines in ring A were replaced by three alicyclic methylenes and the methyl group at C-4 was replaced by an exo-methylene. The structure of **5** was similar to **4** except that the exo-methylene moiety in **4** was replaced by a hydroxyl and a hydroxymethylene groups.

Compounds **6–** **9** were classified into another group which was composed of the 2-phenylimidazole and C-19 tanshinone moieties. Compound **6** was a typical example of this group. The skeleton of **6** was the same as that of **5**, but the substituents at C-4 were changed to two methyl groups. Compounds **7** and **8** were analogs of **6** with one of the methyl group at C-4 changed to a hydroxymethylene and a methyl carboxylate group, respectively. Compound **9** was also similar to **6**, however, the double bond Δ^15,16^ in the furan ring was absent.

The last miscellaneous group included compounds **10** and **11**. In **10**, no signals for the furan ring were observed in the ^1^H and ^13^C NMR data. In contrast, two conjugated carbonyl groups were found in C-13 and C-14. In **11**, there was only one nitrogen atom in the molecule as revealed by the HRESI-MS (*m/z* 384.1588 [M+H]^+^), suggesting that the phenylimidazole might be replaced by a phenyloxazole ring, which was further confirmed by the absence of a proton signal for the NH group.

**Figure 4:**
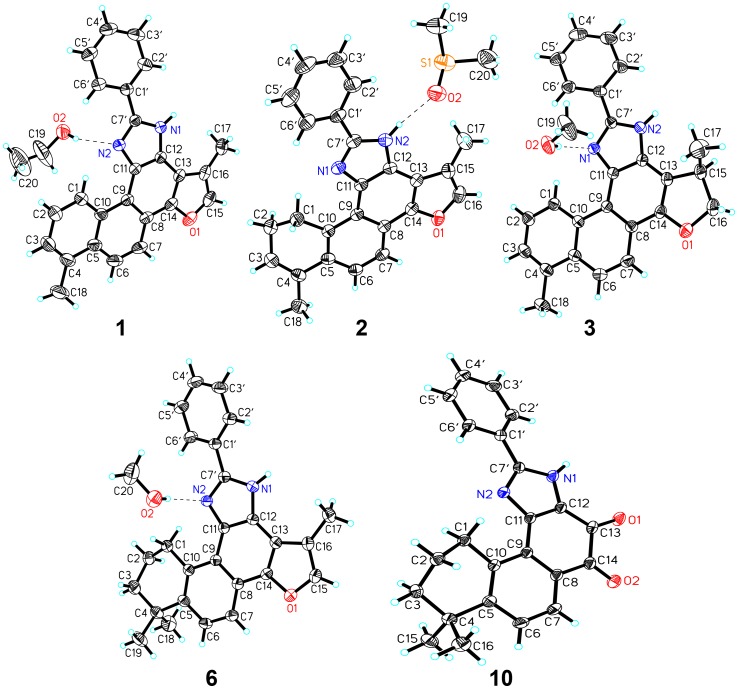
X-ray structures of compound*s* 1, 2, 3, 6 and 10.

All those compounds were identified by extensive spectroscopic methods (For detailed structural elucidation, see Section 2 of File S1; for the high resolution ESIMS data, ^1^H, ^13^C and DEPT-NMR spectra, see Section 3 of File S1), and the structures of **1**, **2**, **3**, **6** and **10** were further confirmed by single crystal X-ray analysis (Fig. 4) (Crystal data and CCDC numbers for **1**, **2**, **3**, **6** and **10**, see Table S1 in File S1).

Compounds **1–** **11** were produced by a reaction between the total tanshinone fraction, benzaldehyde and ammonium acetate through a modified Radziszewski reaction [32]. In brief, ammonia released from ammonium acetate reacted with the α-dicarbonyl compound to form α-diimine, which then condensed with benzaldehyde to form the imidazole derivatives (**1–** **10**) and one oxazole derivative **(11)**, through two different pathways. Based on this hypothetical reaction mechanism, we deduced that **1–** **11** are derived from different natural parental tanshinones including Tan I (**1a**) [33], 1,2-dihydrotanshinone I (**2a**) [33], 15,16-dihydrotanshinone I (**3a**) [33], methylenetanshinquinone (**4a**) [33], tanshindiol A (**5a**) [34], Tan IIA (**6a**) [33], tanshinone IIB (**7a**) [33], methyl tanshinonate (**8a**) [8], CPT (**9a**) [33], neo-cryptotanshinone II (**10a**) [35], and neocryptotanshinone (**11a**) [33], respectively. (The structures of these natural parental tanshinones were shown in Section 5 of File S1). It is interesting to note that with the exception of Tan IIA (**6a**), CPT (**9a**) and Tan I (**1a**) which are generally regarded as major representative components, most of the remaining tanshinones are present in low abundance in *Salvia miltiorrhiza*. Based on the natural parental tanshinones, these new derivatives could be accorded the trivial names phenylimidazoletanshinone I (**1**), 1,2-dihydrophenylimidazoletanshinone I (**2**) [33], 15,16-dihydrophenylimidazoletanshinone I (**3**), methylenephenylimidazoletanshinquinone (**4**) [33], phenylimidazoletanshindiol A (**5**), phenylimidazoletanshinone IIA (**6**) [33], phenylimidazoletanshinone IIB (**7**), methyl phenylimidazoletanshinonate (**8**) [8], phenylimidazolecryptotanshinone (**9**), phenylimidazoleneocryptotanshinone II (**10**), and phenyloxazoleneocryptotanshinone (**11**).

It should be noted that there is a chiral atom at C-16 for **3**, **9** and **11**, and at C-4 for **5**, **7** and **8**. The absolute configurations of these natural tanshinones were well established [33]–[35] (For the known configurations, see Section 6 of File S1), and the chemical reaction took place at the diketone moiety. Thus the tanshinone derivatives would be expected to share the same absolute configurations as the corresponding parental tanshinones (16*R* for **3a**, **9a** and **11a**; 4*S* for **7a** and **8a**; 4*R* for **5a**). Furthermore, final refinement of the CuKα X-ray diffraction data of **3** resulted in a small Flack parameter 0.1, confirming the absolute configuration of this compound.

### Determination of Angiogenesis Activity in Zebrafish

**Figure 5:**
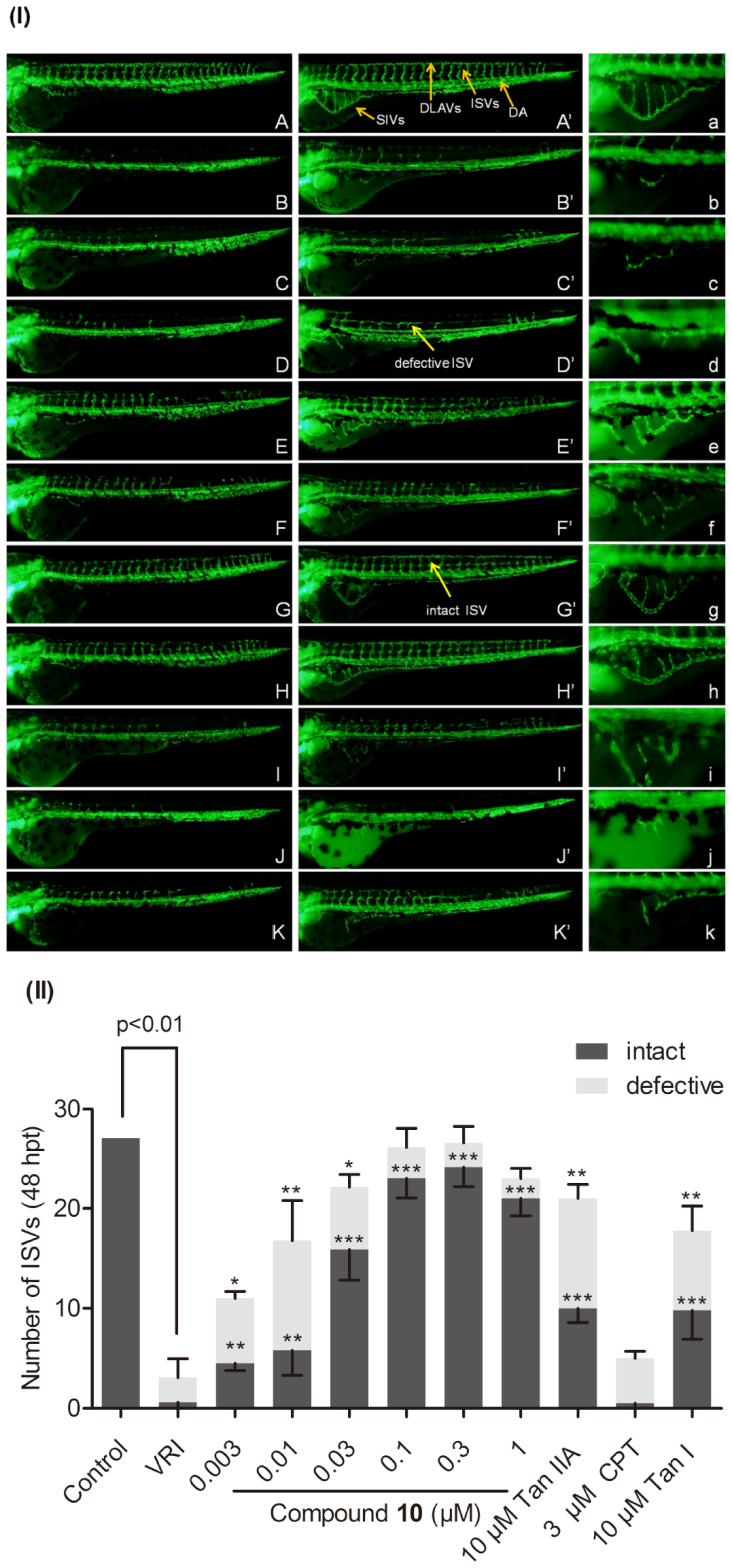
The effects of compound 10 on VRI-induced blood vessels loss in *Tg(fli-1a:EGFP)y1* zebrafish compared to Tan IIA, CPT and Tan I. (I) (A-A’) Control group: 24 hpf zebrafish embryos were treated with 0.1% DMSO (v/v) for 24 h and 48 h. 21 hpf embryos were treated with VRI (500 ng/ml) for 3 h. After that, the VRI was washed out and replaced with 0.1% DMSO (v/v) embryo medium (B-B’) or **10** [0.003 µM(C-C’), 0.01 µM(D-D’), 0.03 µM (E-E’), 0.1 µM (F-F’), 0.3 µM (G-G’),1 µM(H-H’)], 10 µM Tan IIA (I-I’), 3 µM CPT (J-J’) and 10 µM Tan I (K-K’)for 24 h and 48 h respectively. (a–k) Enlarged SIVs region (x 2.5) of (A’–K’) respectively. Yellow arrows indicated that the ISVs (intact and defective), SIVs, DLAVs, and DA. (II) Quantitative analysis of compounds on VRI-treated zebrafish. Number of defective and intact ISVs in each embryo was quantified by counting a minimum of 15 embryos per group at 48 hpt. Data are plotted as mean±SEM, (n≧3). *p<0.05, **p<0.01 and ***p<0.001 versus the VRI-only treatment group.

The angiogenesis effects of compounds **1–** **11** were determined in a chemical-induced blood vessels loss zebrafish model [30]. The major natural tanshinones including Tan IIA, CPT and Tan I were used as reference controls in the angiogenesis assay. VRI, a pyridinylanthranilamide compound that displays anti-angiogenic properties, strongly inhibits the kinase activities of both vascular endothelial growth factor receptor 1 and 2 (VEGFR1 and VEGFR2) [30]. The 21 hpf zebrafish embryos pre-treated with 500 ng/ml of VRI for 3 h were then washed out and placed in embryo medium and incubated for another 48 h. Treatment of the zebrafish with VRI induced obviously blood vessels loss in ISVs, DLAVs and the subintestinal vessels (SIVs) (Fig. 5 B-B’-b). After incubating the VRI pre-treated embryos with **1–** **11** and three major tanshinone controls for another 24 h and 48 h, the VRI-induced blood vessels loss in ISVs, DLAVs and SIVs regions were restored to different degrees. Of **1–** **11**, compound **10** (EC_50_ =  0.026 µM) and **3** (EC_50_ =  4.03 µM) exhibited significantly potency and efficacy in the recovery of blood vessels loss in VRI-treated zebrafish (see Table S2 in File S1). However, there was no significantly observable change in blood vessels following treatment with other nine derivatives at their maximum non-toxic concentrations (Table 1). Furthermore, **10** was shown to be the most potent and effective derivative and demonstrated a stronger vascular effect than Tan IIA, CPT and Tan I on the recovery of VRI-induced blood vessels loss in zebrafish (Fig. 5). Quantitative analysis of the average number of ISVs both intact and defective ISVs, confirmed the significant dose-dependent restorative effect of **10** (Fig. 5II).

**Table 1:** Screening of angiogenesis effects for tanshinone derivatives in VRI-induced blood vessels loss zebrafish embryos.

Compounds (µM)	0.1	0.3	1	3	10	30	100
Tan IIA	○	○	+	++	++	+	Dead^a^
CPT	○	○	○	○	○	Dead^a^	Dead^a^
Tan I	○	+	+	+	○	Dead^a^	Dead^a^
**1**	○	○	○	+	+	++	+
**2**	○	○	○	○	○	○	○
**3**	○	○	+	++	+++	++++	Toxic, Nd^b^
**4**	○	○	○	○	○	○	○
**5**	○	○	○	○	○	○	○
**6**	○	○	○	○	○	+	+
**7**	○	○	○	○	+	++	Toxic, Nd^b^
**8**	○	○	○	○	○	○	○
**9**	○	○	○	○	○	+	Toxic, Nd^b^
**10**	++++	+++++	+++++	+++++	++++	Toxic,Nd^b^	Dead^a^
**11**	○	○	○	○	○	+	Dead^a^

The semi-quantitative scale for restorative blood injury rates of compounds 1–11 and three tanshinones: ○, inactive; +++++, > 80% restorative rate; ++++, 60–80%; +++, 40–60%; ++,20%–40%; +< 20% restoration for ISVs as compared to the vehicle treated zebrafish embryos. ^a^Zebrafish embryos treated with the indicated concentration of compound were dead. ^b^Zebrafish embryos treated with the indicated concentration of compound were toxic and the effect was not determined.

### Determination of mRNA Expression by Real-time qPCR

**Figure 6:**
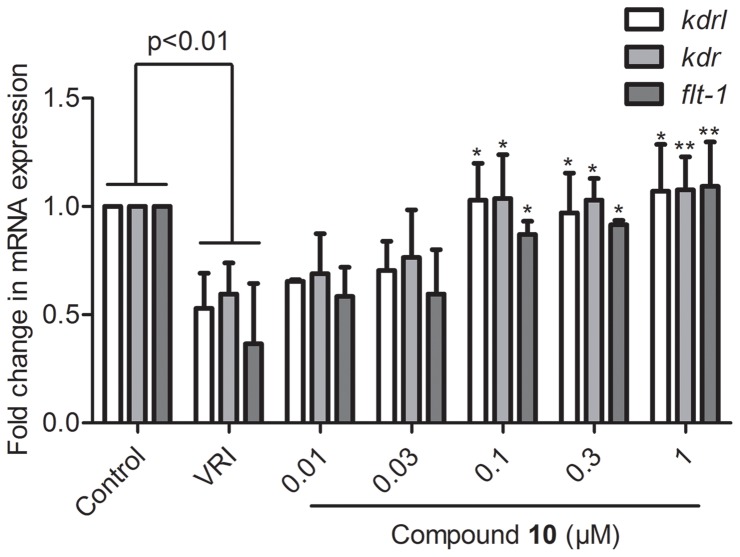
Compound 10 reversed the VRI induced down regulation of *kdrl*, *kdr* and *flt-1* mRNA expression. Zebrafish embryos (Wide-type) at 21 hpf were pre-treated with VRI for 3 h and then washed out followed by post-treatment with **10** for 24 h. Then the total RNA was extracted and reverse transcribed into cDNA for real-time qPCR. Data are expressed as the mean ± SEM, (n≧3). *p<0.05 and **p<0.01 versus VRI-only treatment group.

In order to confirm the vascular phenotypic effects observed in Fig. 5, the significant pro-angiogenesis activity of compound **10** in VRI-treated zebrafish was validated and confirmed by real-time qPCR analysis of selected key angiogenesis gene markers. Embryos were pre-treated with VRI (500 ng/ml) for 3 h, and then treated with **10** (0.01, 0.03, 0.1, 0.3 and 1 µM) for 24 h. Total RNA from different treatment groups of embryos was isolated and reverse transcribed to cDNA. The relative mRNA expressions of *flt-1* encoding the VEGFR1 as well as *kdrl* and *kdr* encoding VEGFR2 were determined using real-time qPCR. The gene expression results clearly showed that increasing dosages of **10** exhibited a significant concentration-dependent recovery of down-regulation of *kdrl*, *kdr* and *flt-1* mRNA expression in VRI-treated zebrfish. Compared to the VRI-only treatment group, the relative change in gene expression level in 1 µM **10** treatment group was 2.01-fold for *kdrl* (p<0.05), 1.80-fold for *kdr* (p<0.01) and 2.98-fold for *flt1* (p<0.01) (Fig. 6). Thus, these results suggested that **10** reversed the VRI-induced down-regulation of the expression of three key VEGF receptors in zebrafish.

### Effect of Inhibitors of FGF and EGF Signaling Pathways on Angiogenesis Effect of Compound 10 in VRI-treated Zebrafish

**Figure 7:**
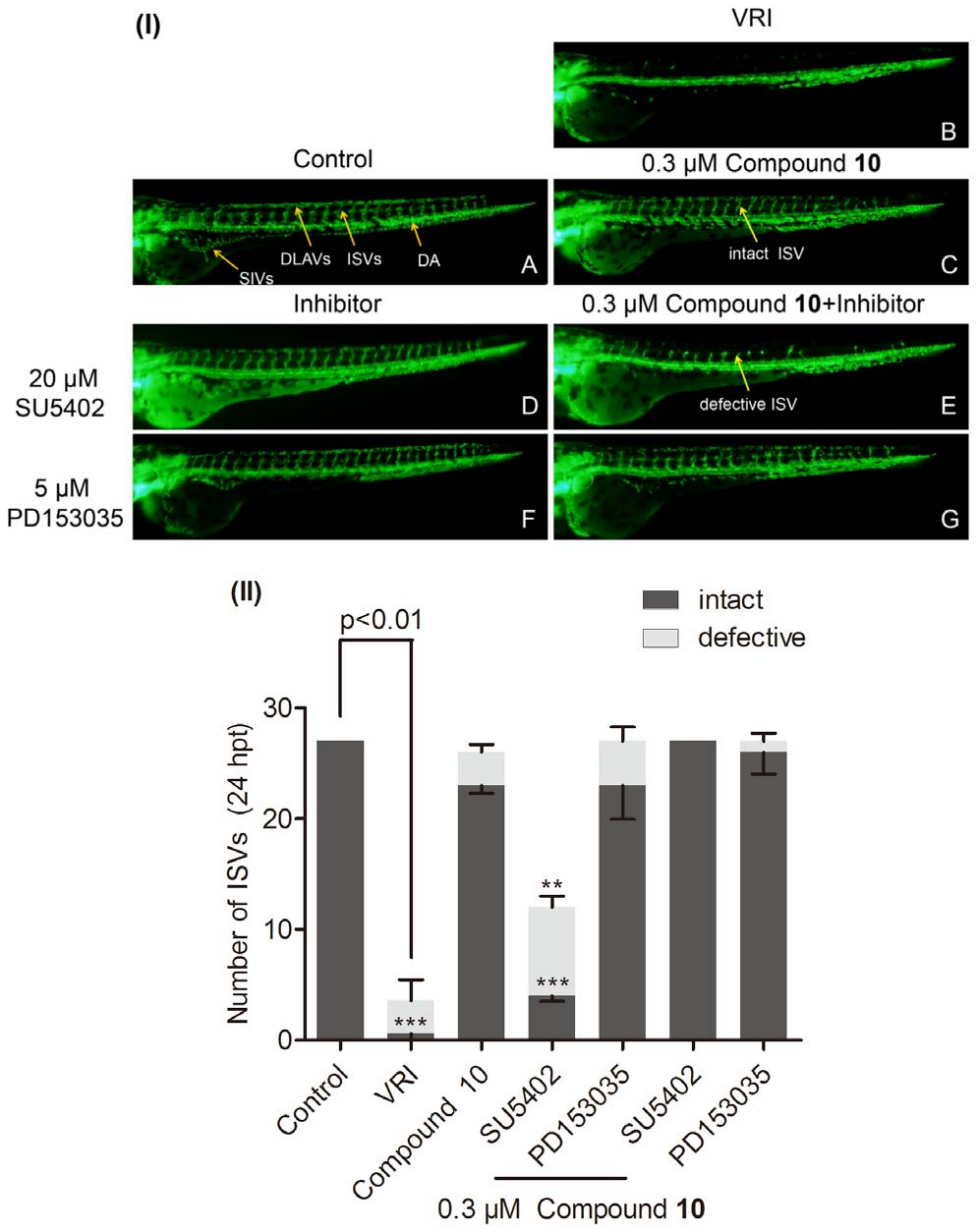
The effects of SU5402 and PD153035 on compound 10-induced angiogenesis in VRI-treated zebrafish. (I) 24 hpf zebrafish embryos were treated with 0.1% DMSO (v/v) (A), 20 µM SU5402 (D) and 5 µM PD153035 (F) for 24 h. 21 hpf embryos were treated with VRI (500 ng/ml) for 3 h. After that, the VRI was washed out and replaced with 0.1% DMSO (v/v) embryo medium (B) or 0.3 µM **10** (C), 0.3 µM **10** and 20 µM SU5402 (E), 0.3 µM **10** and 5 µM PD153035 (G) for another 24 h. Yellow arrows indicated that the ISVs (intact and defective), SIVs, DLAVs, and DA. (II) Quantitative analysis of inhibitory effect of SU5402 and PD153035 on **10**-induced angiogenesis in VRI-treated zebrafish. Data are expressed as mean±SEM, (n≧3). **p<0.01 and ***p<0.001 versus the **10** treatment group.

In order to confirm whether the angiogenesis response of compound **10** also involved epidermal growth factor (EGF) and fibroblast growth factor (FGF) signaling pathways, the effects of specific small molecule inhibitors on **10**-induced angiogenesis were studied. SU5402, a cell-permeable small molecule compound, is an ATP-competitive inhibitor of the FGFR1 tyrosine kinase [36], which has been shown to abolish FGF2-induced angiogenic effect on zebrafish embryonic development [37]. Both observable blood vessel phenotype change and quantitative analysis suggested that co-treatment of SU5402 (20 µM) with **10** for 24 h, almost entirely inhibited the restorative effect of **10** on blood vessels loss in VRI-treated zebrafish (Fig. 7E) while treatment with 20 µM SU5402 alone showed no significant effect on normal blood vessels development of zebrafish (Fig. 7D). In addition, PD 153035, a highly potent ATP-competitive and specific inhibitor of the tyrosine kinase activity of EGF receptor, failed to significantly block the vascular action of **10** in VRI-treated zebrafish (Fig. 7G). Therefore, our results suggested that activation of FGF, rather than EGF, might be essential for the angiogenesis effect of **10** in VRI-treated zebrafish (Fig. 7II).

### Effect of Inhibitors Downstream Signaling Targets of FGFR and VEGFR on Angiogenesis Effect of Compound 10 in VRI-treated Zebrafish

**Figure 8:**
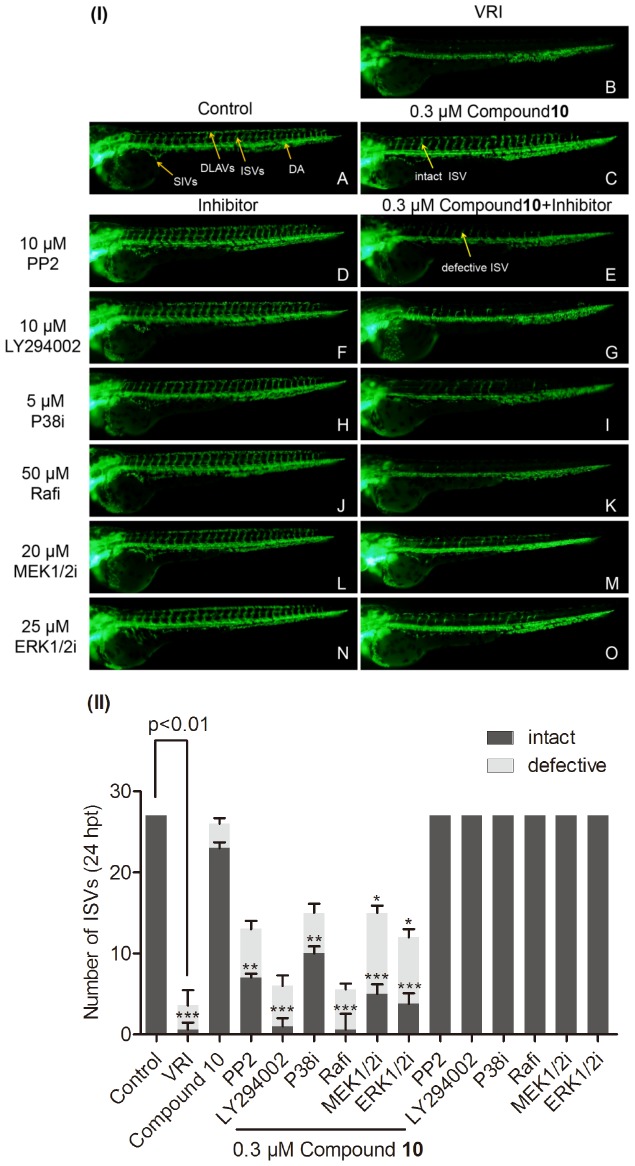
The effects of inhibitors of the downstream targets of FGF and VEGF signaling pathways on compound 10-induced angiogenesis in VRI-treated zebrafish. (I) 24 hpf zebrafish embryos were treated with 0.1% DMSO (v/v)(A),10 µM PP2 (D), 10 µM LY294002 (F),5 µM P38i (H), 50 µM Rafi (J),20 µM MEK1/2 (L),25 µM ERK1/2i (N) for 24 h. 21 hpf embryos were treated with VRI (500 ng/ml) for 3 h. After that, the VRI was washed out and replaced with 0.1% DMSO (v/v) embryo medium (B) or 0.3 µM **10** (C), 0.3 µM **10** and 10 µM PP2 (E), 0.3 µM **10** and 10 µM LY294002 (G), 0.3 µM **10** and 5 µM P38i (I), 0.3 µM **10** and 50 µM Rafi (K), 0.3 µM **10** and 20 µM MEK1/2i (M), 0.3 µM **10** and 25 µM ERK1/2i (O) for another 24 h. Yellow arrows indicated that the ISVs (intact and defective), SIVs, DLAVs, and DA. (II) Quantitative analysis of inhibitory effect of inhibitors of the downstream targets of FGF and VEGF signaling pathways. Data are expressed as mean±SEM, (n≧3). *p<0.05, **p<0.01 and ***p<0.001 versus the 10 treatment group.

**Figure 9:**
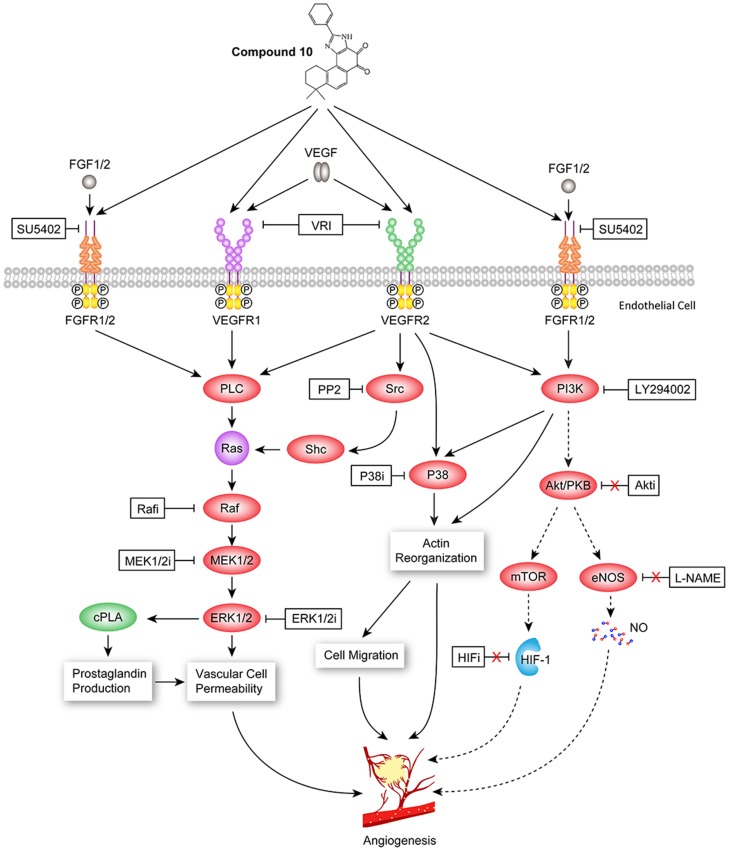
The proposed interaction of FGF and VEGF signaling pathways involved in compound 10-induced angiogenesis effects in VRI-treated zebrafish.

Previous studies suggested that PI3K, P38 and Raf/MEK/ERK are crucial downstream signaling targets that are involved in VEGF and FGF-induced angiogenesis [38], [39], [40]. Moreover, Src is required for the angiogenesis actions of VEGF/FGF, and VEGF/FGF-induced angiogenesis are inhibited by Src inhibitors [41], [42]. Therefore, we further investigated the roles of Src, PI3K, P38, Raf, MEK1/2, ERK1/2, Akt kinases, eNOS and HIF-1 in the angiogenesis effect of compound **10** by observing the effect of their selective pharmacological inhibitors in the zebrafish angiogenesis assay. PP2, a Src kinase inhibitor, acts as a potent and selective inhibitor of the Src family of protein tyrosine kinases [43]. Besides, LY 294002 is commonly used to inhibit PI3K activity which acts on the ATP binding site of PI3K [44]. Our results showed that, eleven inhibitors (Table 2) including PP2, LY294002, P38i, Rafi, ERK1/2i, MEK1/2i could reverse vascular restorative effect of **10** in zebrafish with VRI-treated blood vessels loss (Fig. 8I). Under the same treatment condition, HIFi, L-NAME and three Akti, all failed to abolish the vascular restorative effect of **10**. Meanwhile, in the blank control, treatment at 24 hpf with 10 µM PP2, 10 µM LY 294002, 5 µM P38i, 50 µM Rafi, 25 µM ERK1/2i and 20 µM MEK1/2i for 24 h did not significantly alter normal blood vessels development in zebrafish (Fig. 8II). Hence, our results suggest that Src, Raf, ERK1/2, MEK1/2, P38 and PI3K, but not Akt, eNOS-NO and HIF-1 pathways, were probably involved in the angiogenesis effect of **10** (Fig. 9).

**Table 2:** The effects of inhibitors of EGF, VEGF and FGF signaling pathways on compound **10**-induced angiogenesis in VRI-treated zebrafish.

Inhibitors	The rate of inhibition effect (%)		
PD153035	1 µM ○	5 µM ○	10 µM Toxic^a^
SU5402	10 µM ○	20 µM +++	50 µM ++++,Toxic^a^
PP2	5 µM ++	10 µM +++	20 µM Toxic^a^
LY294002	5 µM+++	10 µM ++++	20 µM ++++
L-NAME	100 µM ○	500 µM ○	1 mM +
P38i	2 µM +	5 µM ++	10 µM Toxic^a^
Rafi	10 µM ++	50 µM ++++	100 µM Toxic
ERK1/2i	10 µM ++	25 µM +++	50 µM +++,Toxic^a^
MEK1/2i	5 µM ++	10 µM ++	20 µM +++
Akti V	10 µM ○	30 µM ○	100 µM Toxic^a^
Akti IX	10 µM ○	30 µM ○	100 µM Toxic^a^
Akti IV	10 µM ○	30 µM Toxic^a^	100 µM Toxic^a^
HIFi	10 µM ○	30 µM ○	100 µM○

The semi-quantitative analysis of effects of inhibitors on angiogenesis activity of 10 in VRI-treated zebrafish: ○, inactive, < 20% inhibition rate; ++++, > 80%; +++, 60–80%; ++, 40–60%; +, 20%–40% as compared to the **10** post-treated zebrafish embryos. ^a^Zebrafish embryos treated with the indicated concentration of inhibitor had been showed toxicity and the inhibitory effect was not determined.

### Anti-proliferation Effects of Compound 10 Against MCF-7, MDA-231 and A549 Cell Lines

**Figure 10:**
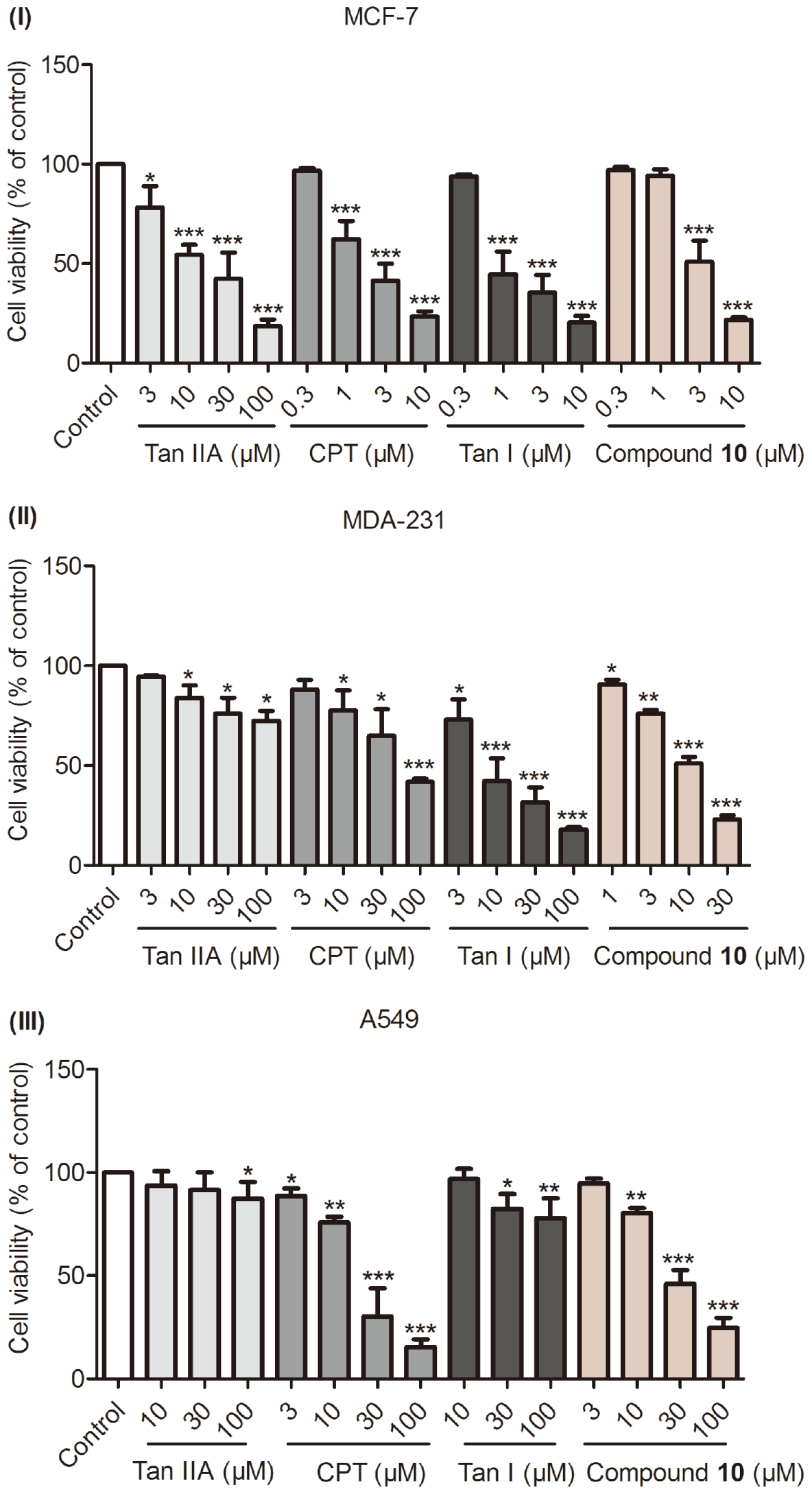
The anti-proliferation effects of compound 10 against three cancer cells compared to Tan IIA, CPT and Tan I. Cancer cells were serum-starved for 24 h followed by incubation with **10**, Tan IIA, CPT and Tan I. After 48 h, changes in the level of cell proliferation were assessed using the MTT assay. Data are expressed as the percentage cell viability as mean±SEM, (n≧3). *p<0.05, **p<0.01 and ***p<0.001 versus control (0.1% DMSO).

Although angiogenesis therapy is widely recognized as an effective mean of restoring various ischemic pathological conditions, it also raises a safety concern regarding the promotion cancer cell growth. Thus, in present study, we intended to identify compound **10** whether only elicited an angiogenesis effect under pathological conditions of angiogenesis deficiencies such as ischemic cerebral and heart diseases or not. In addition, the anti-cancer activities of numerous natural tanshinones had been well characterized in several pre-clinical studies [45], [46], [47]. Therefore, we performed a pilot study testing the anti-proliferation effect of **10** compared to Tan IIA, CPT and Tan I in three cancer cell lines (MCF-7, MDA-231 and A549). The results revealed that **10** exhibited cytotoxic activities against MCF-7 (IC_50_ =  3.3 µM), MDA-231 (IC_50_ =  6.5 µM) and A549 (IC_50_ =  17.9 µM) (Table 3). It is noteworthy that the minimal effective concentration of **10** is 1–3 µM in MCF-7, 1 µM in MDA-231, 3–10 µM in A549, respectively (Fig. 10).

**Table 3:** The IC_50_ values of compound **10**, Tan IIA, CPT and Tan I against MCF-7, MDA-231, A549 cancer cell lines.

Compounds(µM)	MCF-7	MDA-231	A549
Tan IIA	8.1	>100	>100
CPT	1.5	35.4	17.5
Tan I	1.1	4	>100
**10**	3.3	6.5	17.9

## Discussion

In our study, a series of new tanshinone derivatives were successfully synthesized from a mixture of natural tanshinones through one-pot synthesis approach. The present pilot study, for the first time, proved the concept of feasibility using a modified one-pot synthesis to modify minor tanshinones, which are hardly to be isolated with sufficient quantity for chemical modification by conventional organic synthesis approach. We found that some of the new tanshinone derivatives exhibited strong protective and restorative effects against blood vessels loss in zebraifsh. And the newly identified derivatives could serve as new lead candidates for further development as angiogenesis therapeutic agents.

Tanshinones are a group of diterpenoid quinones with unique structural scaffolds and promising biological activities. However, a substantial number of tanshinones which are present in very low quantities in *Salvia miltiorrhiza* [48] constitute as a hurdle in researching on their conventional organic modification because purification of those minor tanshinones are difficult. In previous study, one-pot combinatorial modification was used to improve the drug-like properties of tanshinones by incorporating a N-containing heterocyclic ring which is an important moiety in many drug molecules [49]. 6-mercaptopurine and thioguanine [50] are two examples of anti-cancer drugs which have an N-containing heterocyclic ring. Interestingly, our rapid one-pot synthesis of total tanshinone fraction leaded to isolate and identify eleven new derivatives which exhibited different degrees of angiogenesis activities. In this study, we also proved that the use of a modified one-pot synthesis to enhance the chemical diversity and biological activities of natural products was feasible.

Angiogenesis deficiencies are associated with various human diseases (e.g. cardio-cerebrovascular problems and ulcers). Our zebrafish angiogenesis assay which was produced by inactivation of the VEGF signaling pathway was more physiological relevant assay for identifying pro-angiogenesis agents against diseases associated with angiogenesis deficiencies [30]. In present study, screening of all chemically modified tanshinone derivatives in VRI-treated zebrafish, led to the identification of compound **10** as the most potent and effective angiogenic compound for restoring blood vessels damage which may have been the result of improvement in either one or a combination of various drug-like properties. Further studies to investigate the change of pharmacodynamic and pharmacokinetic properties of **10** were warranted. In addition, the new tanshinone derivatives exhibited limited close relatedness in their structures as they evolved from the random modification of different natural tanshinones. Therefore, no conclusive relationship between their structures and angiogenesis activity in VRI-treated zebrafish could be drawn.

It has been reported that the formation of new blood vessels is orchestrated by different proteins, including cell adhesion molecules, extracellular matric components and vascular endothelial growth factor receptors (VEGFRs) [51]. The VEGFRs play crucial roles in transducing regulatory signal from VEGF which was originally described as a potent angiogenic factor as well as an essential growth factor for vascular endothelial cells [52]. Although our gene expression data provided clues on the possible involvement of the VEGF-KDR/FLK pathway in restoring blood vessels after **10**-treatment, these results alone did not entirely reflect the activation of the downstream signaling targets (Src, PI3K, P38 and MAPK signaling pathways) involved in VEGF/FGF-induced angiogenesis because their activation usually occurs via phosphorylation in post-translation regulation [53]. In order to address this aspect, we determined the effects of specific small molecule inhibitors against key downstream targets of EGF, FGF and VEGF signalling pathways on the pro-angiogenesis effect of **10**. Our results demonstrated that **10** probably promoted angiogenesis through VEGF and/or FGF by activating Src, PI3K, P38, Raf, MEK1/2 and ERK1/2 kinases but not HIF-1, eNOS and Akt kinases. Taken together, all these evidences showed that the angiogenesis activity of **10** probably involved the interaction of VEGF/FGF-Src-MAPK and PI3K-P38 signaling pathways in VRI-treated zebrafish (Fig. 9).

In sum, the above data indicated that a novel one-pot combinatorial modification was used to simultaneously diversify both the major and minor natural tanshinones which led to the isolation and identification of eleven new derivatives. In the present study, our results suggested that **10**, the most potent new angiogenic tanshinone derivative identified without any promoting proliferation effects in three cancer cells, had potential for further development as a therapeutic agent for diseases associated with imbalanced angiogenesis such as ischemic heart disease and traumatic injuries. In addition, both the mRNA expression assay and blocking assay of specific kinases inhibitors provided insights into the mechanism underlying the angiogenesis effect of **10**. It is worthy of note that the zebrafish screening model used in this study is based on embryonic development, but not a real pathology model. Further investigation of the pharmacodynamic data of **10** based on the vascular injury pathological model was warranted. In addition, physiological angiogenesis is a vital process in growth and development of organism. Since the process is complicated, and typically consists of cell proliferation, migration, invasion and tube formation [54], we further investigated if **10** could directly act on either one of the biological steps in angiogenesis on human umbilical vein endothelial cells (HUVECs). In our results, **10** could significantly promote the migration and invasion of HUVECs in a concentration-dependent manner, as effective as the effects of VEGF. However, the HUVECs proliferation stimulating effect of **10** was mild when compared with that of VEGF (Section 8 in File S1). These in vitro findings further confirm our observation of angiogenic effect of **10** in zebrafish and provide further insight into pharmacological action of **10** indeed involving direct cellular action, at least on the two crucial steps of migration (Figure S15 in File S1) and invasion (Figure S16 in File S1) underlying angiogenesis.

In conclusion, one-pot modification of the total tanshinone fraction from *Salvia miltiorrhiza* could successfully generate new chemical structures with higher biological activity, of which **10**, a novel angiogenic compound, was a good example. More importantly, our study should have a high impact in this research field as it provided valuable information on an alternative, relatively fast and green approach for the discovery of novel diversified compounds, particularly those low abundant components present in natural products.

## Supporting Information

File S1 **Supporting Information.** Section 1. A Stack Plot Showing the HPLC Chromatograms of Compounds 1-11. Section 2. Detailed Discussion of the Structure Elucidation of New Derivatives. Section 3. Original Mass Spectra and NMR Spectra of Compounds 1-11. Section 4. Table S1. Crystal data and structure refinement for compounds 1, 2, 3, 6 and 10. Section 5. Structural Formulae of Natural Parental Tanshinones 1a-11a. Section 6. Configurations of Natural Parental Tanshinones 1a-11a. Section 7. Table S2. The EC_50_ and IC_50_ values of compounds 3, 10, Tan IIA, CPT and Tan I in VRI-treated zebrafish. Section 8. Effects of Compound 10 on the Proliferation, Migration and Invasion of HUVECs.
(DOCX)
