# Supplementary material for: In Vivo Angiogenesis Screening and Mechanism of Action of Novel Tanshinone Derivatives Produced by One-Pot Combinatorial Modification of Natural Tanshinone Mixture from Salvia Miltiorrhiza
Source: PLoS One. 2014 Jul 3;9(7):e100416. doi: 10.1371/journal.pone.0100416 (PMC4081027; doi:10.1371/journal.pone.0100416)
Supplement: File S1 — Supporting Information. Section 1. A Stack Plot Showing the HPLC Chromatograms of Compounds 1-11. Section 2. Detailed Discussion of the Structure Elucidation of New Derivatives. Section 3. Original Mass Spectra and NMR Spectra of Compounds 1-11. Section 4. Table S1. Crystal data and structure refinement for compounds 1, 2, 3, 6 and 10. Section 5. Structural Formulae of Natural Parental Tanshinones 1a-11a. Section 6. Configurations of Natural Parental Tanshinones 1a-11a. Section 7. Table S2. The EC50 and IC50 values of compounds 3, 10, Tan IIA, CPT and Tan I in VRI-treated zebrafish. Section 8. Effects of Compound 10 on the Proliferation, Migration and Invasion of HUVECs. (DOCX) [file pone.0100416.s001.docx]

*In vivo* Angiogenesis Screening and Mechanism of Action of Novel Tanshinone Derivatives Produced by One-pot Combinatorial Modification of Natural Tanshinone Mixture from *Salvia Miltiorrhiza*

**Zhe-Rui Zhang^1^☯, Jin-Hang Li^2^☯, Shang Li^1^, Ai-Lin Liu^1,3^, Pui-Man Hoi^1^,Hai-Yan Tian^2^, Wen-Cai Ye^2^, Simon Ming-Yuen Lee^1^* and Ren-Wang Jiang^2^***

**1** State Key Laboratory of Quality Research in Chinese Medicine and Institute of Chinese Medical Sciences, University of Macau, Macao, P.R. China, **2** College of Pharmacy, Jinan University, Guangzhou, P.R. China, **3** Institute of Materia Medica, Chinese Academy of Medical Sciences and Peking Union Medical College, Beijing, P. R. China

**List of Supporting Information**

**Section 1. A Stack Plot Showing the HPLC Chromatograms of Compounds 1-11.**

**Section 2. Detailed Discussion of the Structure Elucidation of New Derivatives.**

**Section 3. Original Mass Spectra and NMR Spectra of Compounds 1-11.**

**Section 4. Table S1. Crystal data and structure refinement for compounds 1, 2, 3, 6 and 10.**

**Section 5. Structural Formulae of Natural Parental Tanshinones 1a-11a.**

**Section 6. Configurations of Natural Parental Tanshinones 1a-11a.**

**Section 7. Table S2. The EC_50_ and IC_50_ values of compounds 3, 10, Tan IIA, CPT and Tan I in VRI-treated zebrafish.**

**Section 8. Effects of Compound 10 on the Proliferation, Migration and Invasion of HUVECs.**

**Section 1: A Stack Plot Showing the HPLC Chromatograms of Compounds 1-11.**


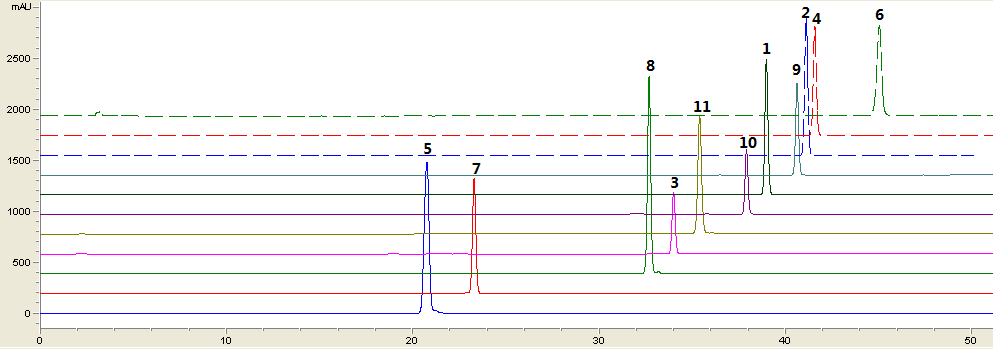


**Figue S1 in File S1. A stack plot showing the HPLC chromatograms of compounds 1-11.**

**HPLC conditions:**

Instrument: Agilent HPLC 1200 system equipped with an auto-sampler and a PDA detector. Column Welch Materials Column-XB-C 18 (4.6x250mm, 5μm) was used.

Mobile phases: A: H_2_O and B: acetonitrile (Gradient: 0-40min 50%-100%B, 40-50min 100%B); flow rate: 1 ml/min; detection at 270 nm.

**Section 2:** Detailed Discussion of the Structural Elucidation of New Derivatives.

The eleven new derivatives (compounds **1**-**11**) which could be classified into three groups.

The first group included **1**-**5**, which was constructed by the 2-phenylimidazole and C_18_ tanshinone moieties. Compound **1** was isolated as colorless prismatic crystals. HRESIMS analysis of **1** showed a quasi-molecular ion peak at *m/z* 363.1510 [M+H]^+^ (calcd for 363.1492), corresponding to a molecular formula C_25_H_18_N_2_O. The IR spectrum showed no absorptions around 1700 cm^-1^ indicating that the *o*-quinone moiety was disappeared after reaction.

The ^1^H NMR spectrum of **1** (Section 3 of File S1) showed the presence of a benzylimidazole moiety: δ 8.45 (2H, d, J= 7.2 Hz), δ 7.54 (2H, dd, J= 7.2, 7.6 Hz) and δ 7.54 (1H, t, J= 7.6 Hz) attributable to the monosubstituted benzene and δ13.04 attributable to the N-H in the imidazole ring, and a tanshinone moiety: five aromatic methines in rings A and B (δ10.87, 1H, d, J= 8.4 Hz, H-1; 7.72, 1H, dd, J= 7.2, 8.4 Hz, H-2; 7.54, 1H, d, J= 7.6 Hz, H-3; 8.13,1H, d, J= 9.2 Hz, H-6; 8.37, 1H, d, J= 9.2 Hz, H-7) and a methyl (2.66, 3H, s, H-17) and an oxygenated methine (8.02,1H, s, H-16) in the furan ring. The proton signals of the tanshinone moiety are similar to Tan I except that H-1 shift to a significantly lower field, which might be explained by the fact that H-1 occupies the deshielding zone of the benzene ring and might form hydrogen bond with the nitrogen in the imidazole ring.

The ^13^C NMR and HMQC spectra confirmed the presence of 2-phenylimidazole and tanshinone moieties in the molecular skeleton. Detailed NMR data assignments were done through analysis of its 2D NMR spectra. Finally, suitable crystals were obtained from the ethanol solution. The subsequent X-ray diffraction experiments revealed the complete structure and conformation. Compound **1** crystallized as an ethanol monosolvate (Fig. 3). The molecule is essentially planar with a mean deviation 0.107 Å. Accordingly, **1** could be identified as phenylimidazoletanshinone I.

The ^1^H and ^13^C-NMR spectra of **2** were similar to those of **1**, except that C-1 and C-2 were changed to methylenes (H-1: δ4.25, 2H, t; C-1: δ22.8 t; H-2: δ 2.36, 2H, m; and C-2: 24.8, t). The molecular structure of **2** was also confirmed by X-ray crystallographic analysis (Fig. 3), which showed 1:1 DMSO solvate linked through a N-H⋅⋅⋅O hydrogen bond. Accordingly, compound **2** could be identified as 4,9-dimethyl-2-phenyl-11,12-dihydro-3H-6-oxa-1,3-diaza-dicyclopenta [a,c] phenanthrene,and could be accorded the trivial names 1,2-dihydrophenylimidazoletanshinone I (**2**).

Compound **3** also showed similar ^1^H and ^13^C-NMR spectra to those of **1** except that the signals for H-15 (δ4.07, 1H, m), H-16 (δ4.96, 1H, t, J= 9.0 Hz; δ4.50,1H, dd, J= 4.5, 4.2 Hz) and H-17 (δ1.53, 3H, d, J= 6.6 Hz) were shifted to higher field, which suggested that **3** was an analog of **1** with the only difference being the absence of Δ^15,16^ double bond. In order to establish the absolute configuration of C-15, we tried to develop suitable crystals for X-ray diffraction experiment. Finally, the complete structure and stereochemistry were established as its methanol monosolvate. The final refinement on the CuKα data resulted in a small Flack parameter of 0.01 (4), allowing the unambiguous assignment of the absolute configuration (Fig. 3).

The ^1^H and ^13^C-NMR spectra of **4** were again similar to those of **1**, except that the three aromatic methines in ring A of **1** were replaced by three alicyclic methylenes (4.09, 2H, t; 2.04, 2H, m; 2.61, 2H, m). In addition, the exo-methylene protons were revealed by the signals at δ5.07 (1H, br s, H-18α) and δ5.65 (1H, br s, H-18β) in the ^1^H -NMR spectrum and δ109.5 in ^13^C-NMR spectrum. Accordingly, **4** can be identified as: 4-methyl-9-methylene-2-phenyl-9,10,11,12- tetrahydro-3*H*-6-oxa-1,3-diaza-dicyclopenta [*a,c*] phenanthrene, and could be accorded the trivial names methylenephenylimidazoletanshinquinone.

The structure of **5** is similar to **4** except that the exo-methylene moiety in **4** was replaced by a hydroxyl and a hydroxymethylene groups which were evident from the ^1^H (δ4.01, 1H, d, J= 6.6Hz, H-18α; δ3.77, 1H, m, H-18β) and ^13^C-NMR signals (δ69.2, s, C-4; 71.3, t, C-18).

The second group of **6**-**9** is constructed by the 2-phenylimidazole and C_19_ tanshinone moieties. Compound **6** is a typical example of this group. Similar to **1**, no carbonyl signals were observed in the ^13^C NMR (Section 3 of File S1) and IR spectrum, indicating that the *o*-quinone moiety was disappeared after the reaction. The ^1^H and ^1^C NMR data showed a phenylimidazole moiety with signals similar to that of **1** and a tanshinone moiety with characteristic signals for three methyl groups (δ2.66, 3H, s, H-17; δ1.37, 6H, s, H_3_-18 and H_3_-19; δ9.7, q, C-17; δ32.0, q, C-18 and 19), three methylenes (δ3.96, 2H, t, J= 6.6Hz, H-1; 1.95, 2H, m, H-2; 1.75, 2H, m, H-3; 30.8, t, C-1; 19.6 t, C-2; 38.5 t, C-3) and one oxymethine (δ7.88, 1H, d, J= 1.0Hz, H-16, 141.2, d, C-16). The signals of the tanshinone moiety were close similar to those of tanshinone IIA except for the absence of carbonyl groups. Accordingly, compound **6** could be named as phenylimidazoletanshinone IIA.

Compounds **7**, **8** and **9** are similar to **6** constructed by the 2-phenylimidazole and C_19_ tanshinone moieties. Compound **7** is a 19-hydroxymethylene analog of **6**, and its structure was confirmed by the replacement of a methyl signal of **6** with a hydroxylated methylene signal (δ3.54, 1H, br.s, H-19α; 3.62, 1H, br.s, H-19β; 69.7, t, C-19). Compound **8** is a 19-methyl carboxylate analog of **6**, which is evident by the ^1^H (δ3.60, 3H, s, H-20) and ^13^C-NMR signals (177.5, s, C-19; 52.5, q, C-20). The ^1^H and ^13^C-NMR spectra of **9** were similar to those of **6**, except that the double bond Δ^15,16^ in the furan ring of **6** was saturated which is evident by the presence of an oxymethylene (δ4.87, 1H, t, J= 8.8 Hz, H-16α; 4.42, 1H, dd, J= 8.8, 4.4 Hz, H-16β; δ 78.7, t, C-16).

Compound **10** displayed a peseudomolecular ion [M+H]^+^ 357.1597, corresponding for a molecular formula of C_23_H_20_N_2_O_2_. X-ray diffraction analysis of **10** revealed a benzylimidazole coupled tricyclic framework (Fig. 4). The presence of *o*-quinone moiety was confirmed by the short bond distances (C13-O1 1.226Å; C14-O2 1.210Å) for the two carbonyl groups. The imidazole ring is roughly co-planar with quinone rings C and D with a dihedral angle 5.9°, while the phenyl ring F is twist and make a dihedral angle 18.1° with the least square plane of rings C and D. The NMR spectral data of **10** supported the structure derived from X-ray diffraction analysis. Absence of signal for the oxygenated methine or methylene protons indicated that there is no furan ring in **10** as compared with **1**-**9**. In contrast, the ^13^C NMR resonance at δ 182.0 (C-13) and δ 180.4 (C-13) confirmed the presence of two carbonyls as revealed by X-ray analysis.

HRESIMS analysis of **11** showed a pseudomolecular ion at *m/z* 384.1588 [M+H]^+^, corresponding to a molecular formula C_25_H_21_NO_3_. There is only one nitrogen atom in the molecule as compared with **1**-**10**, suggesting that the phenylimidazole might be replaced by a phenyloxazole ring, which was confirmed by the signals attributable to the monosubstituted benzene and absence of proton signal for the NH group. The ^1^H NMR spectrum of **11** revealed an ABX coupling pattern for three aromatic protons in ring A at *δ* 10.25 (d, *J*= 8.4 Hz), 7.72 (dd, *J*= 8.4, 6.6 Hz), and 7.56 (d, *J*= 6.6 Hz), an AB pattern for *ortho*-aromatic protons in ring B at *δ* 8.04 (d, *J*= 9.4 Hz) and 8.38 (d, *J*= 9.4 Hz), two methyl groups (*δ*1.51, d, *J*= 3.9 Hz, H-17; 2.77, s, H-18), a methine proton at *δ* 3.89 (1H, m, H-15), and a methylene group at *δ* 4.01 (t, *J*= 7.2 Hz, H-16α) and 4.04 (dd, *J*= 16.6, 7.2 Hz, H-16β). These signals were similar to those of neocryptotanshinone. Accordingly, **11** can be named as phenyloxazoleneocryptotanshinone.

**Section 3:** Original Mass Spectra and NMR Spectra of Compounds **1-11**.

**
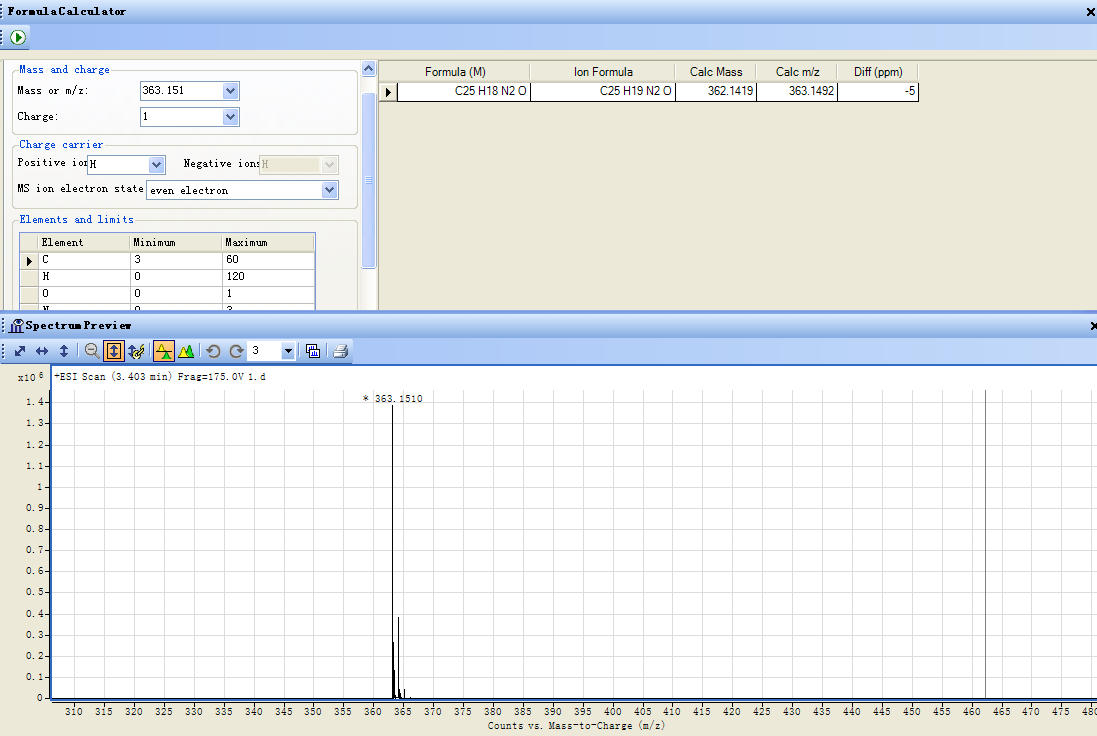
**

**Figure S2a in File S1. HR-ESI-MS of compound 1*.***
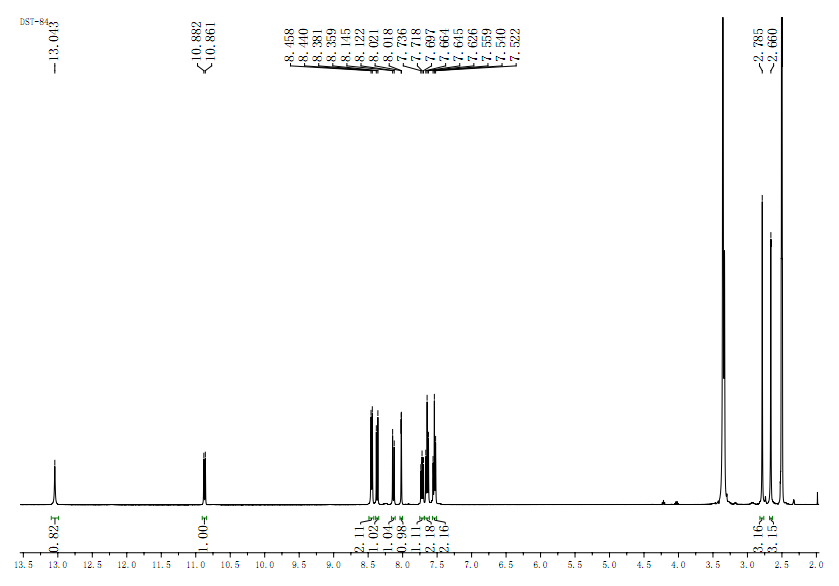


**Figure S2b in File S1. 1H NMR spectrum of compound 1 (300 MHz, DMSO-d6).**
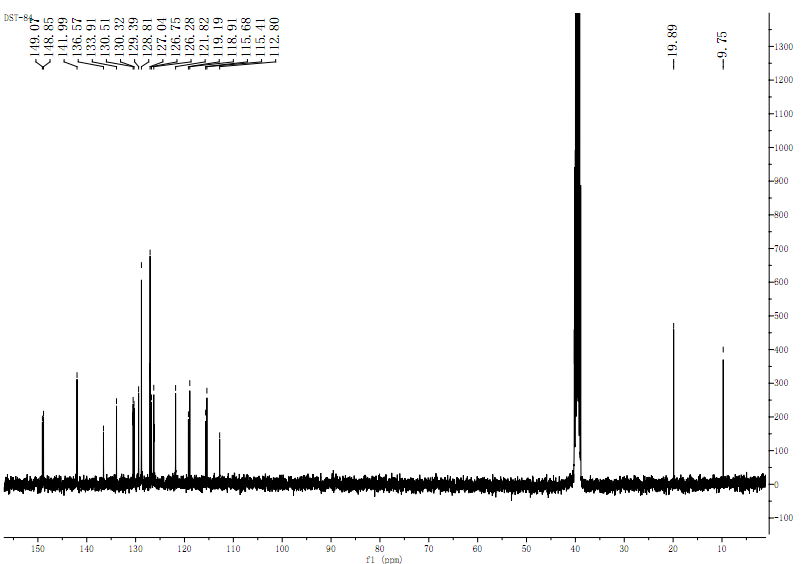


**Figure S2c in File S1. ^13^C NMR spectrum of compound 1 (75 MHz, DMSO-*d_6_*).**


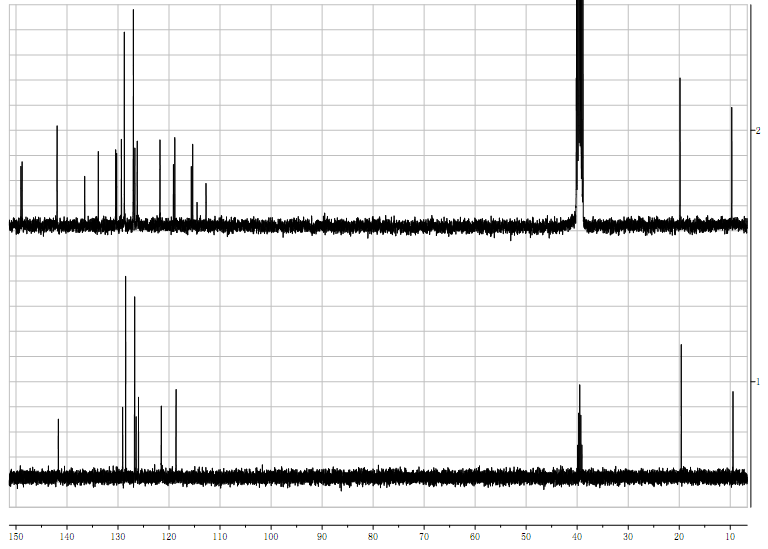


**Figure S2d in File S1. DEPT135 spectrum of compound 1 (DMSO-*d_6_*).**


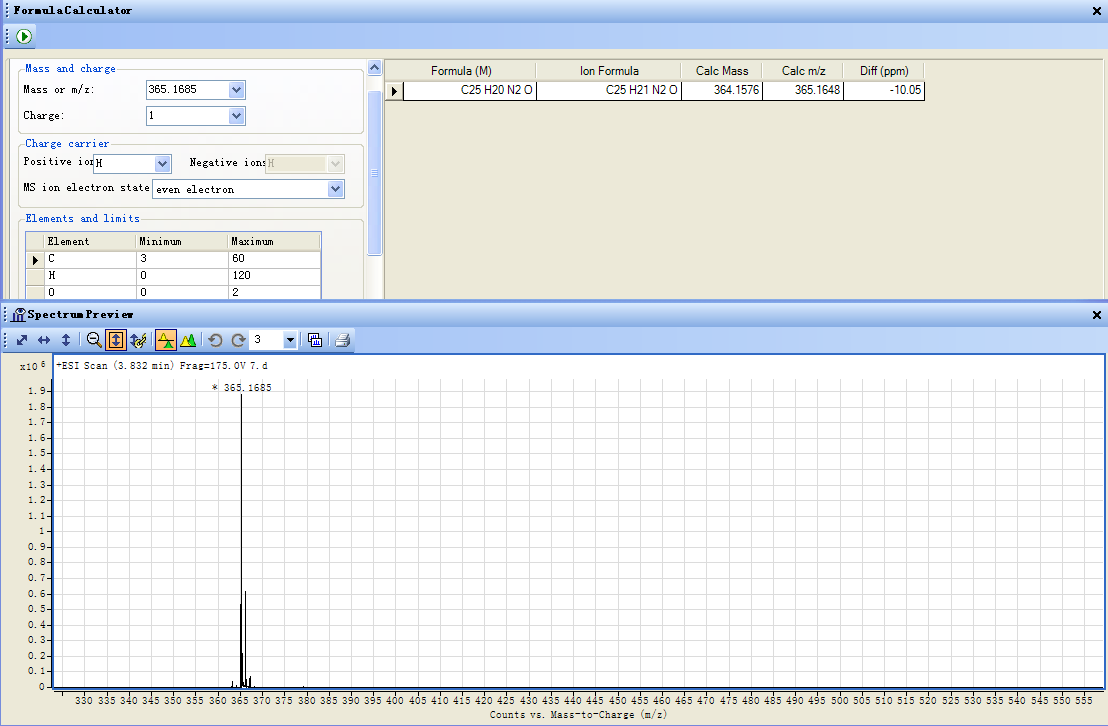


**Figure S3a in File S1. HR-ESI-MS of compound 2.**


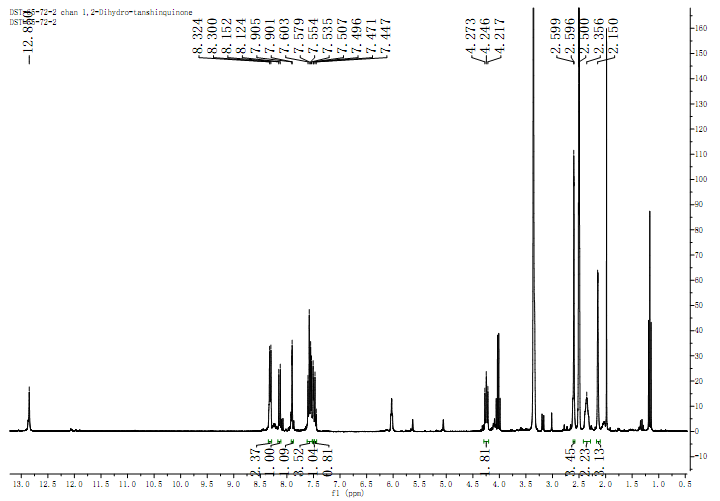
**Figure S3b in File S1 . ^1^H NMR spectrum of compound 2 (300 MHz, DMSO-*d_6_*).**

**
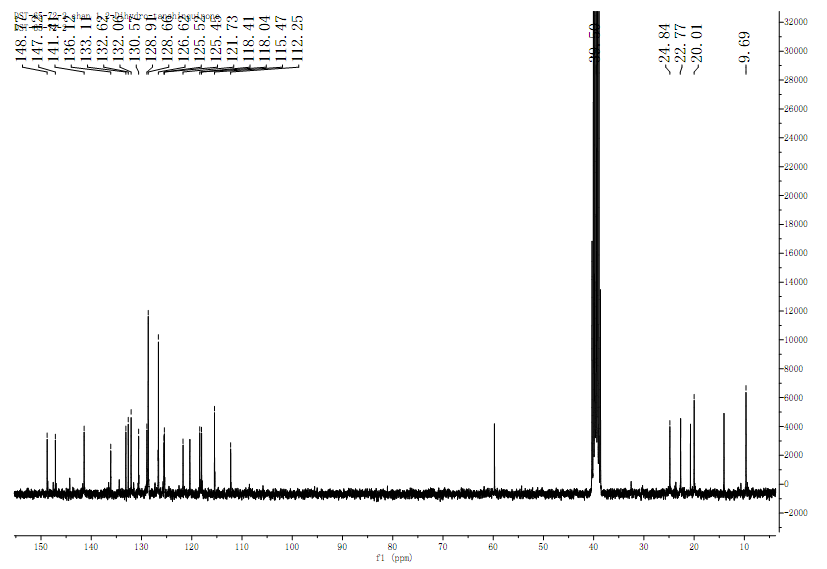
**

**Figure S3c in File S1. ^13^C NMR spectrum of compound 2 (75 MHz, DMSO-*d_6_*).**


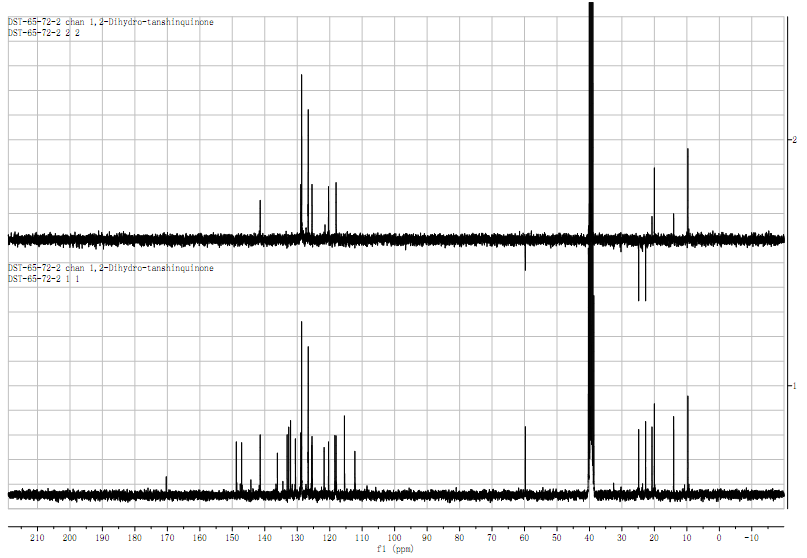


**Figure S3d in File S1. DEPT135 spectrum of compound 2 (DMSO-*d_6_*).**

**
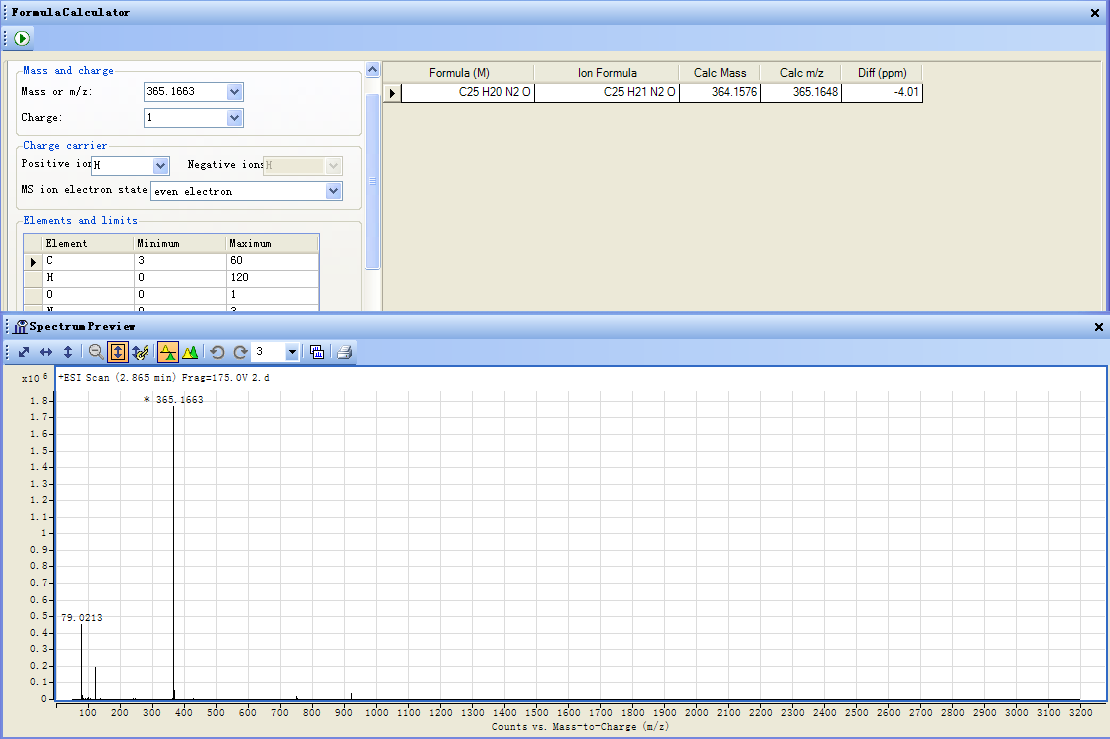
**

**Figure S4a in File S1. HR-ESI-MS of compound 3.**


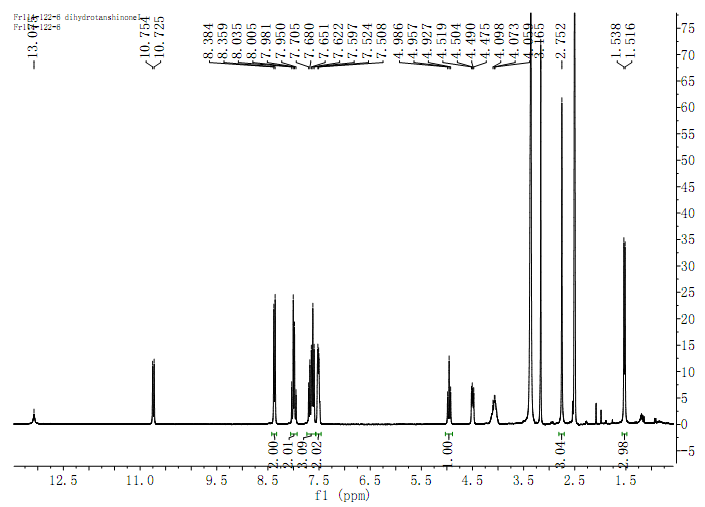


**Figure S4b in File S1. ^1^H NMR spectrum of compound 3 (300 MHz, DMSO-d_6_).**

**
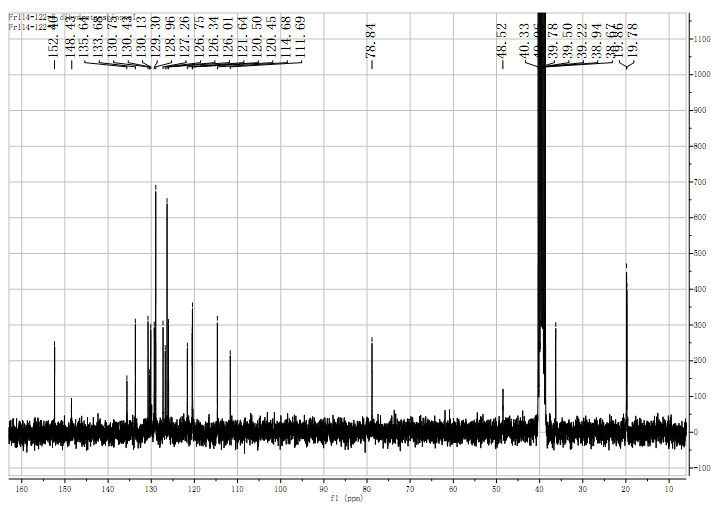
**

**Figure S4c in File S1. ^13^C NMR spectrum of compound 3 (75 MHz, DMSO-d_6_).**


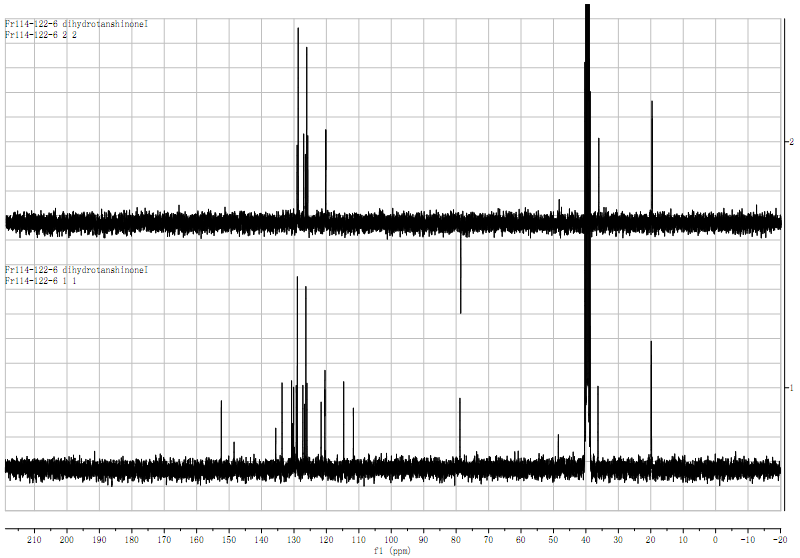


**Figure S4d in File S1. DEPT135 spectrum of compound 3 (DMSO-d_6_).**

**
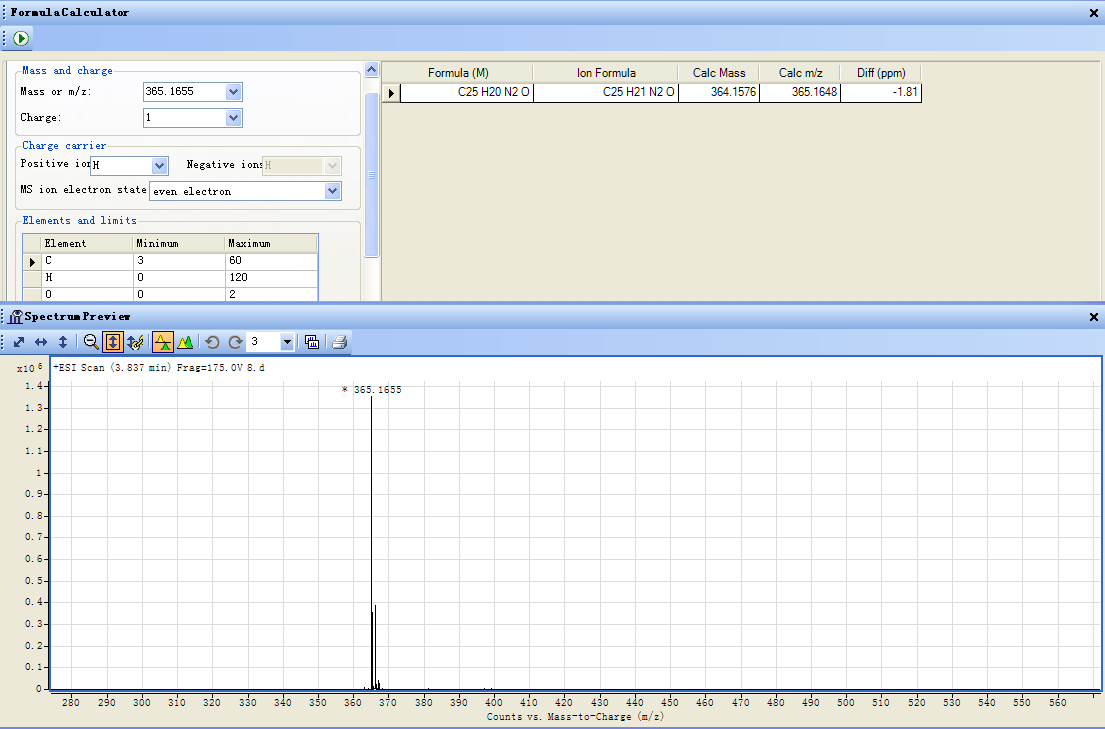
**

**Figure S5a in File S1. HR-ESI-MS of compound 4.**


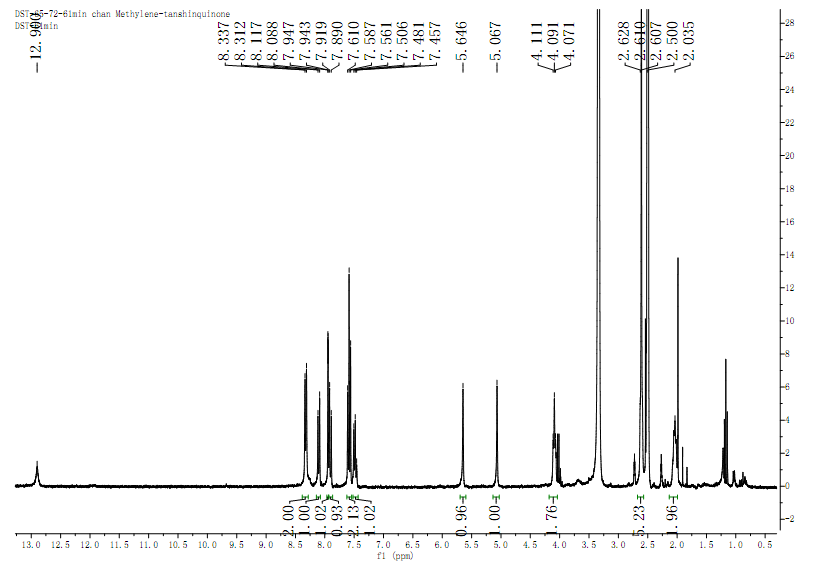


**Figure S5b in File S1. ^1^H NMR spectrum of compound 4 (300 MHz, DMSO-d_6_).**

**
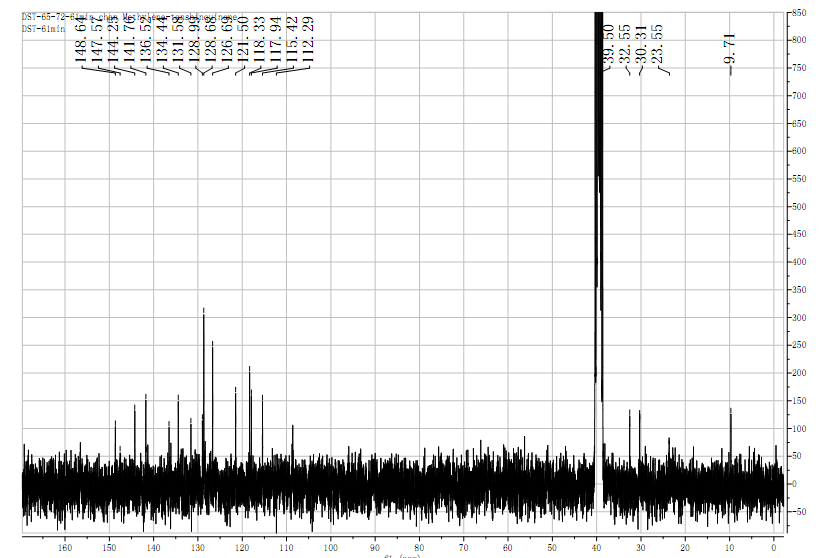
**

**Figure S5c in File S1. ^13^C NMR spectrum of compound 4 (75 MHz, DMSO-d_6_).**


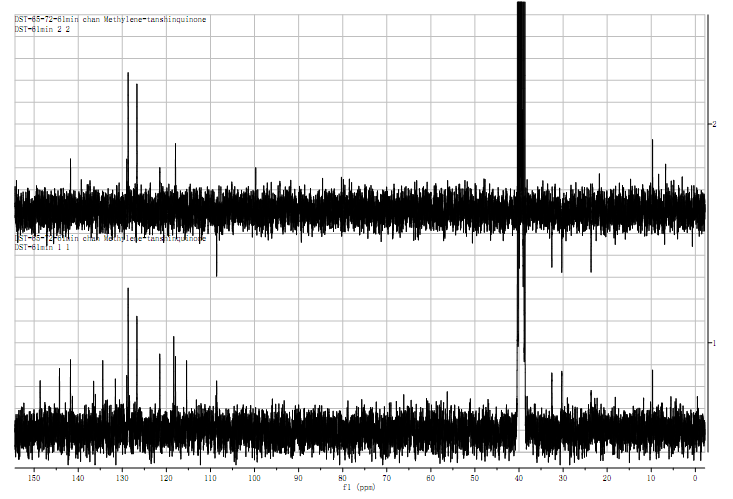


**Figure S5d in File S1. DEPT135 spectrum of compound 4 (DMSO-d_6_).**

**Figure S6a in File S1. ESI-MS of compound 5 (left: negative; right: positive).**


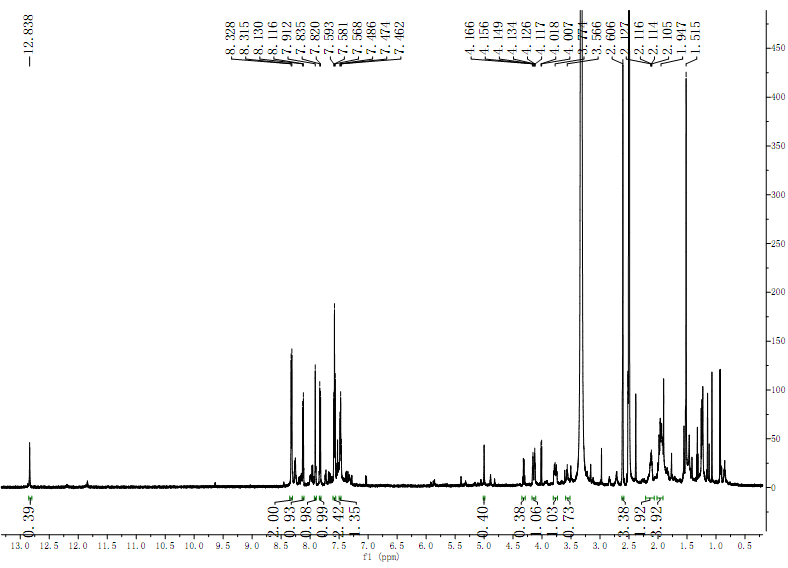


**Figure S6b in File S1. ^1^H NMR spectrum of compound 5 (300 MHz, DMSO-d_6_).**

**
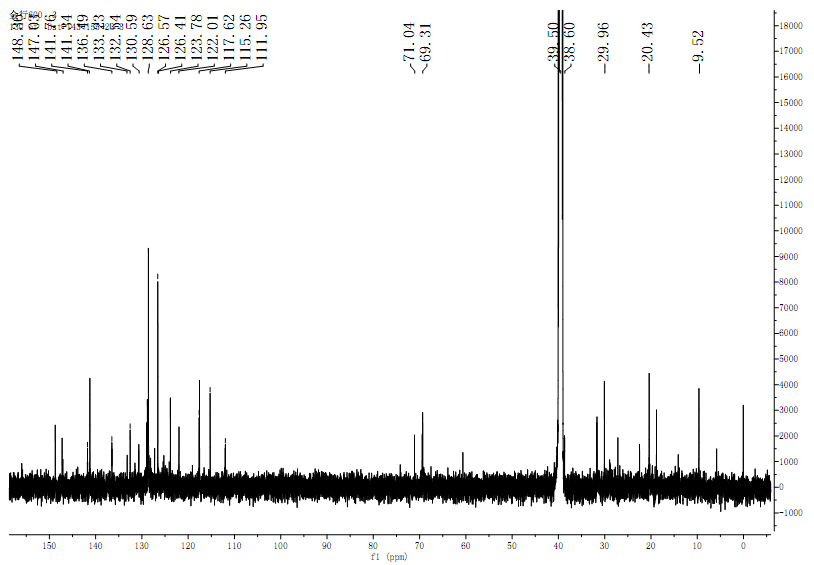
**

**Figure S6c in File S1. ^13^C NMR spectrum of compound 5 (75 MHz, DMSO-d_6_).**


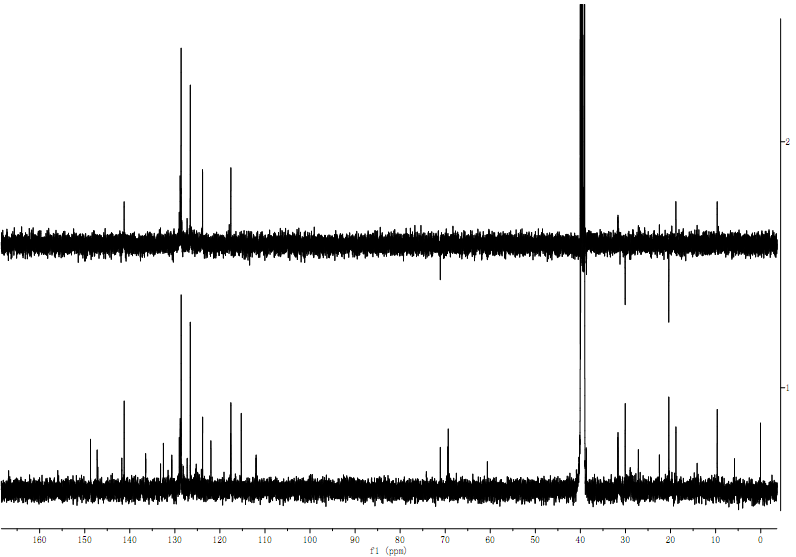


**Figure S6d in File S1. DEPT135 spectrum of compound 5 (DMSO-d_6_).**


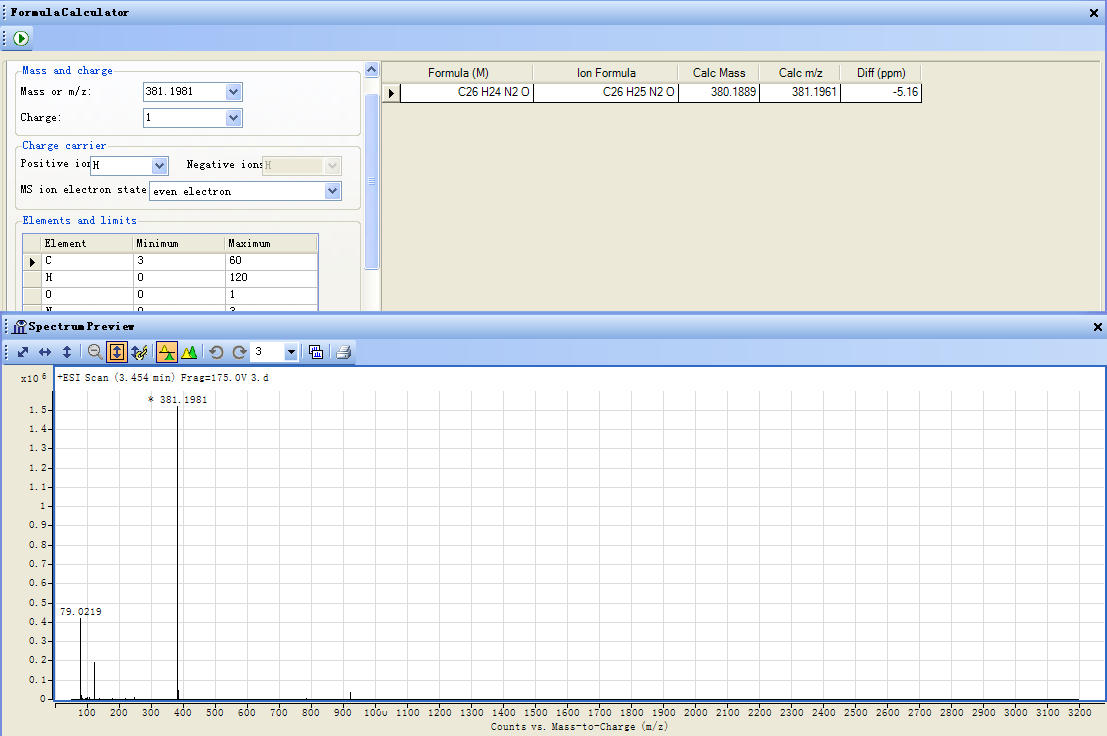


**Figure S7a in File S1. HR-ESI-MS of compound 6.**


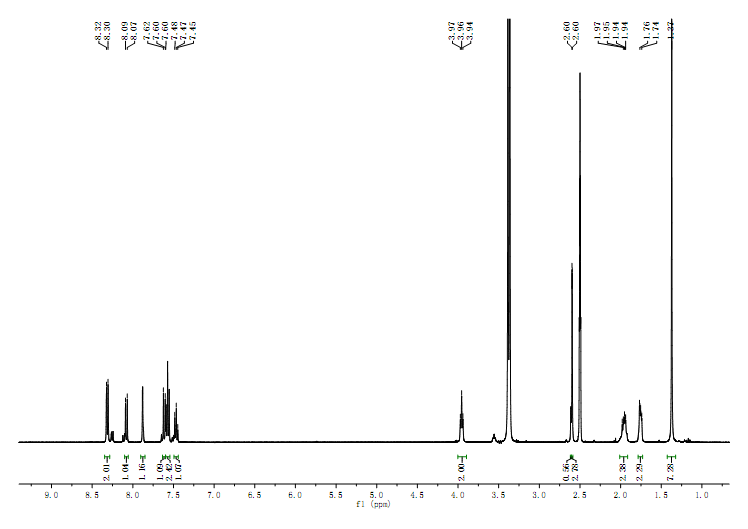


**Figure S7b in File S1. ^1^H NMR spectrum of compound 6 (300 MHz, DMSO-d_6_).**

**
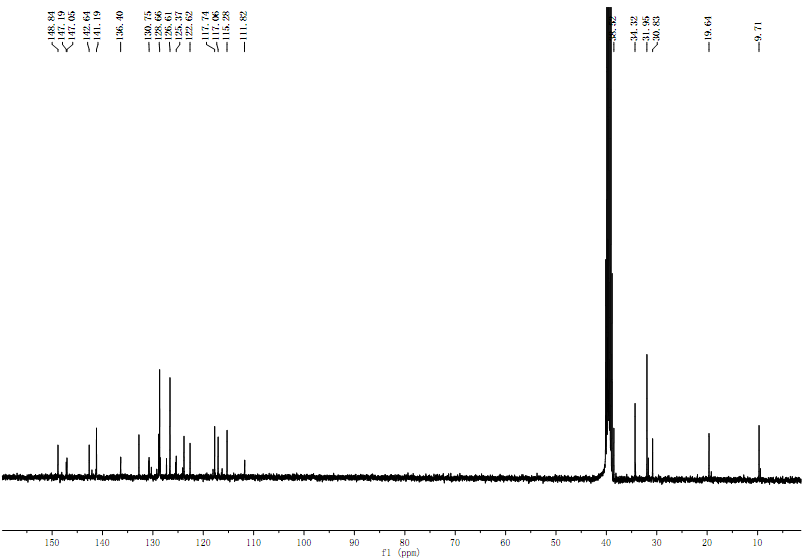
**

**Figure S7c in File S1. ^13^C NMR spectrum of compound 6 (75 MHz, DMSO-d_6_).**


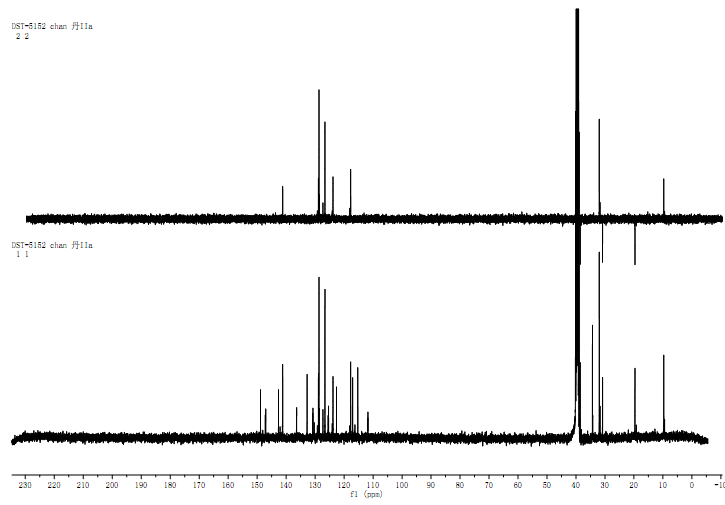


**Figure S7d in File S1. DEPT135 spectrum of compound 6 (DMSO-d_6_).**


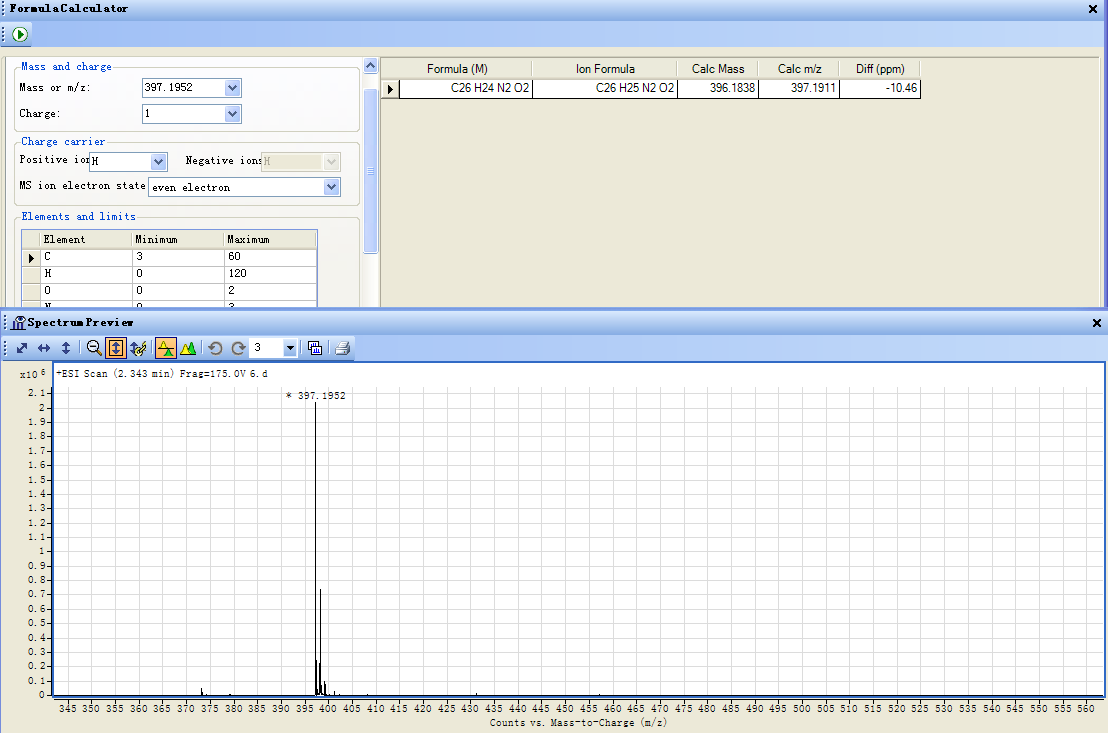


**Figure S8a in File S1. HR-ESI-MS of compound 7.**


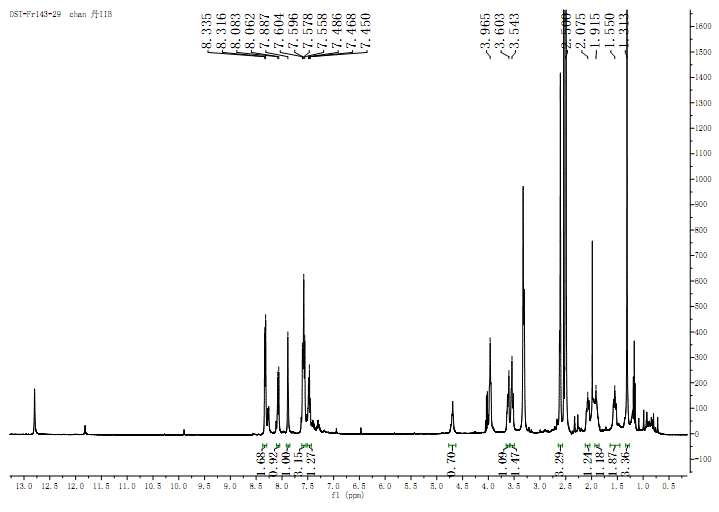


**Figure S8b in File S1. ^1^H NMR spectrum of compound 7 (300 MHz, DMSO-d_6_).**

**
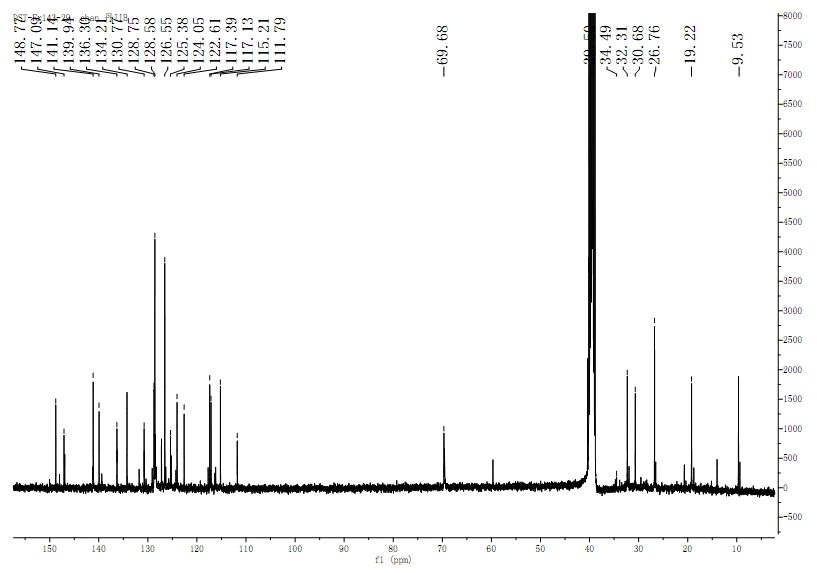
**

**Figure S8c in File S1. ^13^C NMR spectrum of compound 7 (75 MHz, DMSO-d_6_).**


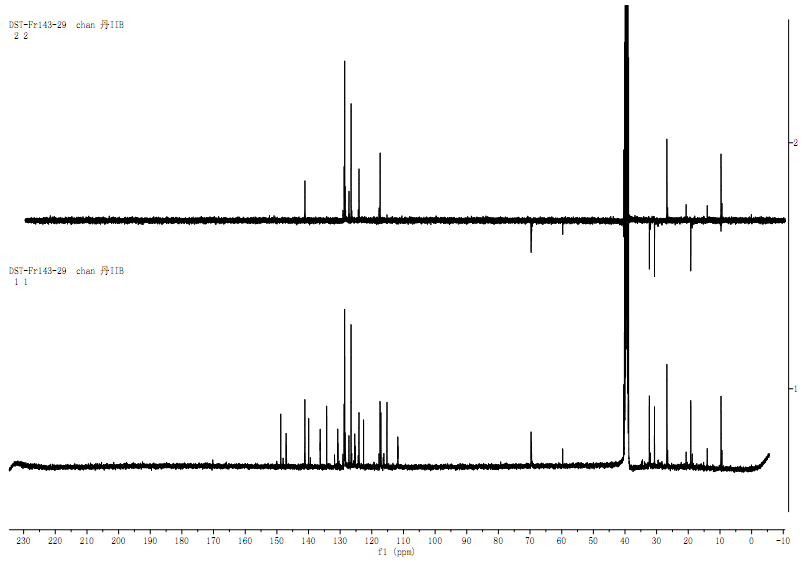


**Figure S8d in File S1. DEPT135 spectrum of compound 7 (DMSO-d_6_).**

**
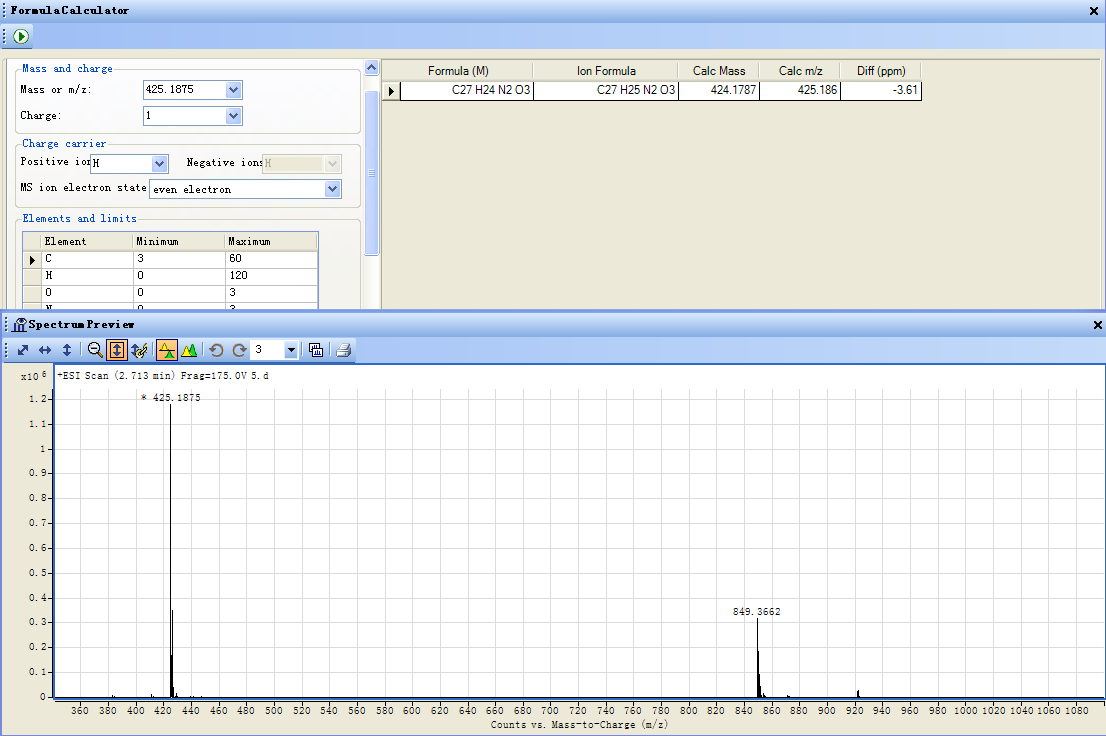
**

**Figure S9a in File S1. HR-ESI-MS of compound 8.**


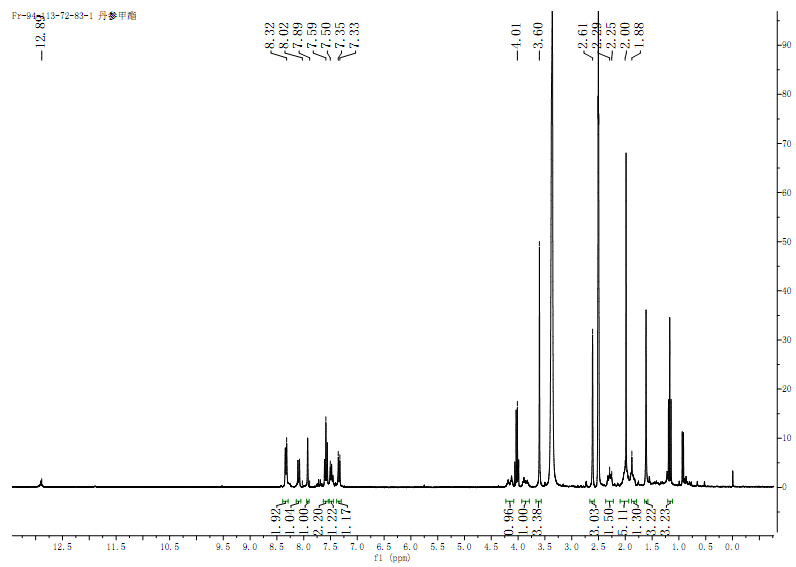


**Figure S9b in File S1. ^1^H NMR spectrum of compound 8 (300 MHz, DMSO-d_6_).**

**
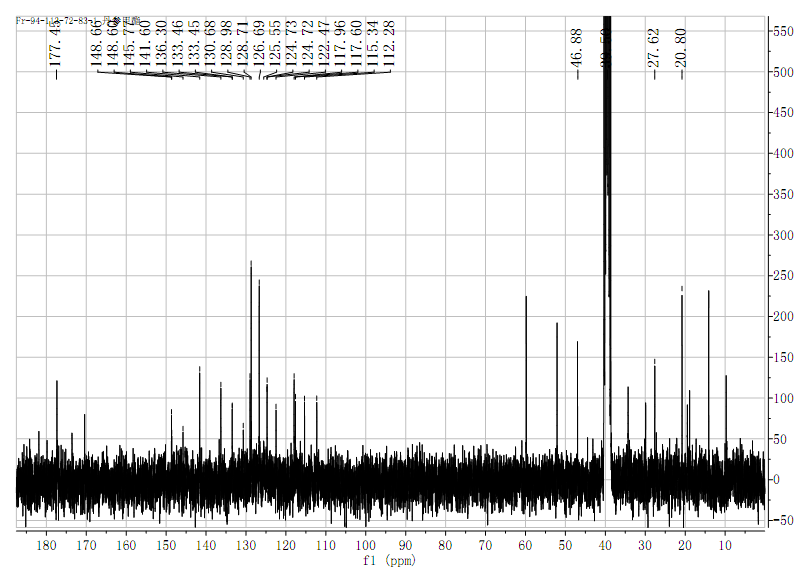
**

**Figure S9c in File S1. ^13^C NMR spectrum of compound 8 (75 MHz, DMSO-d_6_).**


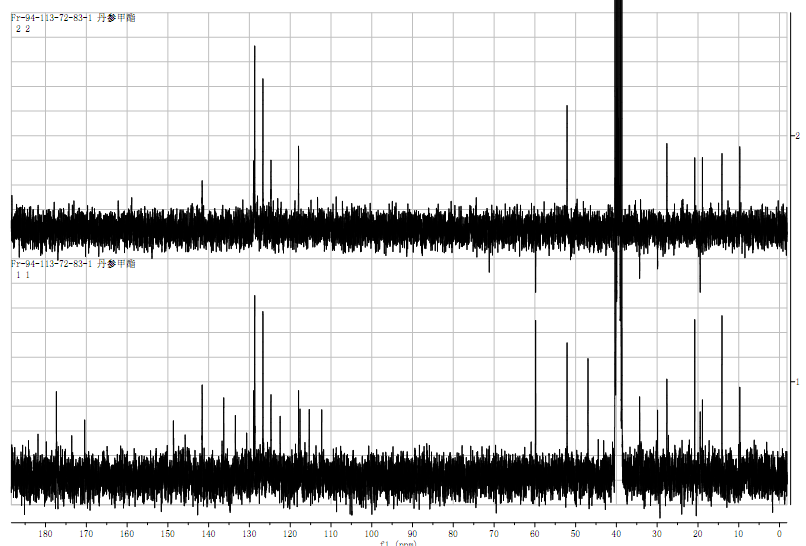


**Figure S9d in File S1. DEPT135 spectrum of compound 8 (DMSO-d_6_).**

**
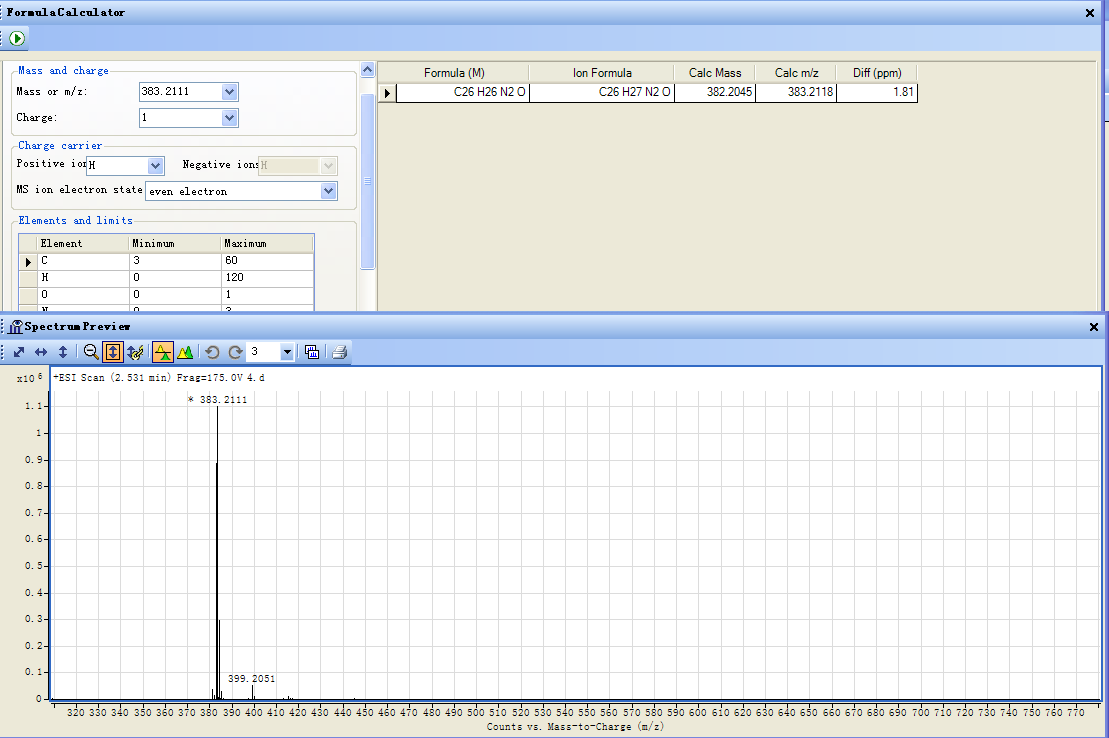
**

**Figure S10a in File S1. HR-ESI-MS of compound 9.**


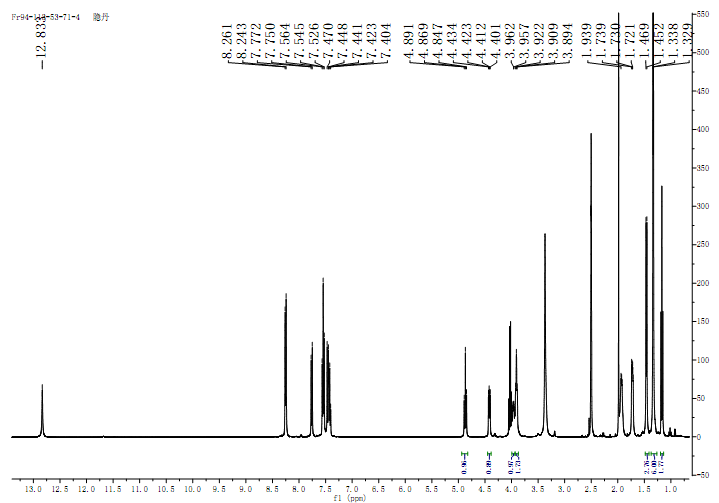


**Figure S10b in File S1. ^1^H NMR spectrum of compound 9 (300 MHz, DMSO-d_6_).**

**
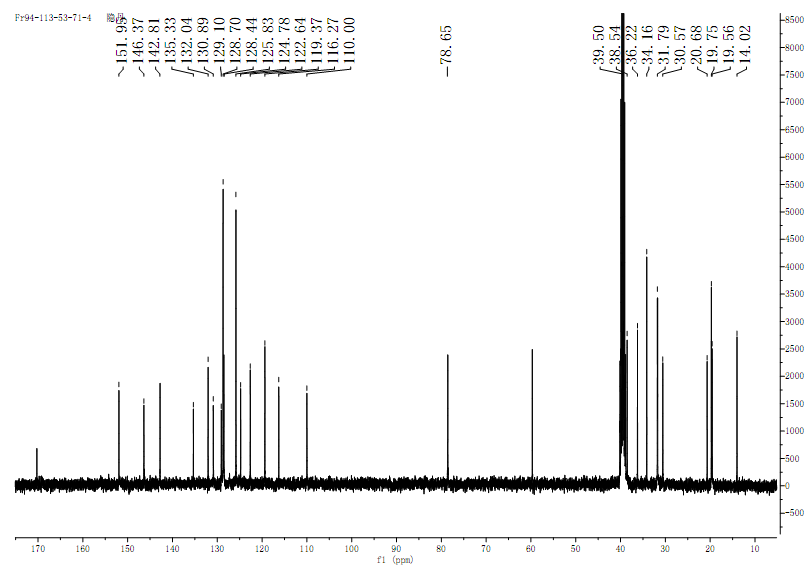
**

**Figure S10c in File S1. ^13^C NMR spectrum of compound 9 75 MHz, DMSO-d_6_).**


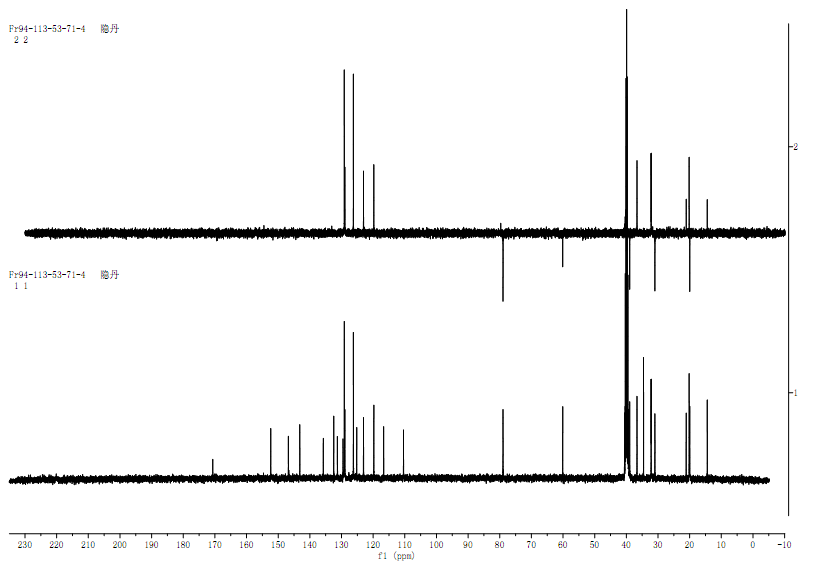


**Figure S10d in File S1. DEPT135 spectrum of compound 9 (DMSO-d_6_).**

**
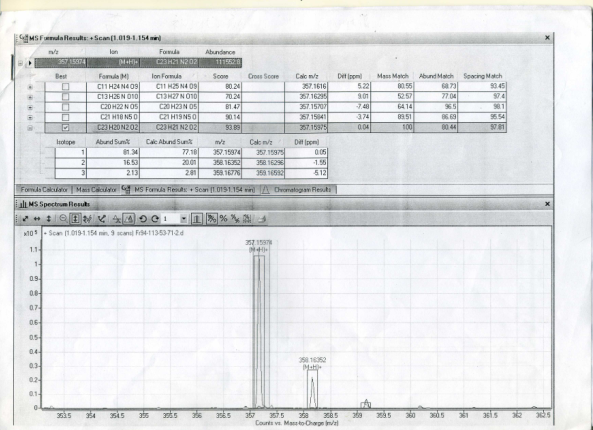
**

**Figure S11a1 in File S1. HR-ESI-MS of compound 10.**


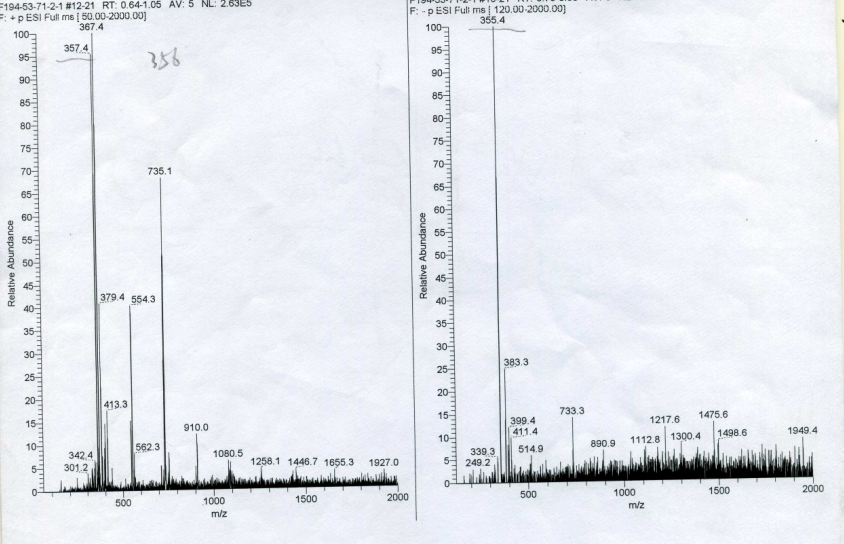


**Figure S11a2 in File S1. ESI-MS of compound 10 (left: positive; right: negative)**


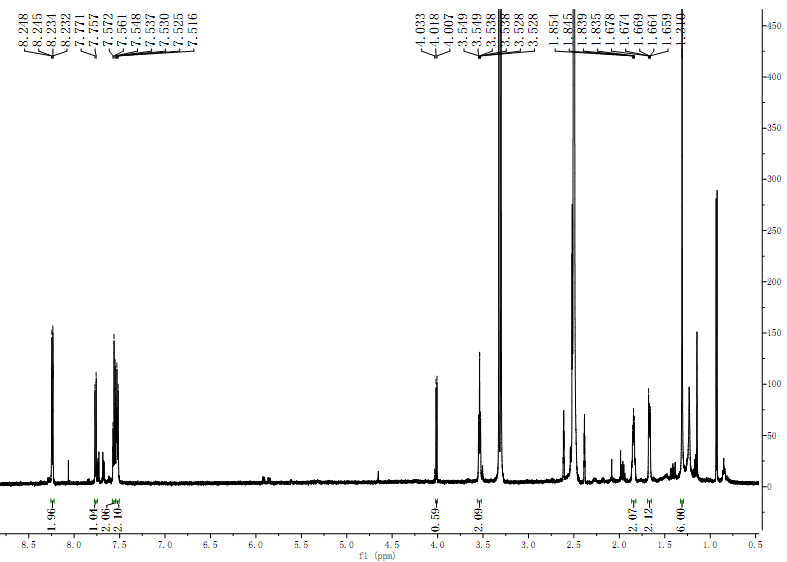


**Figure S11b in File S1. ^1^H NMR spectrum of compound 10 (300 MHz, DMSO-d_6_).**

**
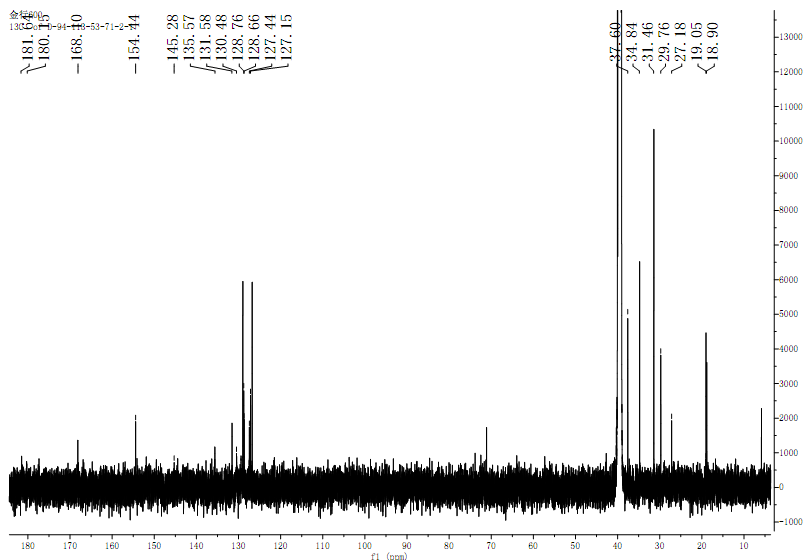
**

**Figure S11c in File S1. ^13^C NMR spectrum of compound 10 (75 MHz, DMSO-d_6_).**


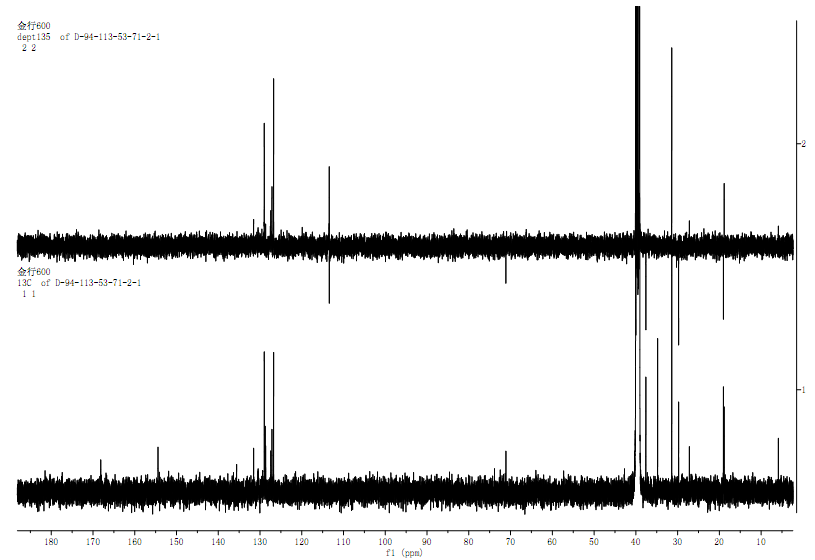


**Figure S11d in File S1. DEPT135 spectrum of compound 10 (DMSO-d_6_).**

**
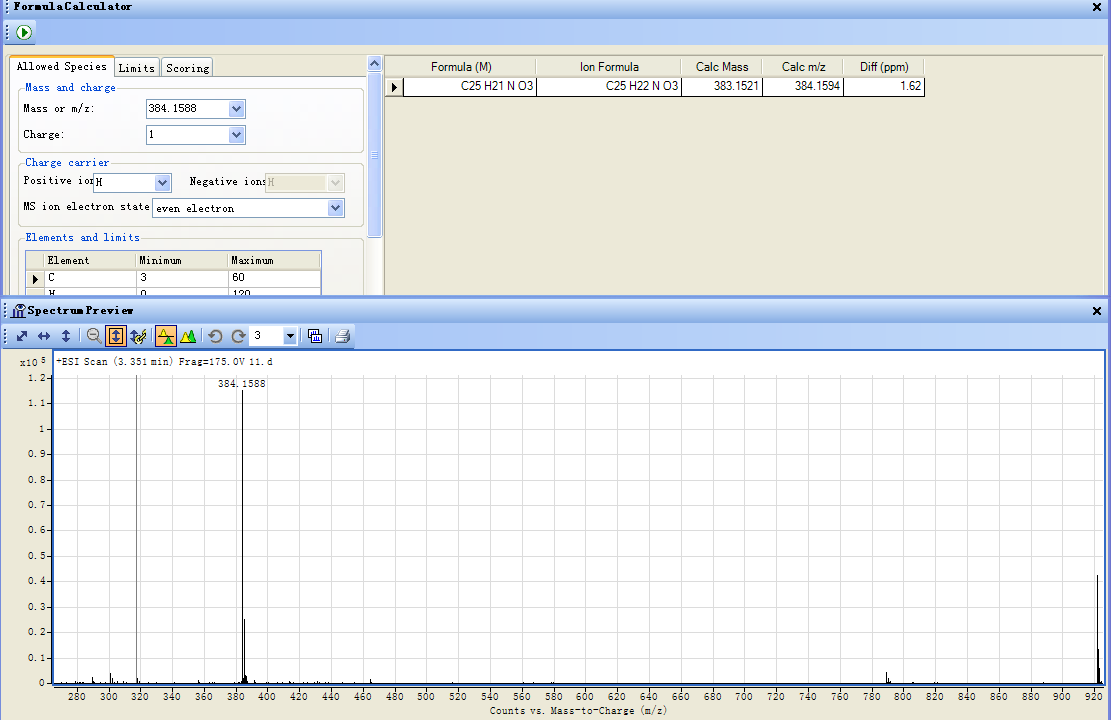
**

**Figure S12a in File S1. HR-ESI-MS of compound 11.**


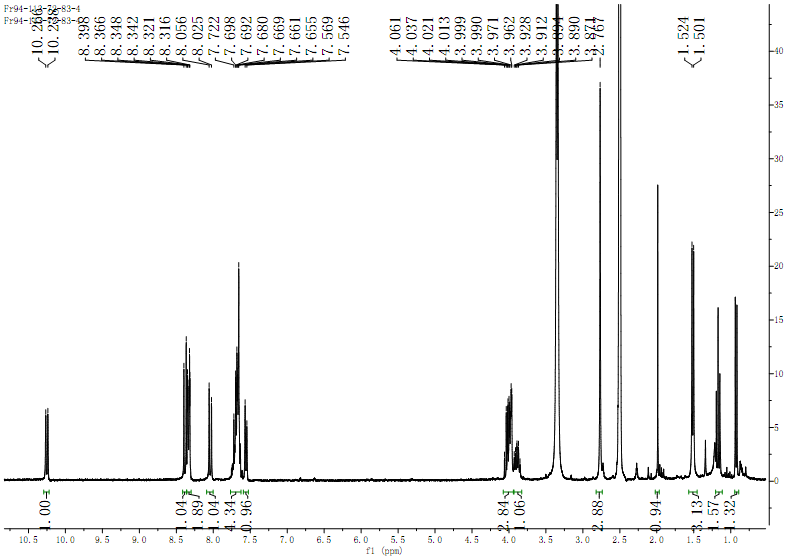


**Figure S12b in File S1. ^1^H NMR spectrum of compound 11 (300 MHz, DMSO-d_6_).**

**
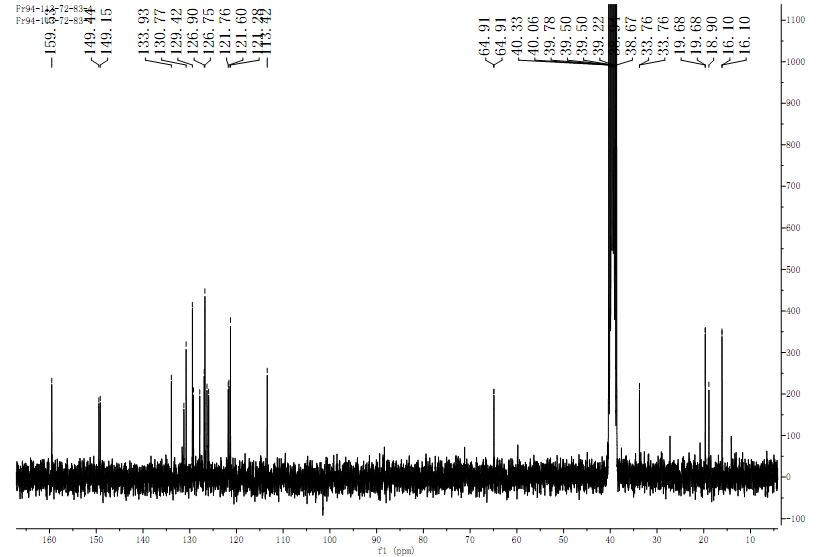
**

**Figure S12c in File S1. ^13^C NMR spectrum of compound 11 (75 MHz, DMSO-d_6_).**


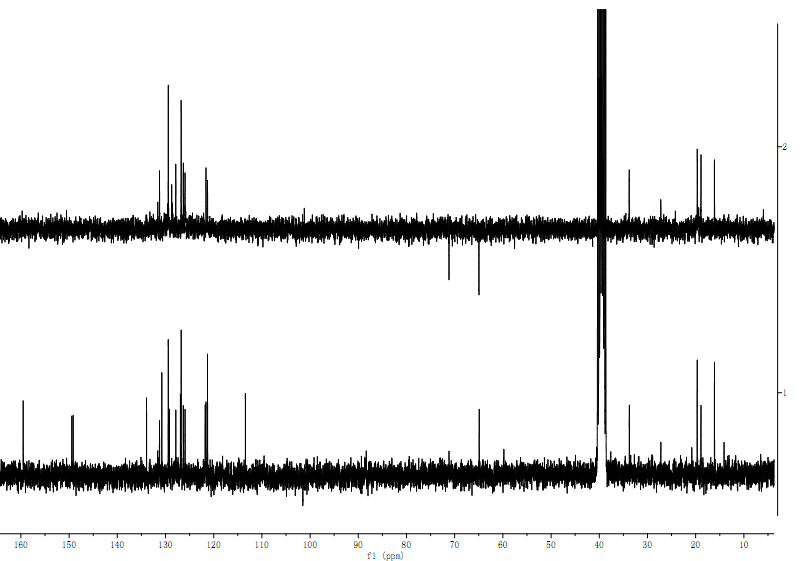


**Figure S12d in File S1. DEPT135 spectrum of compound 11 (DMSO-*d_6_*).**

**Section 4:**

**Table S1 in File S1.** Crystal data and structure refinement for compounds **1, 2, 3, 6 and 10**.

| **Compounds** | **1** | **2** | **3** | **6** | **10** |
| --- | --- | --- | --- | --- | --- |
| CCDC deposit no. | 899336 | 899337 | 899338 | 899339 | 899340 |
| Color/shape | Light red/block | yellow/ plate | light yellow/prism | yellow/prism | Orange/Prism |
| Cryst dimension (mm^3^) | 0.44×0.32 × 0.28 | 0.50 × 0.10 × 0.08 | 0.50× 0.12 × 0.10 | 0.32×0.12 ×0.08 | 0.4× 0.1 ×0.08 |
| Chemical formula | C_25_H_18_N_2_O | C_25_H_20_N_2_O | C_25_H_20_N_2_O | C_26_H_24_ N_2_O | C_23_H_20_N_2_O_2_ |
| Formula weight | 362.42 | 364.44 | 364.44 | 380.48 | 356.41 |
| Temperature, K | 293(2) | 293(2) | 293(2) | 293(2) | 293(2) |
| Crystal system | Triclinic | Orthorhombic | Orthorhombic | Orthorhombic | Monoclinic |
| Space group | *P-1* | *P2(1)2(1)2(1)* | *P2(1)2(1)2(1)* | *P bca* | *P2(1)* |
| Unit cell dimens (Å) | *a* = 7.712(4) | *a* = 5.230(10) | *a* = 6.679(10) | *a* = 7.614(1) | *a* = 11.7851(3) |
|  | *b* = 11.798(6) | *b* = 18.470(4) | *b* = 15.923(2) | *b* = 23.810 (1) | *b* = 5.97390(10) |
|  | *c* = 12.539(6) | *c* = 23.442(4) | *c* = 19.923(3) | *c* = 24.591(4) | *c* = 12.9667(3) |
|  | α= 98.528(4) |  |  |  |  |
|  | β=106.175(4) |  |  |  | β= 99.328(2) |
|  | γ= 96.093(4) |  |  |  |  |
| Volume, Å^3^ | 1070.42(9) | 2264.45(8) | 2118.85(5) | 4457.83(11) | 900.82(3) |
| *Z* | 2 | 4 | 4 | 8 | 2 |
| Density, Mg/m^3^ | 1.267 | 1.298 | 1.243 | 1.229 | 1.314 |
| Absorption coefficient, mm^-1^ | 0.635 | 1.478 | 0.624 | 0.61 | 0.674 |
| Diffractometer/ Scan | Oxford Gemini S Ultra CCD | Oxford Gemini S Ultra CCD | Oxford Gemini S Ultra CCD | Oxford Gemini S Ultra CCD | Oxford Gemini S Ultra CCD |
| θρανγε, δεγ | 5.83 to 62.56 | 3.00 to 61.08 | 3.55 to 62.62 | 3.59 to 60.82 | 3.45 to 62.67 |
| Reflections mesd | 5749 | 4380 | 4366 | 7613 | 2624 |
| Independent reflections (*R*_int_) | 3329 (0.0235) | 3044(0.0178） | 2856 (0.0328) | 3358 (0.0344) | 1981(0.0131) |
| Observed reflections [*I* > 2 *I*] | 4299 | 2836 | 2720 | 2439 | 1916 |
| Data/parameters | 3329 / 281 | 3044/290 | 2856 / 274 | 3358 / 281 | 1981/245 |
| Extinction coefficient | 0.004(8) | 0.0002(3) | 0.0065(6) | 0.0003(2) | 0.0013(4) |
| Goodness of fit on *F^2^* | 1.044 | 1.023 | 1.005 | 0.994 | 1.057 |
| *R*_1_[*I* > 2 (*I*)] | 0.065 | 0.0478 | 0.0475 | 0.0549 | 0.0272 |
| *wR*_2_ (all data) | 0.1828 | 0.1336 | 0.1331 | 0.1423 | 0.0704 |

**Section 5:** Structural Formulae of Natural Parental Tanshinones 1a-11a.

**Figure S13 in File S1. Structural formulae of natural parental tanshinones 1a-11a.**

**Section 6:** Configurations of Natural Parental Tanshinones 1a-11a. (All results were from Scifinder scholar database except for compound **5a** from literature)


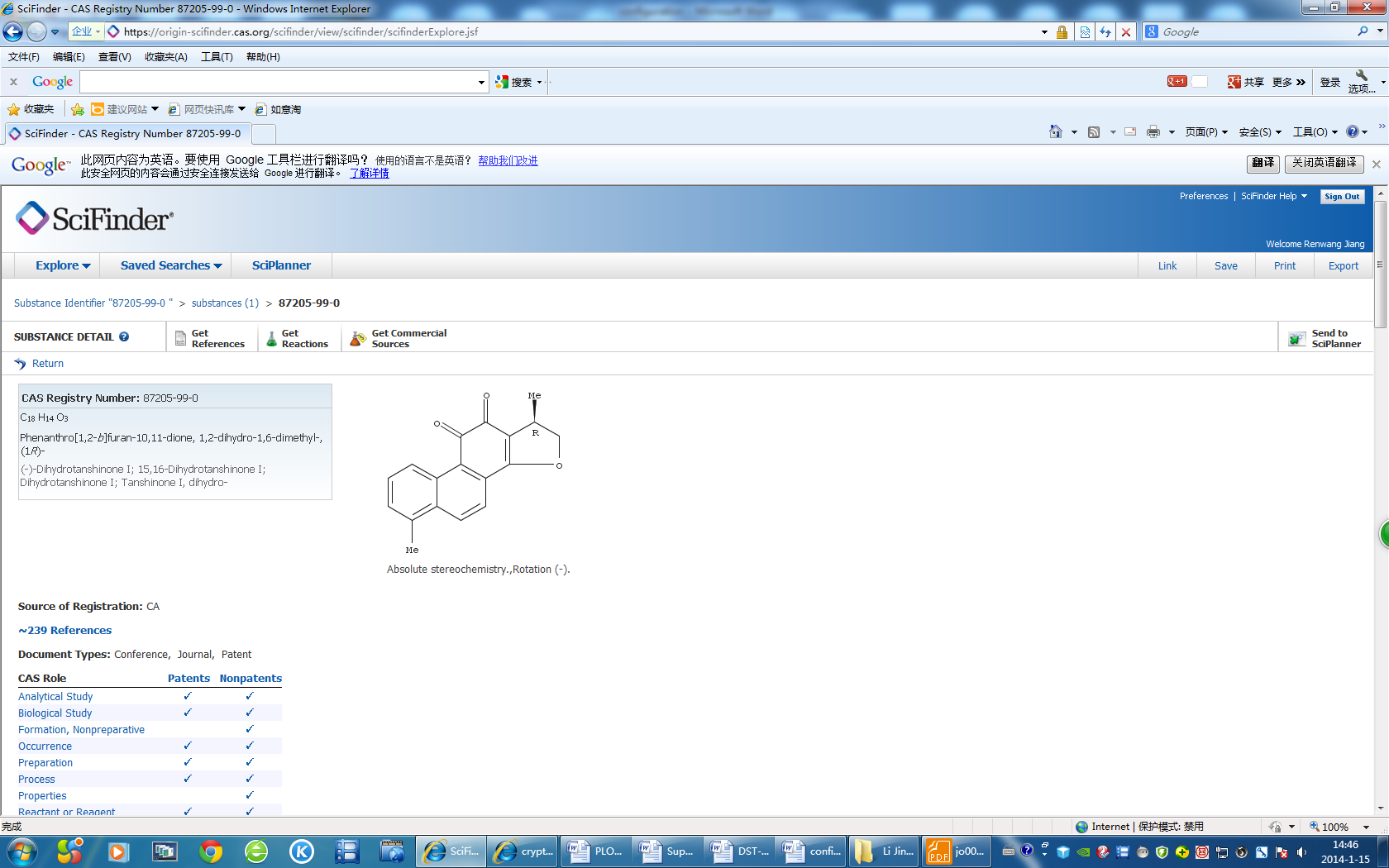


The know configuration of natural parental compound 15,16-dihydrotanshinone I (**3a**)

**
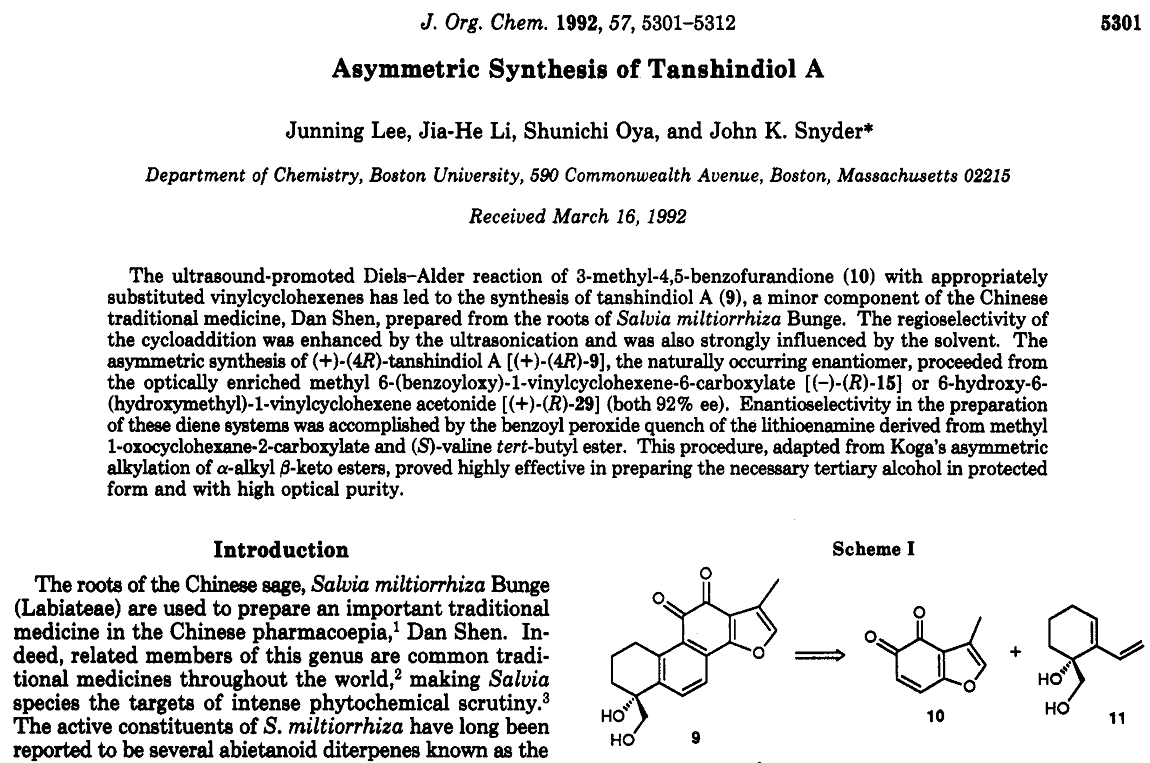
**

The know configuration of natural parental compound tanshindiol A (**5a**)


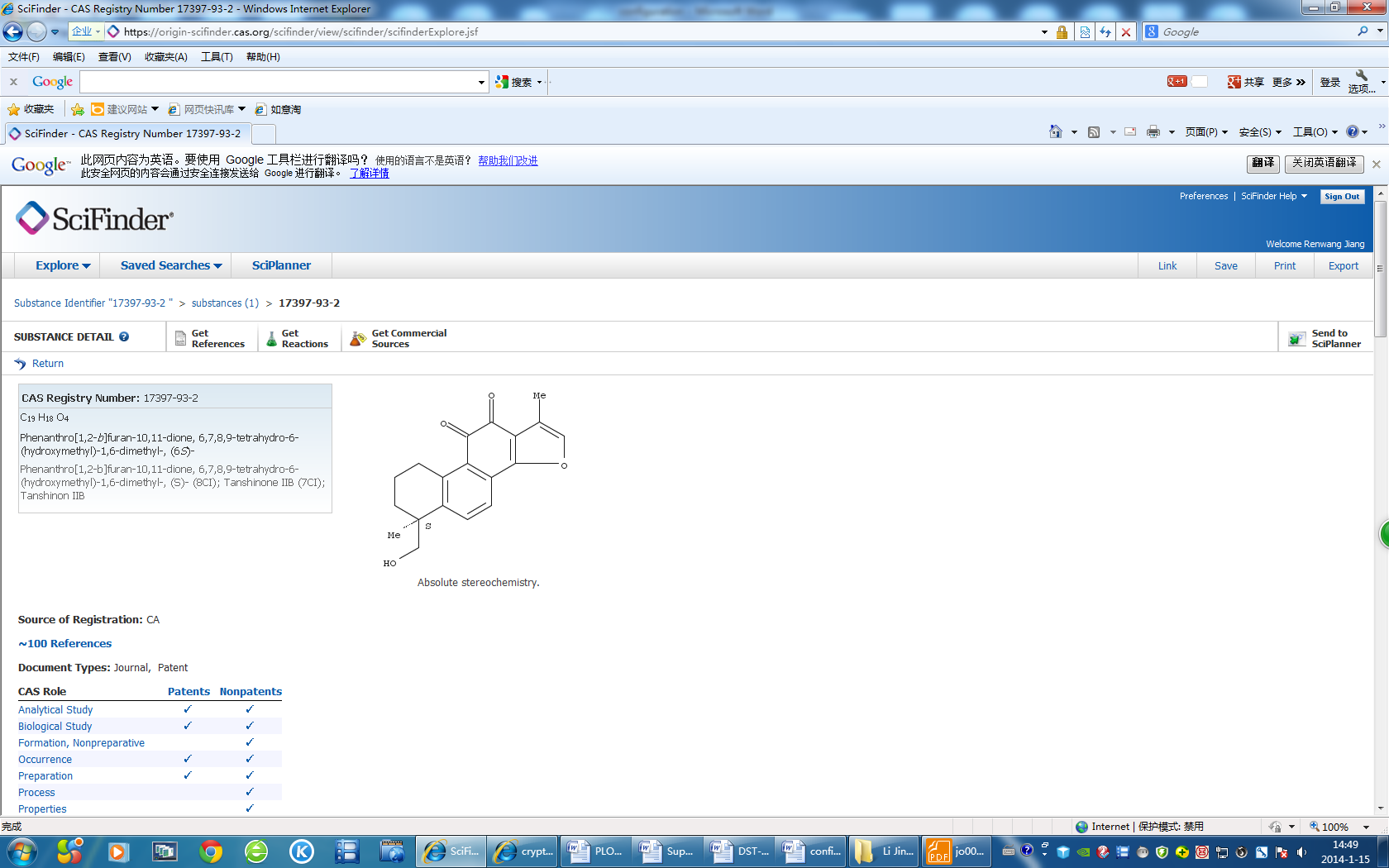


The know configuration of natural parental compound tanshinone IIB (**7a**)


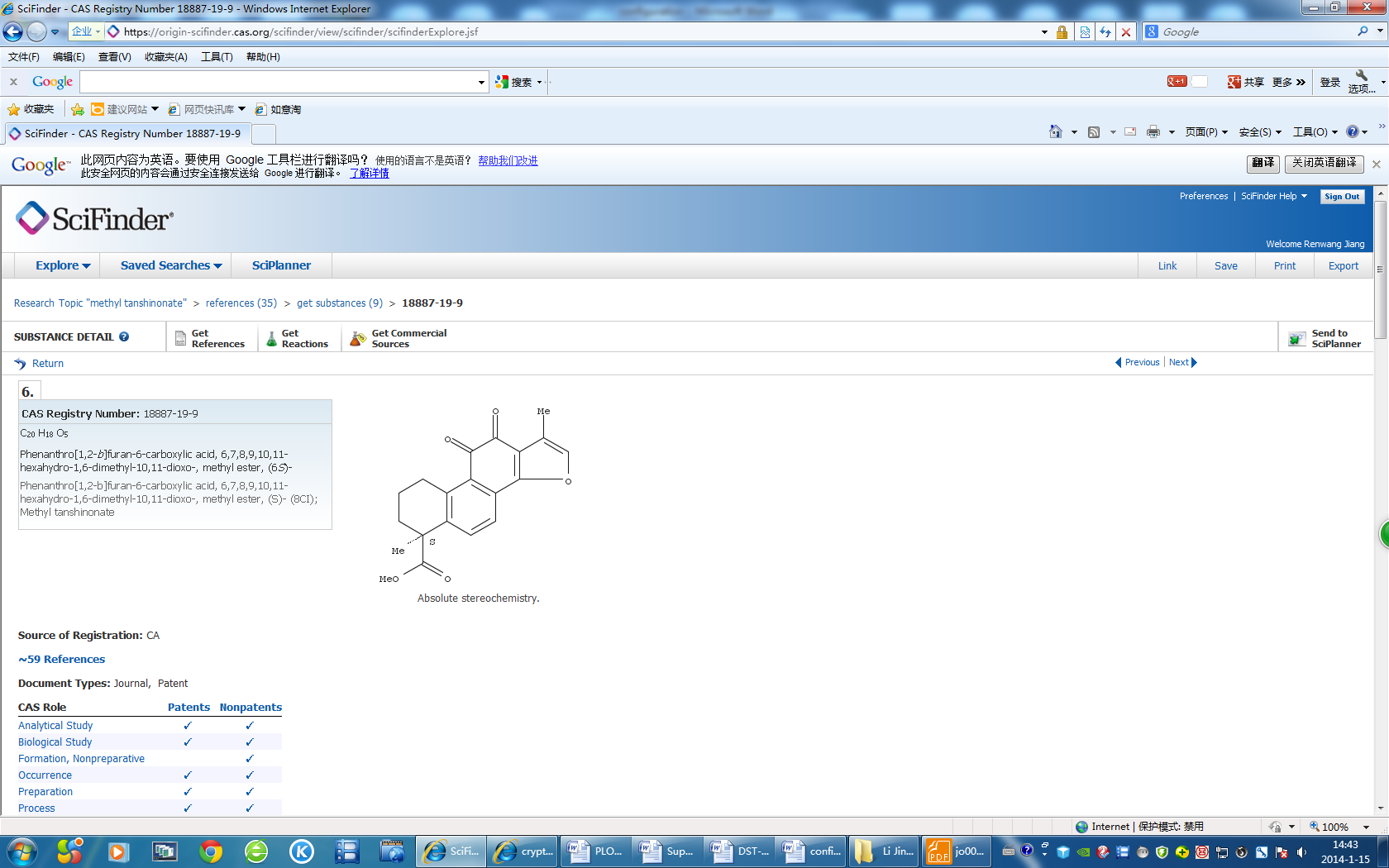


The know configuration of natural parental compound methyl tanshinonate (**8a**)


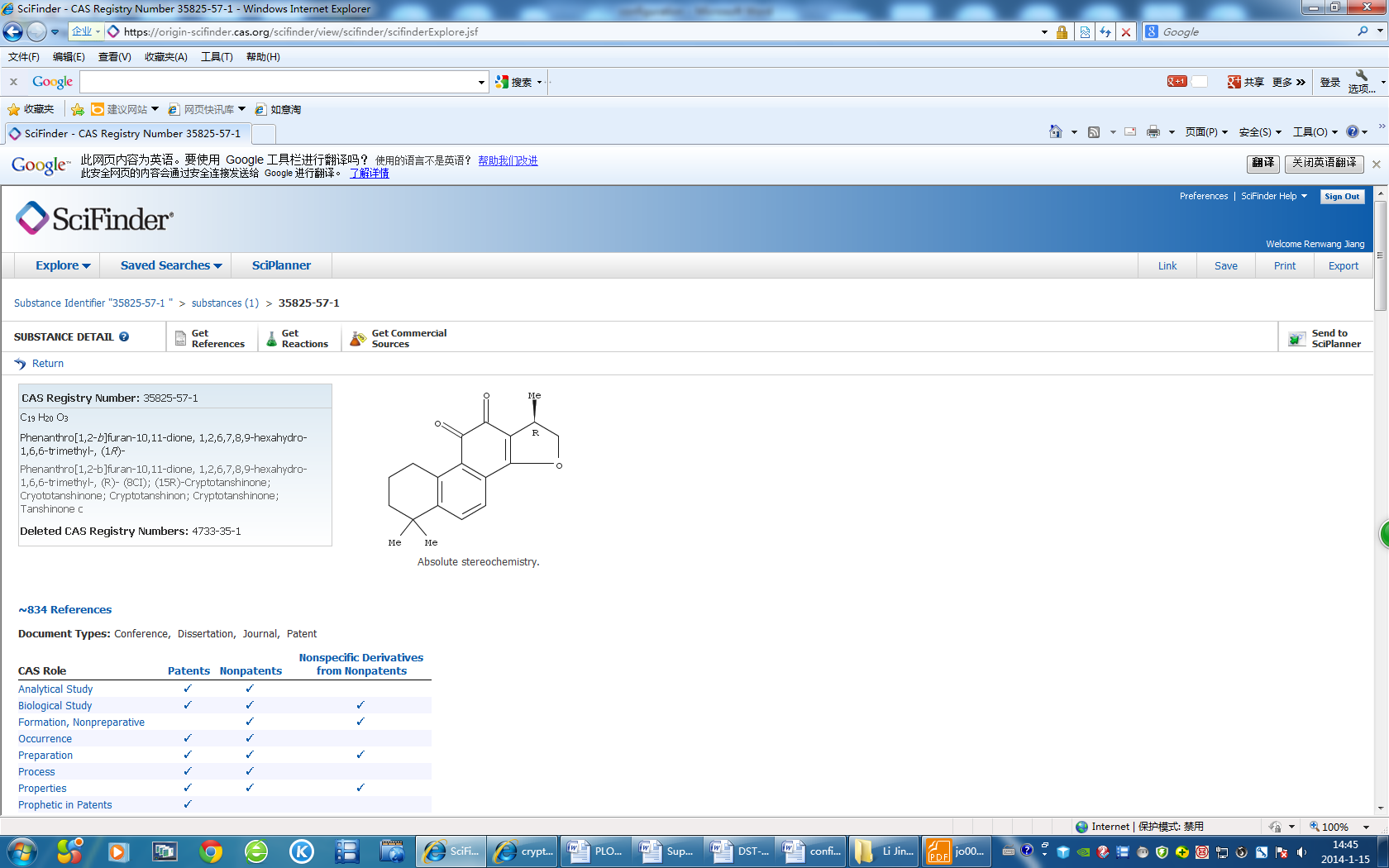


The know configuration of natural parental compound cryptotanshinone (**9a**)

**
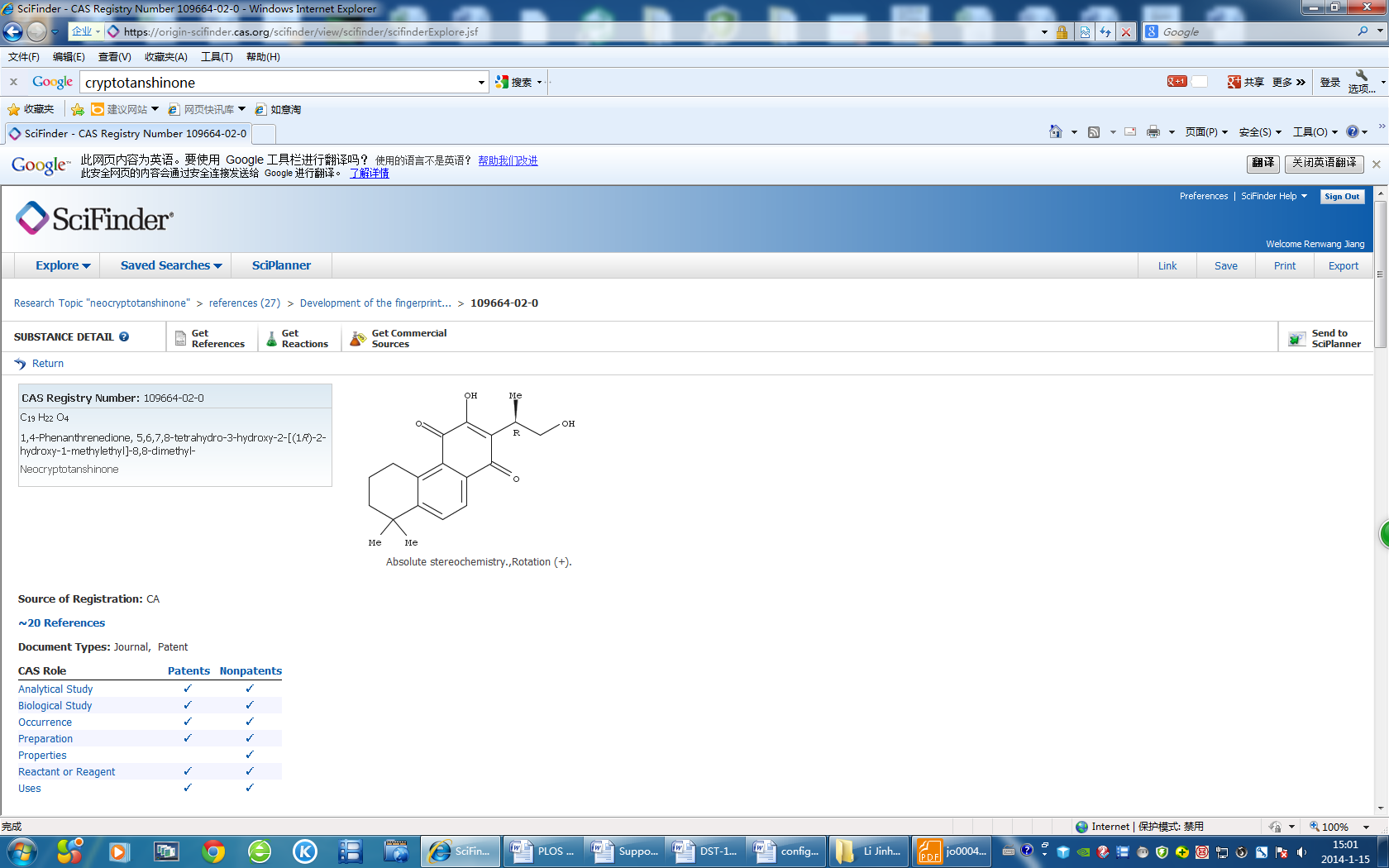
**

The know configuration of natural parental compound neocrypotanshinone (**11a**)

**Section 7:**

**Table S2 in File S1.** The EC_50_ and IC_50_ values of compounds **3**, **10**, Tan IIA, CPT and Tan I in VRI-treated zebrafish.

| **Compounds (μM)** | **3** | **10** | **Tan IIA** | **CPT** | **Tan I** |
| --- | --- | --- | --- | --- | --- |
| LC_50_ | 188.55 | 25.68 | 25.71 | 3.85 | 22.32 |
| EC_50_ | 4.03 | 0.026 | N/A^a^ | N/A^a^ | N/A^a^ |

LC_50_ (Lethal concentration, 50%) of compound is the dose required to kill half number of tested zebrafish embryos after 48 hpt.

EC_50_ (Half maximal effective concentration) refers to the concentration of compound which increases a halfway (50%) average number of ISVs for rescuing blood vessels loss compared to control group.

^a^Zebrafish embryos treated with compound at maximum non-toxic concentrations and the EC_50_ value was not available.

**Section 8:** Effects of Compound **10** on the Proliferation, Migration and Invasion of HUVECs.

The effect of compound **10** on HUVECs proliferation was evaluated by XTT assay. Following the 24 h starvation, HUVECs were cultured in low serum medium (0.5 % FBS) supplement with **10** (0.03-10µM) for 48 h. As shown in Fig. S14, although **10** significantly promoted HUVECs proliferation, the cell proliferation stimulating effect of **10** was mild compared with that of VEGF. The maximum increase of cell viability induced by **10** was 26.7% at 1 μM, compared to vehicle control. A significant (p<0.001) increase in cell proliferation was also observed in VEGF-treated group (89%), which served as the positive control.


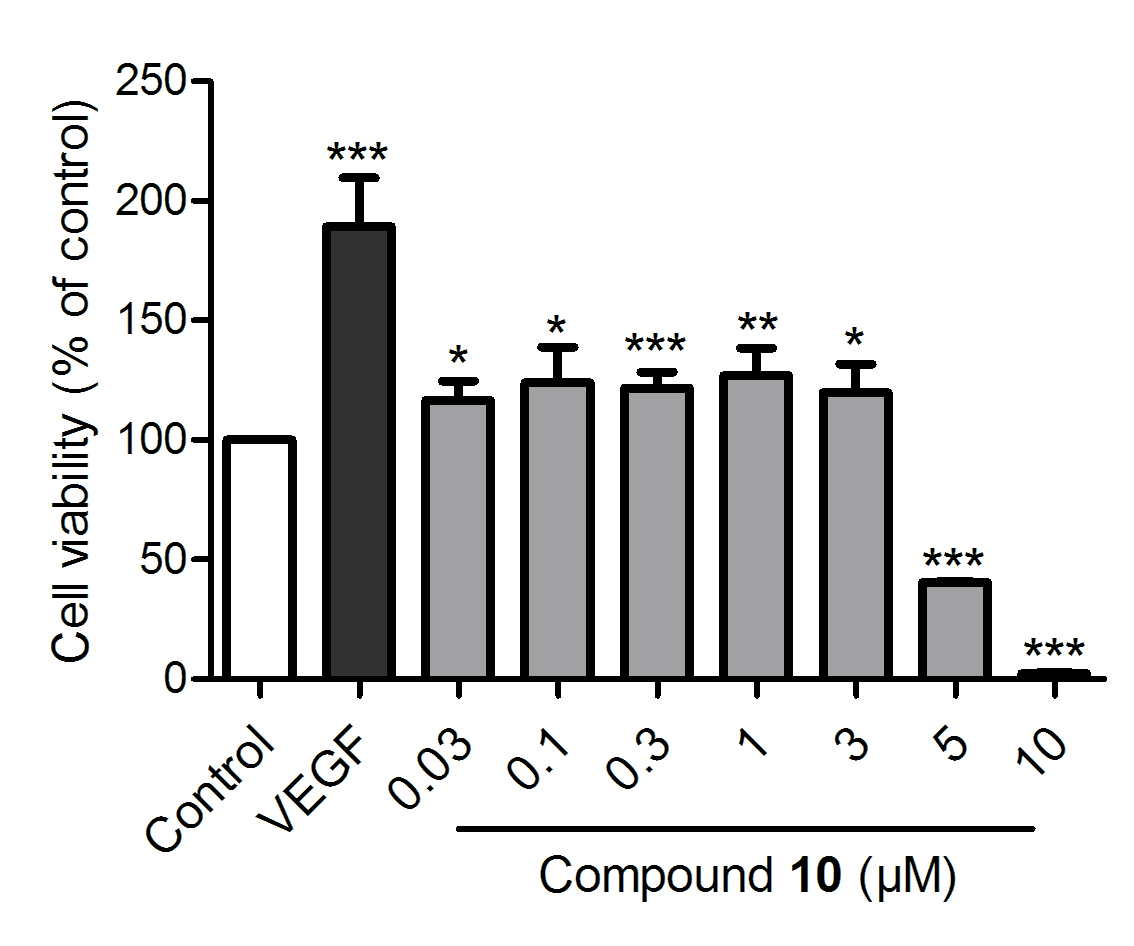


**Figure S14 in File S1. The effect of compound 10 on HUVECs proliferation.**

HUVECs were serum-starved for 24 h followed by incubation with **10**. Data are expressed as the percentage cell viability ± SEM of duplicate experiments. *p<0.05, **p<0.01 and ***p<0.001 versus vehicle control (0.1% DMSO).

The effect of **10** on HUVECs migration was examined using the wound healing method. As shown in Fig. S15, a low level of HUVECs migration was observed in the vehicle control group after 18 h wounding. In contrast, a significant (p<0.001) increase to 208% in cell number of migration was observed in VEGF-treated group which served as the positive control compared to the vehicle. Meanwhile, **10** had been exhibited obvious increase in HUVECs migration in a concentration-dependent manner and the most significant concentration of cell migration stimulation (207%) was 0.3 μM.


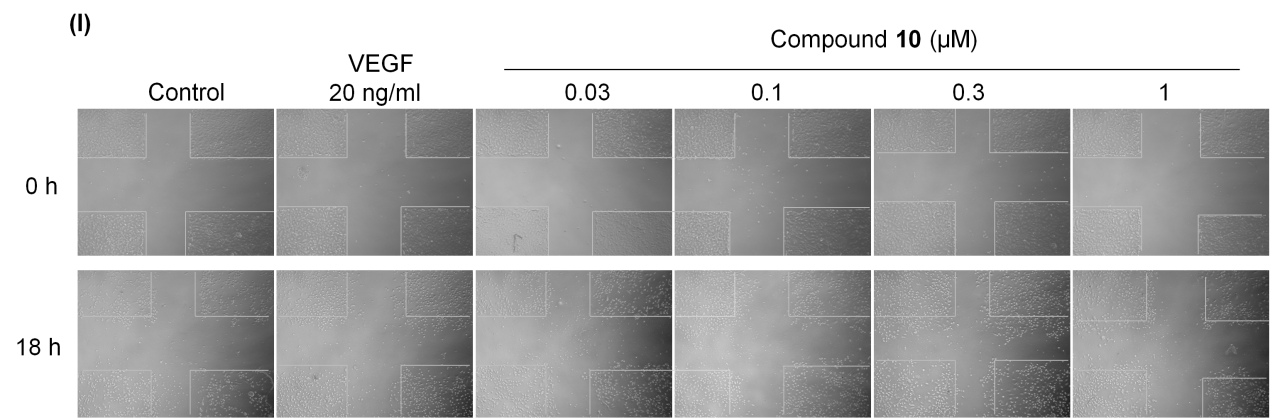


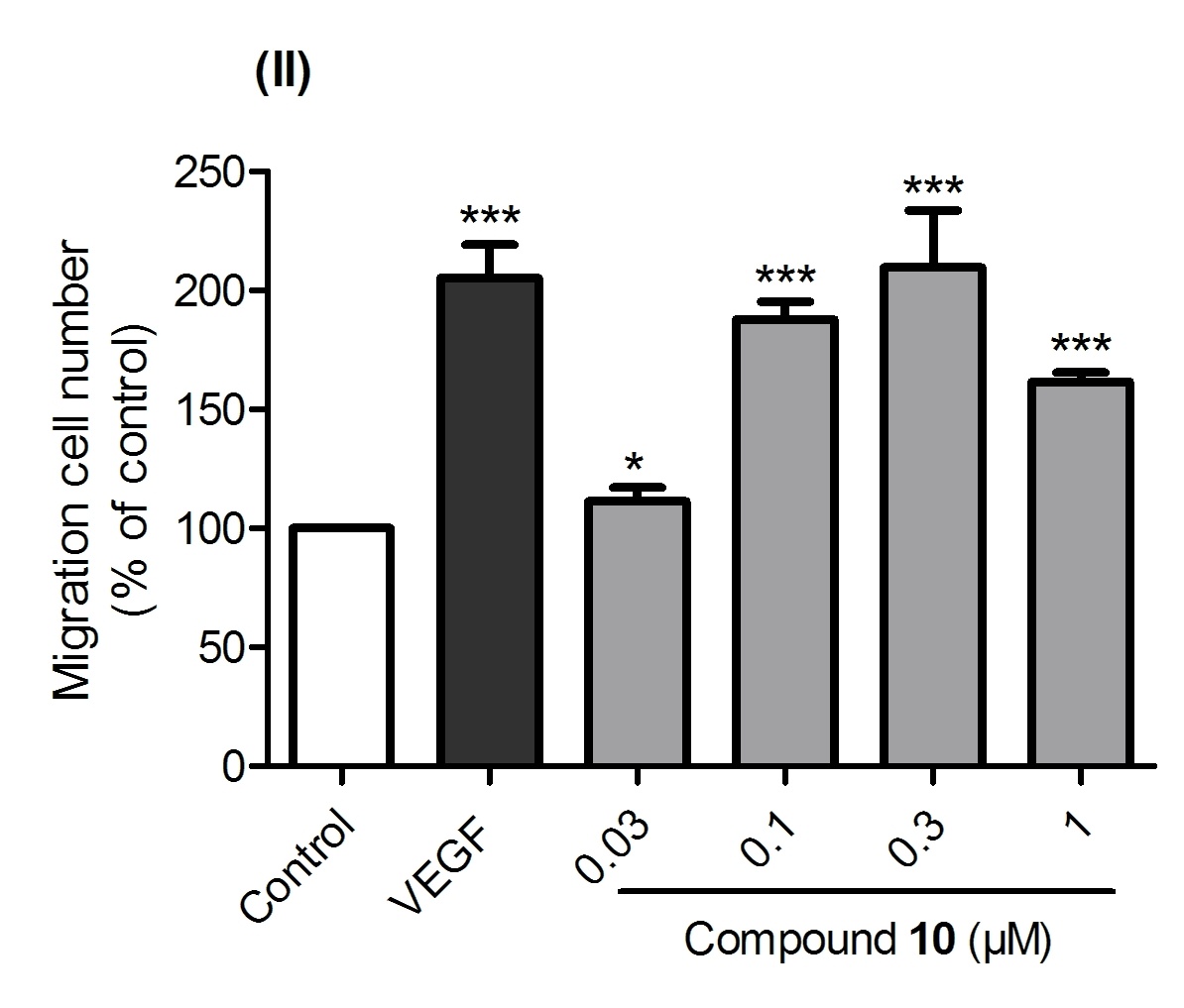


**Figure S15 in File S1. The effect of compound 10 on HUVECs migration.**

(I)HUVECs were serum-starved for 24 h, then immediately after wounding cells were incubated with **10** for 18 h.

(II) The extent of migration was determined by averaging migration cell number after treatment. Values are given as the increment of migration ± SEM, for three independent experiments. *p < 0.05, **p<0.01 and ***p<0.001 versus vehicle control (0.1% DMSO).

To test the ability of **10** to induce HUVECs invasion, an *in vitro* Matrigel model was used. In this assay, HUVECs were stimulated to invade through an extracellular matrix and migrate toward an angiogenic chemoattractant. As positive control, the VEGF-treated cells significantly enhanced the invasion (237%) (Fig. S16). Compared with the vehicle control, **10** stimulated invasion of HUVECs in a dose-dependent manner following 7 h incubation, and reached a maximum effect (197%) at a concentration of 0.3 μM. These results indicated that **10** was capable of inducing the HUVECs invasion.


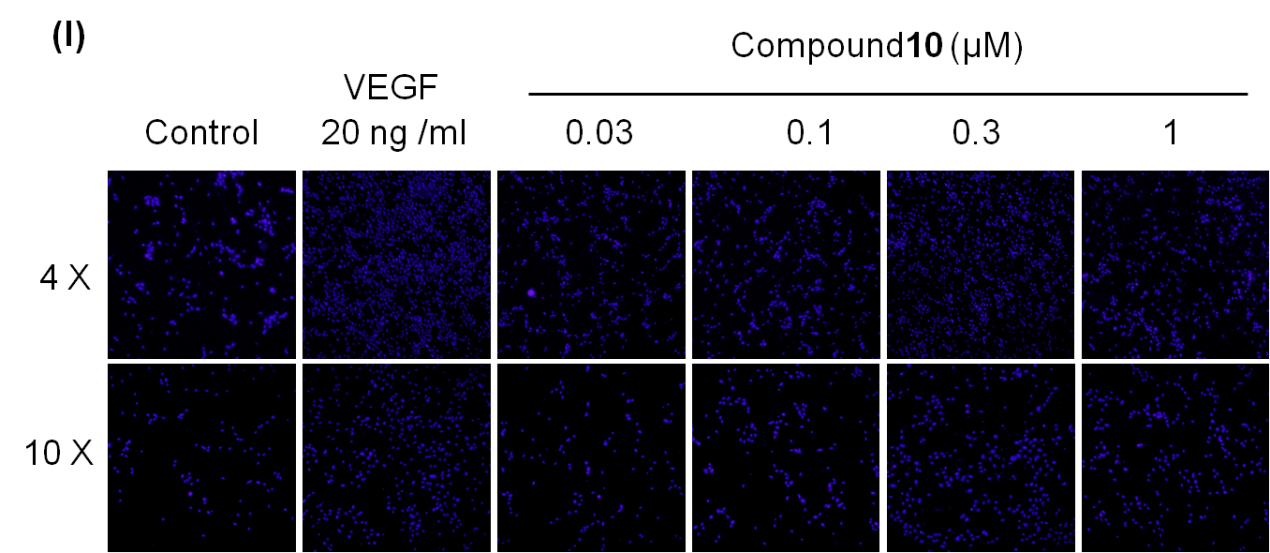


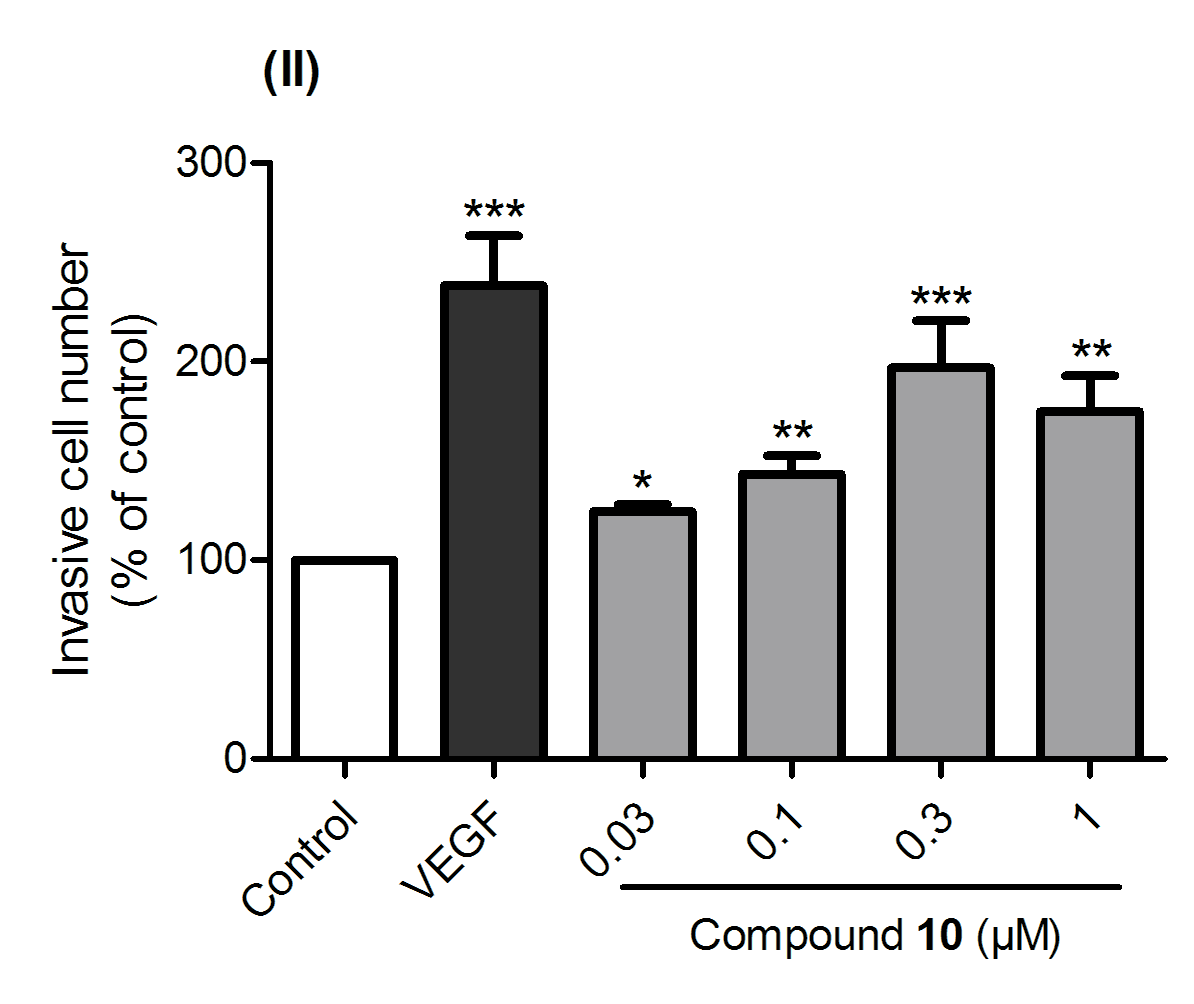


**Figure S16 in File S1. The effect of compound 10 on HUVECs invasion.**

(I) HUVECs were seeded onto inserts pre-coated with the Matrigel and placed into the 24-well companion plates with medium containing **10**. After 7 h, the inserts were removed, washed with PBS and non-invading cells on the upper surface of the membrane were removed by wiping with cotton swabs. The membranes were stained with Hoechst 33342 and captured by the fluorescent inverted microscope (at 4× and 10×).

(II) Quantitative analysis of the **10**-induced HUVECs invasion was carried out using the Metamorph Imaging Series software. Data are expressed as the increment of invasion ± SEM from three individual experiments. *p< 0.05, **p<0.01 and ***p<0.001 versus vehicle control (0.1% DMSO)
